# Brain miR-137 governs growth and development via GH/IGF-1 signaling

**DOI:** 10.1186/s12915-025-02306-8

**Published:** 2025-07-01

**Authors:** Keng-Mao Liao, Wei-Lun Hsu, Wan-Yi Huang, Wei-Jia Luo, Jung-Hsuan Chang, Sung-Liang Yu, Pan-Chyr Yang, Kang-Yi Su

**Affiliations:** 1 Department of Clinical Laboratory Sciences and Medical Biotechnology, College of Medicine, National Taiwan University, Taipei, 100233 Taiwan; 2 Department of Internal Medicine, College of Medicine, National Taiwan University, Taipei, 100233 Taiwan; 3 Centers for Genomic and Precision Medicine, National Taiwan University, Taipei, 100025 Taiwan; 4 Department of Laboratory Medicine, National Taiwan University Hospital, Taipei, 100233 Taiwan; 5 College of Medicine, Graduate Institute of Pathology, National Taiwan University, Taipei, 100233 Taiwan; 6 Genome and Systems Biology Degree Program, National Taiwan University, Taipei, 106319 Taiwan

**Keywords:** MiR-137, Growth retardation, IGF-1, GH resistance, Knockout mice

## Abstract

**Background:**

Brain-enriched miR-137 is highly associated with neuropsychiatric disorders and neural development. Although complete loss of miR-137 that leads to postnatal lethality had been addressed in mice, the underlying mechanism particularly related to growth and development remains unknown.

**Results:**

MiR-137-deficient mice (*Mir137*^−/−^) exhibited postnatal lethality, severe growth retardation, osteoporosis, fat atrophy, and hypothermia. Despite comparable serum growth hormone (GH) levels, IGF-1 levels in both liver and serum were significantly reduced, with compensatory upregulation of IGF-1 receptor expression in major organs. Reduced IGF-1 levels were not due to defects in GH secretion by the pituitary nor GH responsiveness of hepatocytes. Instead, impaired in vivo GH-induced p-STAT5 signaling suggested GH resistance in *Mir137*^−/−^. Conditional deletion of *Mir137* in the nervous system, but not in the liver, showed similar results, confirming the brain-specific role of miR-137. Transcriptomic analyses revealed that differentially expressed genes in the brain were enriched in development and neurogenesis while those in the liver showed diverse and less enrichments. IGF-1 reduction caused by miR-137 deficiency emerged as a central factor impacting the cell proliferation network to systemic growth.

**Conclusions:**

This study underscores the critical role of miR-137 in failure to thrive through regulation of the GH/IGF-1 axis and supports the use of *MiR137*^−/−^ as a disease model for GH resistance. Given the conserved miR-137 sequences between mice and humans, further human studies or clinical trials may validate its potential as a biomarker and therapeutic target for growth retardation.

**Supplementary Information:**

The online version contains supplementary material available at 10.1186/s12915-025-02306-8.

## Background

MicroRNAs (miRNAs) are small non-coding RNA composed of 18–22 nucleotides that are derived from longer primary miRNA transcripts and processed by serial enzymatic activities such as Drosha and Dicer [1]. They are usually clustered within both inter-genic and intra-genic region of genome and transcribed in polycistronic transcripts. The expression pattern differed depending on different tissues and changes of miRNAs may represent pathological condition of specific origins [2]. Upon binding to corresponding target mRNAs containing the complementary sequence of seed region, miRNAs can regulate genes by either repressing translation or degrading their mRNAs [3]. One miRNA often targets multiple genes while a gene may, in turn, be regulated by multiple miRNAs. Therefore, the regulatory network between miRNA and genes may be complicated for pathogenesis of disease.

The brain was a complex organ responsible for controlling physiological function when facing internal and external stimulations. According to previous studies, although more than 70% miRNAs were expressed in the brain, only a very small proportion of miRNAs were brain-specific or brain-enriched [4, 5]. Accumulated evidences showed brain-enriched miRNAs were responsible for growth and development. In the mention of cardiovascular development, miR-128 had been reported to promote differentiation of cardiomyocyte progenitor cells for early heart development. miR-138 was necessary for the appropriate patterning of heart chamber while miR-218 was shown to be a vital factor for the heart tube formation [6–8]. Regarding skeletal and bone formation, miR-140 deficiency led to shortened limbs and miR-206 conferred to muscle development through regulating connexin43 [9, 10]. MiR-124 contributed to many aspects related to growth and development in the brain, such as cell proliferation, migration, memory formation, and neurodegeneration [11].

Mir-137 is also a brain-enriched microRNA with a highly conserved sequence across various species [12]. Based on target prediction analysis, miR-137 is potentially involved in cellular functions, metabolism, growth and development, and biological regulation [13]. Functional genomics and clinical studies have shown a strong association between miR-137 and neurological or psychiatric disorders. MiR-137 plays a crucial role in neural development and is also associated with schizophrenia [14–16]. Moreover, a significant downregulation of miR-137 has been observed in young patients with Parkinson’s disease [17]. Recently, miR-137 is reported it may mitigate autism-like behaviors through HIF-1α-dependent modulation of glycolysis and neuroinflammatory pathway [18]. The in vivo physiological role of miR-137 was also demonstrated by Cheng et al. using a gene-targeted mouse model [19]. Mice heterozygous for miR-137 deficiency exhibited neurological and cognitive abnormalities, including dysregulated synaptic plasticity, repetitive behavior, and impaired learning and memory. Notably, they also reported that complete whole-body or nervous system-specific loss of miR-137 resulted in reduced body and brain weights and postnatal lethality. However, the causal relationship between miR-137 deficiency and growth retardation had not been addressed. This prompted us to investigate the regulatory role and underlying mechanisms of miR-137 in systemic growth and development. Although organs such as the brain and liver are well-documented to participate in endocrine regulation of growth [20], few studies have explored the connection between brain-enriched microRNAs, growth retardation, and hormone signaling. We hypothesize that brain-specific miR-137 may regulate systemic growth and development through endocrine signaling. Accordingly, we generated miR-137-deficient mice (*Mir137*^−/−^) to explore the underlying mechanisms.

In the present study, we found mice deficient in whole body and nervous system specific miR-137, but not liver-specific miR-137, exhibited severe growth retardation and postnatal lethality. While serum growth hormone (GH) levels were comparable in wild-type mice (*Mir137*^+/+^) and *Mir137*^−/−^, the levels of IGF-1 in both liver and serum were significantly reduced in *Mir137*^−/−^, suggesting impaired hepatic IGF-1 synthesis. This defect was not due to insufficient GH secretion from the pituitary or impaired GH responsiveness in hepatocytes, resembling features of GH resistance. Transcriptomic and pathway analyses suggested that growth retardation in *Mir137*^−/−^ may result from systemic network dysregulation involving miR-137 target genes. In particular, IGF-1 was identified as a central molecule connecting cell proliferation signaling to systemic growth. Taken together, our findings reveal a novel link between miR-137, growth retardation, and GH/IGF-1 axis signaling. Whether miR-137 could serve as a growth-related biomarker or therapeutic target warrants further investigation.

## Results

### Mice deficient in miR-137 exhibit severe growth retardation and cause postnatal early lethality

**Fig. 1:**
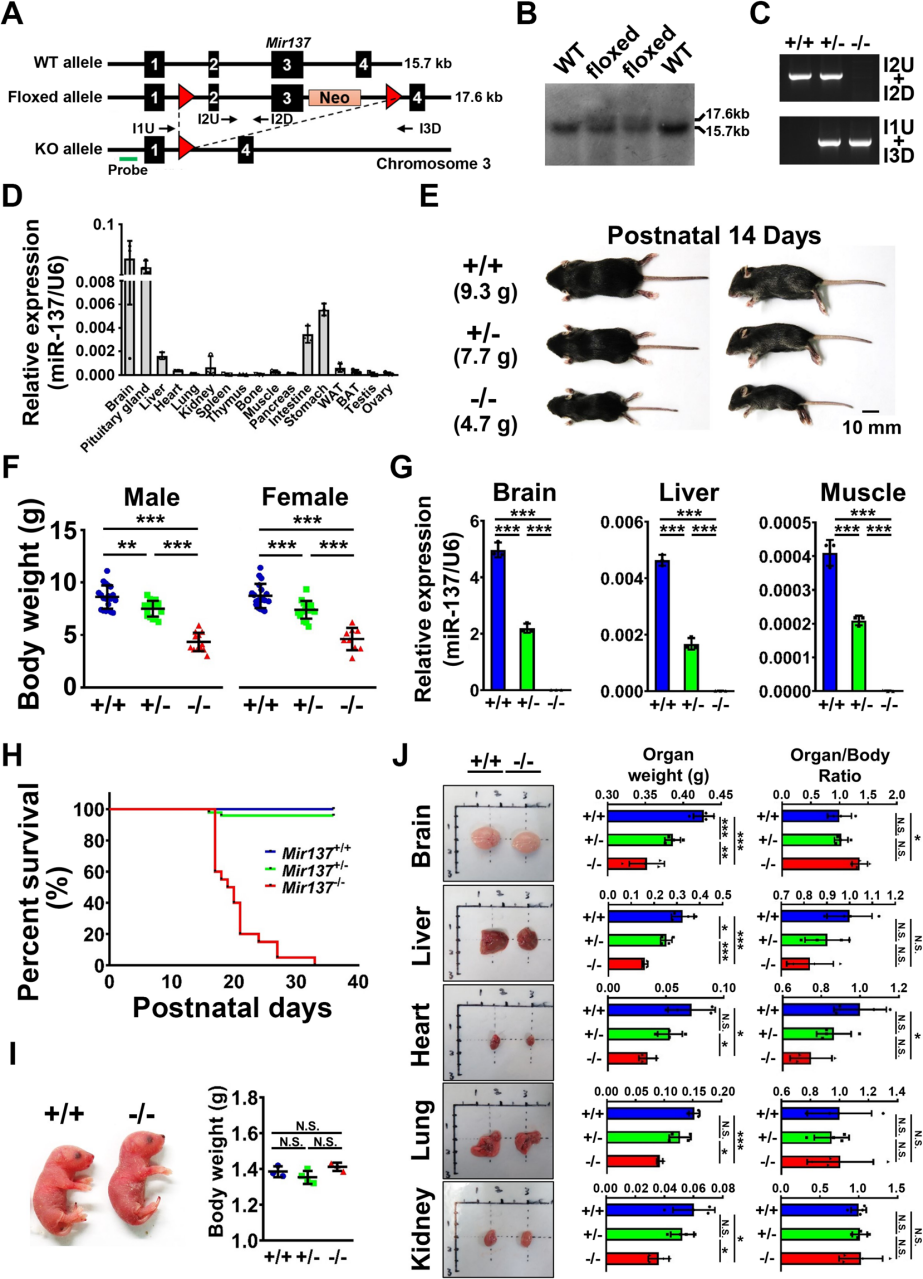
Mice deficient in miR-137 exhibited severe growth retardation and postnatal lethality. **A** Targeting strategy. The 12.6 kb targeting fragment containing loxP (the red triangle)-floxed mouse Mir137 locus containing exon 1–4 (numeric black squares) locus was used to replace endogenous wild-type Mir137 allele by homologous recombination. With introducing Cre recombinase, the exons 2–3 containing mature miR-137 were deleted to generate the knockout allele. The green line represented the probe for Southern blot and arrows represented primers for PCR genotyping. **B** Embryonic stem cells with *lox*P-floxed allele were confirmed by Southern blot. The molecular size was 15.7 kb for wild-type alleles and 17.6 kb for loxP-floxed alleles. **C** Genotyping by PCR. Primer pairs I2U plus I2D and I1U plus I3D were used to check wild-type and knockout alleles, respectively. PCR products were 580 bp in size for wild-type and 600 bp in size for knockout alleles. **D** Expression profiling of miR-137 by Taqman RT-PCR (*n* = 3 for each organ). The *U6* was used as internal control for normalization. **E** Photographs of wild-type (+/+, *n* = 3), *Mir137* heterozygous knockout mice (+/−, *n* = 4), and *Mir137* homologous knockout mice (−/−, *n* = 3) at postnatal day 14. **F** Quantification of body weights of +/+, +/−, and −/− mice at postnatal day 14 (male, *n* = 19, 17, 13; female, *n* = 19, 17, 9). **G** miR-137 expression in brains, livers, and muscles of each genotype by Taqman RT-PCR. The *U6* was used as internal control for normalization (*n* = 4 for each group). **H** Survival curve of +/+, +/−, and −/− mice from the date of birth (*n* = 35, 48, and 20 for +/+, +/−, and −/−, respectively). **I** Left panel, the photograph of +/+ and −/− neonatal mice (postnatal day 1); right panel, quantification of body weights (*n* = 3–4 for each group). **J** Macroscopic analysis of major organs. Left panel, photographs of major organs from +/+ and −/− mice; right panel, quantification of organ weights and organ/body ratios in +/+, +/−, and −/− mice. (*n* = 3–4 for each group) Data was presented as the mean ± SD. *p < 0.05; **p < 0.01; ***p < 0.001 by Student’s *t* tests; N.S., non-significant

The targeting vector was designed to insert one *lox*P site upstream of exon 2 and a neomycin resistant gene with a *lox*P site downstream of exon 3 which encoded miR-137 from the mouse predicted EST-based gene (ENSMUSESTT00000017155) locus on chromosome 3 (Fig. 1A). By introducing Cre recombinase in embryonic stem (ES) cells, we deleted *Mir137* in the germline and generated heterozygous *Mir137* whole body knockout mice. Mir-137 wild-type (*Mir137*^+/+^), heterozygous (*Mir137*^+/−^), and homozygous (*Mir137*^−/−^) mice were generated by *Mir137*^+/−^ mating with each other. After homologous recombination in ES cells, the *lox*P-floxed allele of *Mir137* was identified by the presence of a 17.6-kb *Avr*II-*EcoR*V fragment while the wild-type allele had a 15.7-kb fragment (Fig. 1B) in Southern blot analysis by using the probe (Fig. 1A). The genotype of mice was performed routinely by primer pairs I1U + I3D and I2U + I2D for knockout and floxed alleles identification, respectively (Fig. 1C). To guide the following analysis for physical developments, we profiled the expression pattern of miR-137 among organs and tissues (Fig. 1D). Mir-137 was highly enriched in the brain and pituitary gland than others, however, it was also expressed in the liver, heart, kidney, muscle, intestine, stomach, white adipose tissue (WAT), and brown adipose tissue (BAT). Interestingly, miR-137 deficient mice showed severe growth retardation at postnatal day 14 with gene dosage-dependent manner (Fig. 1E). The body weight of *Mir137*^+/+^, *Mir137*^+/−^, and *Mir137*^−/−^ had gradually and significantly decreased in both male and female mice (Fig. 1F). To confirm the deficiency of miR-137, the brain, liver, and skeletal muscle were checked for expression levels (Fig. 1G). Mir-137 was undetectable in *Mir137*^−/−^. To address the consequence of severe growth retardation, we observed *Mir137*^−/−^ were dead during postnatal one month while *Mir13*^+/+^ and *Mir137*^+/−^ had no significant survival reduction (Fig. 1H). To test whether the phenomenon of postnatal early lethality was caused by intrauterine growth restriction (IUGR), we monitored newborns at postnatal day 1 (Fig. 1I). There was no significant difference in both macroscopic abnormalities and body weight between each genotype suggested the miR-137 deficiency caused growth retardation was not due to IUGR. We further evaluated the weight and the ratio to body weight of major organs in each genotype (Fig. 1J). The results showed organs including the brain, liver, heart, lung, and kidney had significant reductions in *Mir137*^−/−^ than in *Mir137*^+/+^. However, the weight ratio of organ to whole body had no significant difference among all organs suggested miR-137 deficiency caused ubiquitous growth retardation. Taken together, miR-137 may participate in body growth based on the observation from *Mir137*^−/−^.

### miR-137 deficiency conferred to abnormal bone formation, fat tissue atrophy, hypoglycemia, and hypothermia

#### Abnormal bone formation

**Fig. 2:**
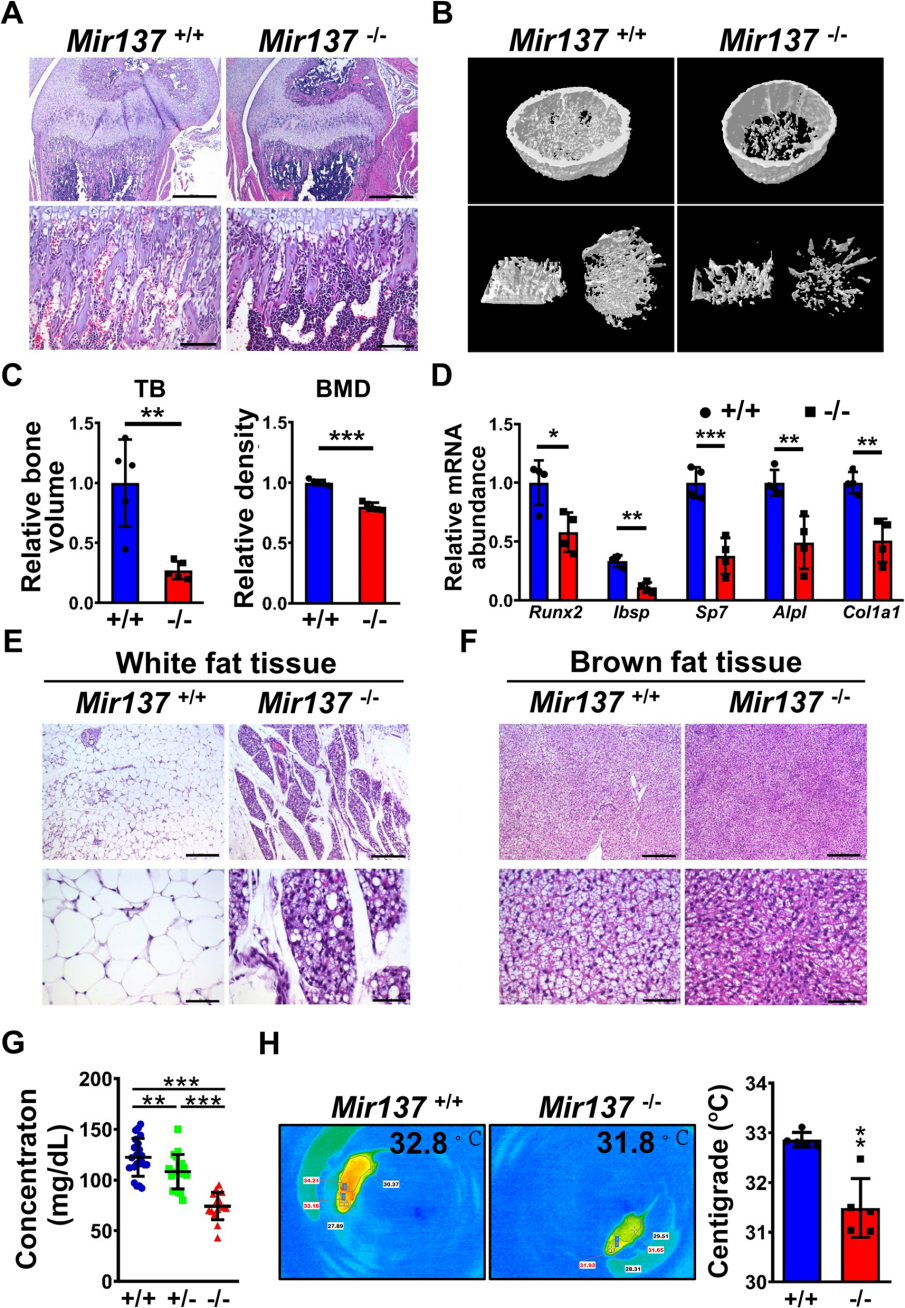
miR-137 deficiency caused defects in bone formation, fat tissues atrophy, hypoglycemia, and hypothermia. **A** Histopathological analysis of bone in miR-137 wild-type (*Mir137*^+/+^) and deficient (*Mir137*^−/−^) mice. Scale bars represented 500 (upper) and 100 (lower) μm. **B** Micro-CT analysis for trabecular bones of *Mir137*^+/+^ and *Mir137*^−/−^. **C** Quantification of trabecular bone volume and bone mineral density by micro-CT in *Mir137*^+/+^ (+/+) and *Mir137*.^−/−^ (−/−). **D** Real-time QPCR analysis of genes related to osteogenesis (*n* = 4–5). **E** Histopathological analysis of white fat tissue. Scales bars represented 200 (upper) and 50 (lower) μm. **F** Histopathological analysis of brown fat tissue. Scale bars represented 200 (upper) and 50 (lower) μm. **G** Blood glucose tests in postnatal day 14 by Accu-Check blood glucose meter (*n* = 22, 19, and 15 for +/+, +/−, and −/−, respectively). **H** Left panel, body temperature measurement by infra-red thermograph; right panel, quantification of body temperature (*n* = 5 for each group). Data was presented as the mean ± SD. *p < 0.05; **p < 0.01; ***p < 0.001 by Student’s *t* tests; N.S., non-significant. *Runx2*, Runt related transcription factor 2; *Ibsp*, Integrin binding sialoprotein; *Sp7*, Osterix; Alpl, Alkaline phosphatase; *Col1a1*, Collagen type I alpha 1

Since *Mir137*^−/−^ exhibited severe growth retardation, we further performed histopathological analysis in major organs (Additional file 1: Fig. S1). There was no macroscopic abnormality in structures and compositions. However, organs highly related to body growth such as bone and white adipose tissue showed significant defects in *Mir137*^−/−^ (Fig. 2). In bone formation, H&E staining showed *Mir137*^−/−^ had osteopenia with loss of bone mass at the distal femur condyle compared with *Mir137*^+/+^ (Fig. 2A). A micro-CT analysis was performed to evaluate the architecture and bone structural of the distal femur (Fig. 2B). *Mir137*^−/−^ showed severe osteopenia with less epiphyseal trabecular bone in the cross-sectional area. Quantification of trabecular bone (TB) volume and bone mineral density (BMD) showed both parameters were significantly reduced in *Mir137*^−/−^ than in *Mir137*^+/+^ (Fig. 2C). To further confirm whether the osteogenesis was affected due to miR-137 deficiency, we tested markers related to bone formation including runt related transcription factor 2 (*Runx2*), integrin binding sialoprotein (*Ibsp*), osterix (*Sp7*), alkaline phosphatase (*Alpl*), and type I collagen alpha 1 (*Col1a1*) in the bone tissue (Fig. 2D). All these signatures were significantly reduced in *Mir137*^−/−^ suggested miR-137 deficiency conferred to defects in bone formation.

#### Fat tissue atrophy

In addition, we also found the composition of fat body in *Mir137*^−/−^ was severely reduced (Additional file 1: Fig. S2A, left). Both inguinal WAT and inter-scapular BAT were less in *Mir137*^−/−^ and *Mir137*^+/−^ than in *Mir137*^+/+^ (Additional file 1: Fig. S2A, right). Since adipose tissue was highly related to body growth, homeostasis, and energy balance, we performed histopathological analysis in WAT and BAT (Fig. 2E and F). Compared with *Mir137*^+/+^, *Mir137*^−/−^ showed atrophy phenomena. According to the result that miR-137 was higher expressed in WAT than in BAT (Additional file 1: Fig. S2B), we tested whether loss of miR-137 induced WAT browning in atrophied fat. Transcriptional mRNA levels of markers for begging were evaluated and only *Tbx1* had significant upregulation in WAT of *Mir137*^−/−^ suggested atrophy of WAT may be not caused by hyper-browning (Additional file 1: Fig. S2C).

#### Hypoglycemia and hypothermia

The atrophy of adipose tissue may cause lack of energy and hypothermia, we further tested the blood glucose level and body temperature of mice (Fig. 2G and H). *Mir137*^−/−^ showed significant hypoglycemia under starvation condition and hypothermia compared with *Mir137*^+/+^. Taken together, miR-137 deficiency contributed to impairments of body composition and physiological energy homeostasis.

### Impairment of hepatic IGF-1 production in *Mir137*^−/−^

**Fig. 3:**
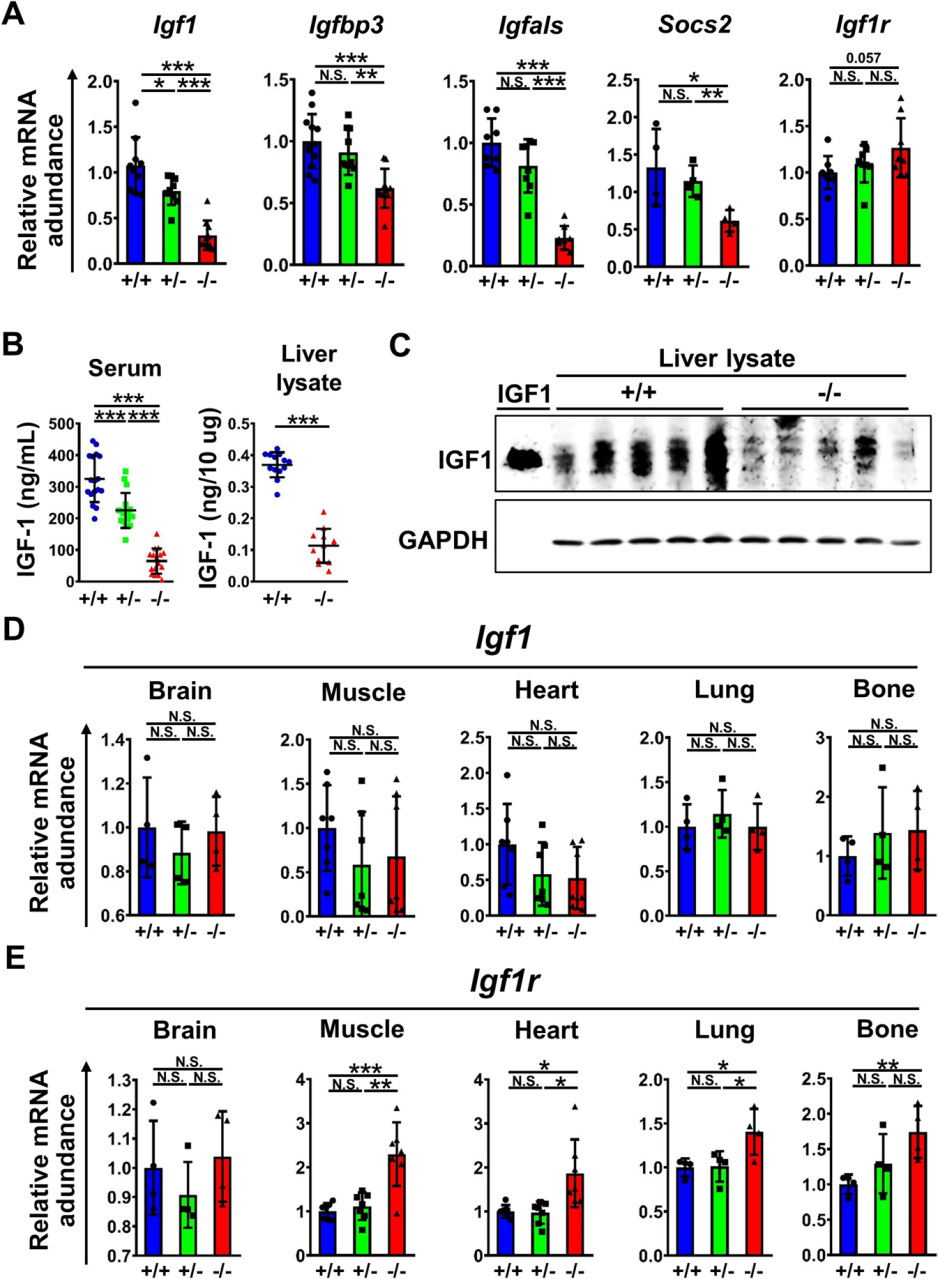
Impaired IGF-1 production and signaling in miR-137 deficient mice. **A** Expression levels of hepatic *Igf1* and *Igf1* associated genes including *Igfbp3*, *Igfals*, *Socs2*, and *Igf1r* in *Mir137*^+/+^ (+/+), *Mir137*^+/−^ (+/−), and *Mir137*^−/−^ (−/−) mice by Real-time QPCR (*n* = 8–10 for each group). **B** Serum IGF-1 (*n* = 17, 17, and 16 for +/+, +/−, and −/−, respectively) and hepatic IGF-1 (*n* = 13 for +/+ and 12 for −/−) measurements by ELISA. **C** Liver IGF-1 protein level by Western blot (*n* = 5). **D** Expression of *Igf1* mRNA levels in major organs by real-time QPCR (*n* = 4–7 for each group). **E** Expression of *Igf1r* mRNA levels in major organs by real-time QPCR (*n* = 4–7 for each group). Data was presented as the mean ± SD. *p < 0.05; **p < 0.01; ***p < 0.001 by Student’s *t* tests; N.S., non-significant. *IGF-1*, Insulin-like growth factor-1; *Igfbp3*, Insulin-like growth factor binding protein 3; *Igfals*, Insulin-like growth factor binding protein acid labile subunit; *Socs2*, Suppressor of cytokine signaling 2; *Igf1r*, Insulin-like growth factor1 receptor

IGF-1 is a critical hormone that manages the effects of growth hormone (GH) and body growth. GH/IGF-1 axis promotes normal growth of bones and tissues. The hepatocyte receives GH signal to express downstream genes including *Igf-1*, insulin-like growth factor binding protein acid labile subunit (*Igfals*), insulin-like growth factor-binding protein 3 (*Igfbp3*), and suppressor of cytokine signaling 2 (*Socs2*) [21, 22]. We tested whether the level of IGF-1 had significant changes due to miR-137 deficiency. Firstly, the transcriptional level of hepatic *Igf1* other GH downstream genes including *Igfbp3*, *Igfals*, and *Sosc2* were reduced while *Igf1r* had a slight increase (Fig. 3A). IGF-1 protein levels in circulation and liver lysate had significant reductions in *Mir137*^−/−^ confirmed by ELISA (Fig. 3B, *p* < 0.001 by Student’s *t* tests across genotypes) or Western blot (Fig. 3C). Among *Mir137*^+/+^ and *Mir137*^−/−^, circulating IGF-1 levels were also highly correlated to the body weight and genotypes (Additional file 1: Fig. S3). Although IGF-1 was majorly produced by the liver, we further tested whether IGF-1 from other organs had significant changes due to miR-137 deficiency (Fig. 3D). The result showed *Igf1* transcriptional levels in the brain, skeletal muscle, heart, lung, and bone had no significant difference between genotypes supported by hepatic IGF-1 majorly conferred to body growth. However, *Igf1r* expression in some of these organs had significant upregulation in *Mir137*^−/−^ suggested reduced IGF-1 levels leaded to compensatory upregulation in its receptor (Fig. 3E). We also tested whether the downstream signaling of organs, phosphorylation of AKT and ERK, was impacted by reduced IGF-1 (Additional file 1: Fig. S4). It indicated that despite the increased expression of *Igf1r*, these organs were still unable to compensate for the growth retardation caused by IGF-1 reduction. To directly verify whether brain miR-137 can interfere with GH-induced hepatic IGF-1 production through alternative mechanisms, without GH disruption, we conducted further tests. We also aimed to determine whether miR-137 deficiency, leading to reduced hepatic IGF-1, was indeed the primary cause of growth retardation. *Mir137*^−/−^ were administrated with exogenous upstream brain miR-137 supplementation or downstream IGF-1 treatment to validate the role of miR-137 in GH/IGF-1 signaling and IGF-1 production. MiR-137 mimic was administrated intracranially and IGF-1 intraperitoneally to mice at postnatal day 1, with subsequent monitoring of body weight and survival (Additional file 1: Fig. S5). While miR-137 alone or combined with IGF-1 partially improved weight loss and early mortality in *Mir137*^−/−^, the outcomes were not comparable to those in *Mir137*^+/+^. These results indicated miR-137 had impacts on hepatic IGF-1 production followed by impairing downstream signaling among systemic organs related to body growth.

### miR-137 regulated hepatic IGF-1 production by cell non-autonomous pathway

**Fig. 4:**
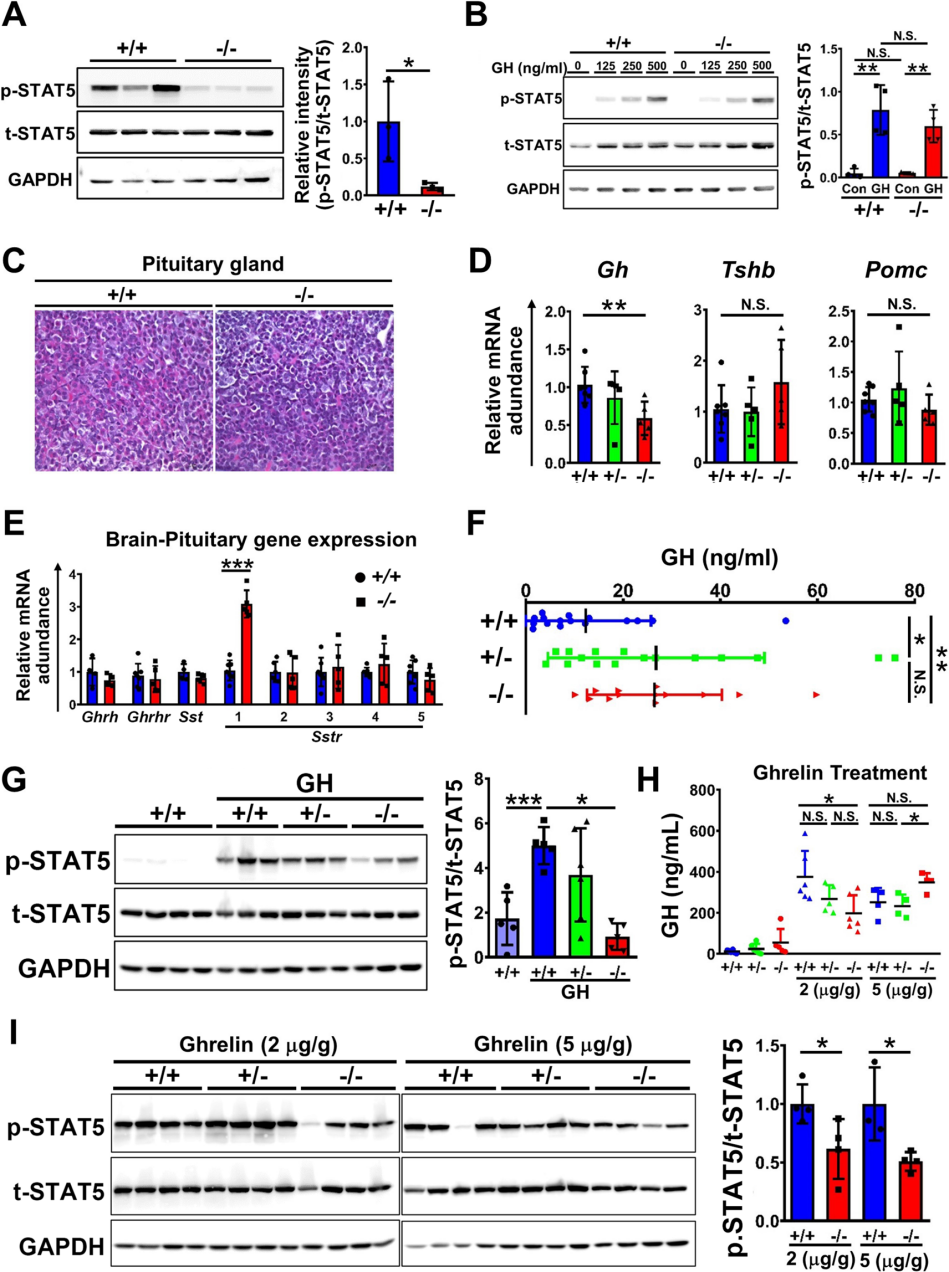
miR-137 deficiency led to growth hormone (GH) resistance through non-cell autonomous signaling. GH/IGF-1 axis signaling was tested in *Mir137*^+/+^ (+/+) and *Mir137*^−/−^ (−/−) mice for elucidating the cause of reduced IGF-1. **A** Testing of p-STAT5, a downstream mediator of GH, in the liver by Western blot (left panel). Quantification of p-STAT5 was presented as the ratio of p-STAT5/t-STAT5 (right panel) (*n* = 3). **B** Exogenous recombinant GH treatment on primary isolated hepatocytes from 14-day-old mice to test the responsiveness of GH in vitro. The p-STAT5 was measured by Western blot (left panel). Quantification of p-STAT5 was presented as the ratio of p-STAT5/t-STAT5 (right panel) (*n* = 4). **C** Histopathological analysis of pituitary gland by H&E staining. **D** Expression of hormones including *Gh, Tshb*, and *Pomc* in the mouse pituitary gland by real-time QPCR (*n* = 5–7). **E** The expression level of genes related to GH releasing regulation by real-time QPCR (*n* = 4–7). **F** Serum GH levels testing by ELISA (*n* = 16). **G** The p-STAT5 of liver in mice after GH treatment by Western blot. Postnatal day 14 mice were treated with GH (250 μg/kg) by intraperitoneal injection for 15 min followed by Western blot for liver pSTAT-5 evaluation (left panel). Quantification of p-STAT5 was presented as the ratio of p-STAT5/t-STAT5 (right panel) (*n* = 5–6). **H** Serum GH testing in mice after ghrelin stimulation by ELISA. Postnatal day 14 mice were treated with ghrelin (2–5 μg/g) by intraperitoneal injection for 15 min follow by serum GH measurement (*n* = 4–6). **i** The p-STAT5 of liver in mice by Western blot after ghrelin treatment. Livers of mice after ghrelin treatment were collected and performed Western blot for p-STAT5 evaluation (left panel). Quantification of p-STAT5 was presented as the ratio of p-STAT5/t-STAT5 (right panel) (*n* = 3–4). Data was presented as the mean ± SD. *p < 0.05; **p < 0.01; ***p < 0.001 by Student’s *t* tests; N.S., non-significant. *Gh*, Growth hormone; *Tshb*, Thyroid-stimulating hormone, beta subunit; *Pomc,* Proopiomelanocortin

Since IGF-1 production was reduced in the liver of *Mir137*^−/−^, there were two issues we should address including whether reduced hepatic IGF-1 was caused by miR-137 deficiency in the liver and whether the GH releasing from the upstream of brain had defects. Hepatic IGF-1 was synthesized by GH stimulation, growth hormone receptor (GHR) signaling was further mediated through JAK2-STAT5 pathway [23]. We found activated phosphor-STAT5 (p-STAT5) was significantly downregulated in *Mir137*^−/−^ liver compared with that in *Mir137*^+/+^ (Fig. 4A). This phenomenon was comparable to previous results that the IGF-1 level and downstream p-STAT5 were significantly reduced in the liver but no other organs (Fig. 3 and additional file 1: Fig. S6). We further tested whether the reduced p-STAT5 was caused by the absence of miR-137 in the liver by the experiment of in vitro GH treatment on isolated primary hepatocytes (Fig. 4B). Phospho-STAT5 in *Mir137*^−/−^ hepatocytes was comparable with that in *Mir137*^+/+^ hepatocytes with dosage-dependent manner after exogenous GH stimulation suggested that liver miR-137 had no significant impact on hepatic IGF-1 production. Based on this observation, we tested whether the production or secretory of GH in the pituitary gland which led to hepatic IGF-1 expression had affected in *Mir137*^−/−^. Histopathological analysis showed that eosinophilic stained GH of somatotrophs cells on H&E staining were decreased in *Mir137*^−/−^ compared with that in *Mir137*^+/+^ (Fig. 4C). In addition, we further evaluated transcriptional levels of hormones including *Gh*, *Tshb*, and *Pomc* in the pituitary grand (Fig. 4D). Only *Gh* but not *Tshb* and *Pomc* had significant reduction in *Mir137*^−/−^. Among regulators of GH production and releasing including *Ghrh* (growth hormone releasing hormone), *Ghrhr* (growth hormone releasing hormone receptor), *Sst* (somatostatin), and *Sstr* (somatostatin receptor) family, only *Sstr1* had significant upregulation in *Mir137*^−/−^ (Fig. 4E). In contrast to the pituitary gland, the serum level of GH by ELISH analysis showed a significant upregulation in *Mir137*^−/−^ suggested miR-137 deficiency may lead to GH resistance (Fig. 4F). To corroborate this hypothesis, we treated mice with over-dose GH followed by p-STAT5 evaluation in the liver (Fig. 4G). Compared with *Mir137*^+/+^ and *Mir137*^+/−^, *Mir137*^−/−^ exhibited significant reduced p-STAT5 after GH treatments. In addition to exogenous GH stimulation, we further used ghrelin which could facilitate endogenous GH releasing from the pituitary gland to test whether GH releasing was impaired in *Mir137*^−/−^ (Fig. 4H and I). After treatments with different dose of ghrelin, although secretory GH was all upregulated among all genotype mice (Fig. 4H), p-STAT5 was significantly reduced in *Mir137*^−/−^ compared with that in *Mir137*^+/+^ (Fig. 4I). The expression of *Ghr*, on major organs, was significantly decreased in the liver and brain of *Mir137*^−/−^ (Additional file 1: Fig. S7). These results suggested that growth retardation in *Mir137*^−/−^ was due to GH resistance, where GH failed to induce sufficient IGF-1 production in vivo.

### Growth retardation in *Mir137*^−/−^ was not caused by starvation and metabolic defects

**Fig. 5:**
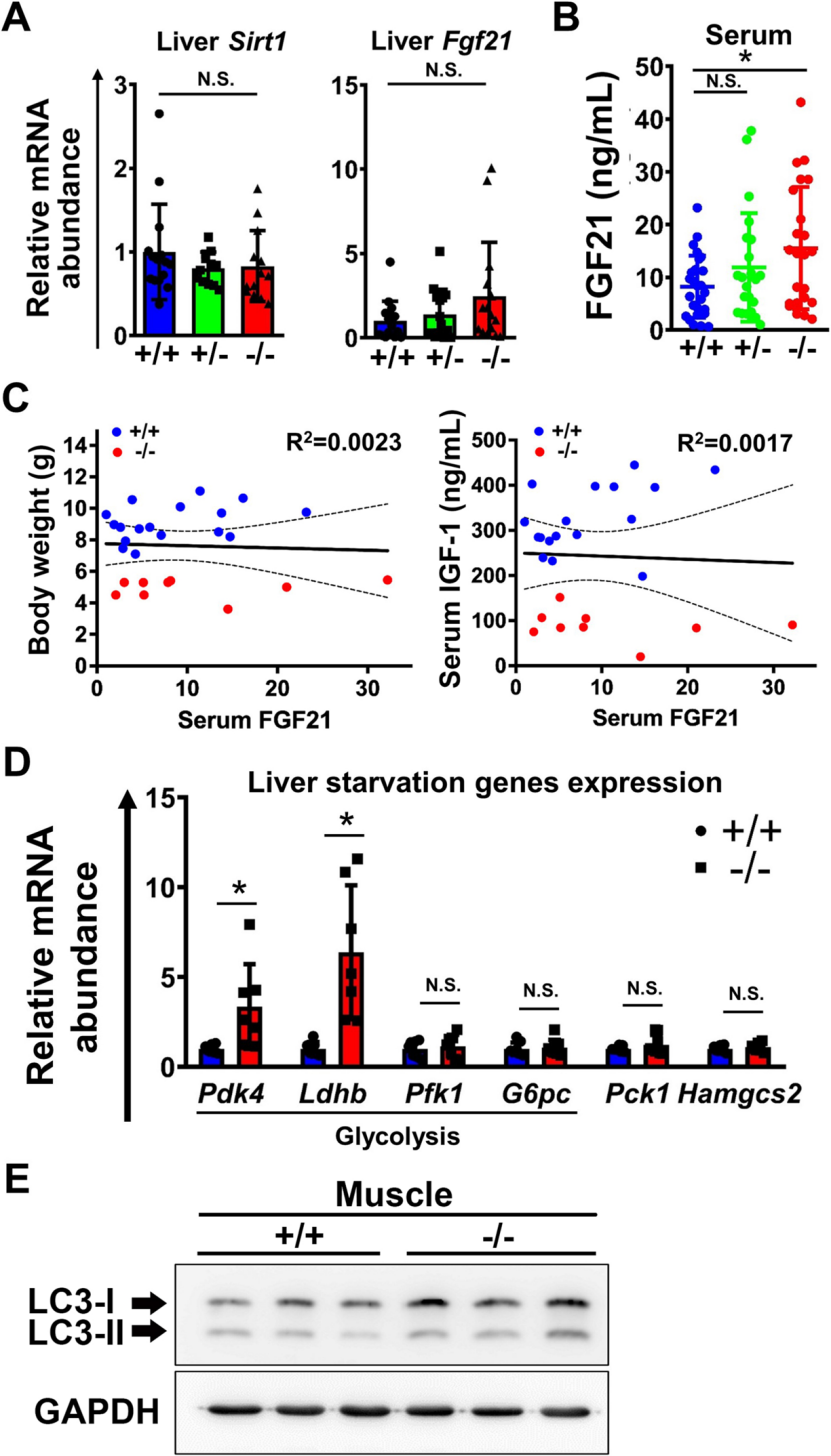
Growth retardation in miR-137 deficient mice (*Mir137*.^−/−^, −/−) was not caused by starvation or metabolic abnormalities. **A** The expression levels of *Sirt1* and *Fgf21* by real-time QPCR (*n* = 10–15). **B** Serum FGF21 measurement by ELISA (*n* = 26, 22, and 23 for +/+, +/−, and −/−, respectively). **C** Scatter diagram and correlation coefficient of body weight to serum FGF21 and serum IGF-1 to serum FGF21. **D** The expression levels of genes related to starvation or metabolism by real-time QPCR (*n* = 3–4). **E** The expression levels of autophagy signature, LC3, by Western blot (*n* = 3). miR-137 wild-type, heterozygous knockout, and homozygous knockout mice were represented as +/+, +/−, and −/−, respectively. Data were presented as the mean ± SD. *p < 0.05; **p < 0.01 by Student’s *t* tests; N.S., non-significant. *Sirt1*; AD-dependent deacetylase sirtuin-1); *Fgf21*; Fibroblast growth factor 21; *Pdk4*, Glycolysis genes: pyruvate dehydrogenase kinase 4, *Ldhb,* Lactate dehydrogenase B, *Pfk1,* Phosphofructokinases 1, *G6pc,* Glucose-6-phosphatase; *Pck1,* gene: Carboxykinase1 (Gluconeogenesis); *Hamgcs2,* 3-hydroxy-3-methylglutaryl-CoA synthase 2 (Ketogenesis gene); LC3, Microtubule-associated protein 1A/1B-light chain 3

Since malnutrition which referred to deficiencies in energy or nutrient uptake commonly caused growth retardation, we tested expression levels of hepatic *Fgf21* and *Sirt1*, key genes for *Igf1* inhibition under malnutrition condition [24, 25]. Both these two genes had no significant difference between *Mir137*^+/+^, *Mir137*^+/−^, and *Mir137*^−/−^ (Fig. 5A). The serum FGF21 levels were significantly upregulated in *Mir137*^−/−^ compared with *Mir137*^+/+^ (Fig. 5B). The correlation between serum FGF21 and body weights or serum IGF-1 was analyzed by the scatter plot (Fig. 5C). The result showed there was no significant correlation between these factors. We further tested whether metabolism was affected in *Mir137*^−/−^. Genes related to glycolysis, gluconeogenesis, and ketogenesis in the liver were tested by real-time QPCR (Fig. 5D). Only *Pdk-4* and *Ldhb* were upregulated in *Mir137*^−/−^ than in *Mir137*^+/+^. In addition, we also evaluated the formation of autophagy, a marker for mice starvation. The results showed the ratio of LC3II/LC3I had no significant difference between *Mir137*^+/+^ and *Mir137*^−/−^ (Fig. 5E). Taken together, growth retardation in miR-137 deficient mice was not the consequence of starvation.

### Brain specific miR-137 deficiency majorly caused growth retardation

**Fig. 6:**
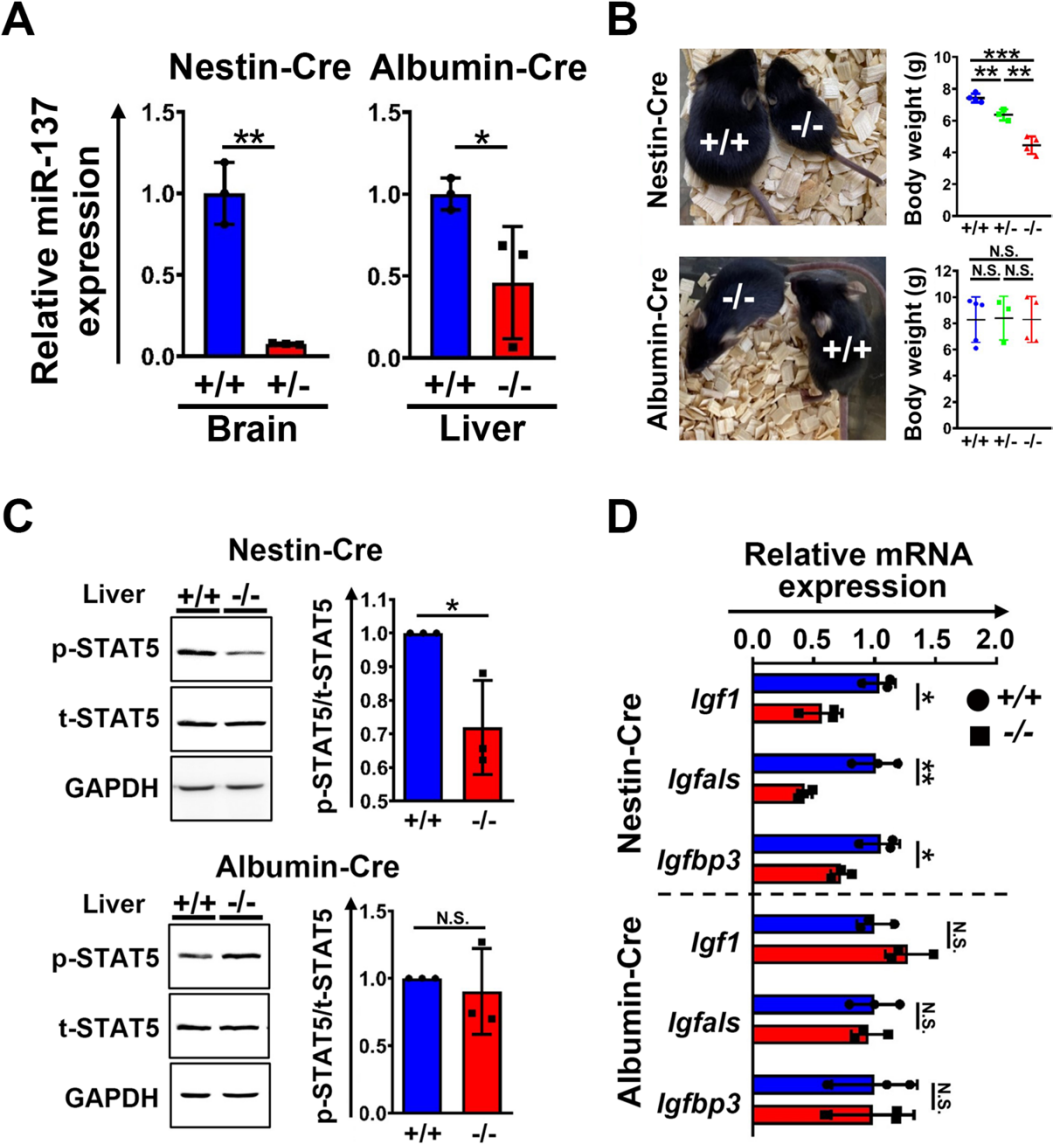
Nervous system but not liver-specific miR-137 deficiency conferred to growth retardation. **A** Expression of miR-137 in the brain and liver by Taqman QPCR (*n* = 3). **B** Photographs and body weight recording of postnatal day 14 nervous system and liver specific miR-137 deficiency mice (*n* = 3–5). **C** Expression of liver p-STAT5, a GH downstream mediator, in nervous system and liver-specific miR-137-deficient mice by Western blot. Quantification of p-STAT5 was presented as the ratio of p-STAT5/t-STAT5 (*n* = 3). **D** Expression of GH responsive genes including *Igf1*, *Igfals*, and *Igfbp3* by real-time QPCR (*n* = 3). Data were presented as the mean ± SD. *p < 0.05; **p < 0.01; ***p < 0.001 by Student’s *t* tests; N.S., non-significant. *Igf1,* Insulin-like growth factor1; *Igfbp3,* Insulin-like growth factor binding protein 3; *Igfals,* Insulin-like growth factor binding protein acid labile subunit

Although miR-137 was highly enriched in the brain, we would like to further test whether the reduction of hepatic IGF-1 and growth retardation were the consequences of miR-137 deficiency specific in the brain. In addition to elucidate this cell autonomous issue, the brain and liver (as a control) specific conditional miR-137 knockout mice were generated by crossing *lox*P-floxed in *Mir137* locus (*Mir137*^*loxP*/*loxP*^) mice with nestin-Cre or albumin-Cre transgenic mice, respectively (Additional file 1: Fig. S8). Mir-137 was significantly and obviously reduced in the brain or liver in corresponding conditional knockout mice by crossing *Mir137*^*loxP*/*loxP*^ with nestin-Cre or albumin-Cre, respectively (Fig. 6A). Mice specific deficient in brain miR-137 exhibited significant reduction in body sizes and weights similar with previous results shown in whole-body miR-137 deficiency while liver miR-137-specific deficient mice had no significant difference in body sizes and weights compared with *Mir137*^*loxP*/*loxP*^ (Fig. 6B). The histopathological analysis showed that there was no macroscopic abnormality in structures and compositions in brain-specific *Mir137*^−*/*−^ similar with whole-body *Mir137*^−*/*−^ (Additional file 1: Fig. S9). Thinner bones and fat atrophy were found in brain-specific *Mir137*^−*/*−^ (Additional file 1: Fig. S10). To confirm the consequence of brain-specific miR-137 deficiency on GH/IGF-1 signaling, the downstream signaling of GH was tested. The p-STAT5 in the liver after GH treatment in brain-specific but not liver-specific miR-137 deficient mice showed significant reductions in *Mir137*^−/−^ compared with that in *Mir137*^+/+^ (Fig. 6C). The corresponding expression levels of *Igf1*, *Igfals*, and *Igfbp3* were also tested (Fig. 6D). Results showed mRNA levels of *Igf1*, *Igfals*, and *Igfbp3* significantly decreased in brain-specific miR-137 deficient mice while these phenomena were absent in liver-specific miR-137 deficient mice. Taken together, the growth retardation in *Mir137*^−/−^ was caused majorly by brain loss of miR-137 rather than miR-137 deficiency in the liver.

### miR-137 sustains growth and development through IGF-1-mediated systemic regulatory network

**Fig. 7:**
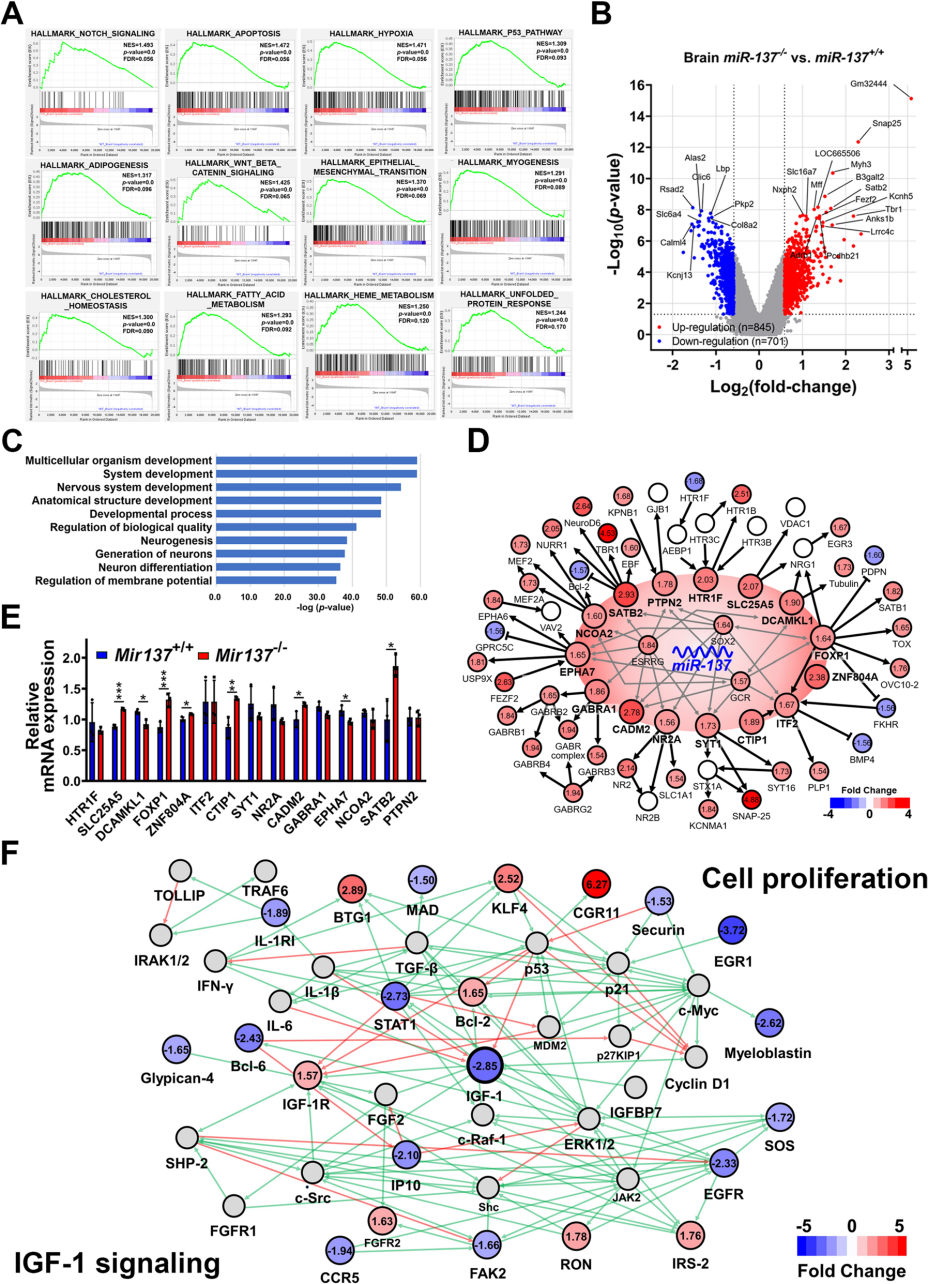
Transcriptomic and pathway analysis for miR-137 deficiency mediated growth retardation in the brain. Brain RNAs from postnatal day 14 mice of *Mir137*^+/+^ and *Mir137*^−/−^ were performed whole-genome transcriptomic analysis using expression cDNA microarray followed by enrichment and pathway analysis. **A** Positive-enriched hallmarks in *Mir137*^−/−^ brains by Gene Set Enrichment Analysis (GSEA). **B** Volcano plot analysis for differential expressed genes in brains (*Mir137*^−/−^ v.s. *Mir137*^+/+^) (*p* < 0.05 and over 1.5-fold change). **C** Gene ontology (GO) enrichment analysis of differentially genes in brains between *Mir137*^−/−^ and *Mir137*^+/+^. Enriched cellular processes with *p*-value were ranked. **D** The regulatory network constructed by potential miR-137 targets (genes around the central red oval). Red and blue colors represented upregulated and downregulated genes with the fold changes presented in numbers according to transcriptomic results. **E** Real-time QPCR validation for miR-137 potential target genes (*n* = 3). **F** Functional network connection by IGF-1. IGF-1 as a central molecule linked IGF-1 signaling to cell proliferation. IGF-1 reduction caused by miR-137 deficiency impacted several representative genes related to growth and development. Red and blue colors represent upregulated and downregulated genes with the fold changes presented in numbers according to transcriptomic results. The network was constructed based on Metacore software analysis. Data were presented as the mean ± SD. *p < 0.05; **p < 0.01; ***p < 0.001 by Student’s *t* tests; N.S., non-significant

**Fig. 8:**
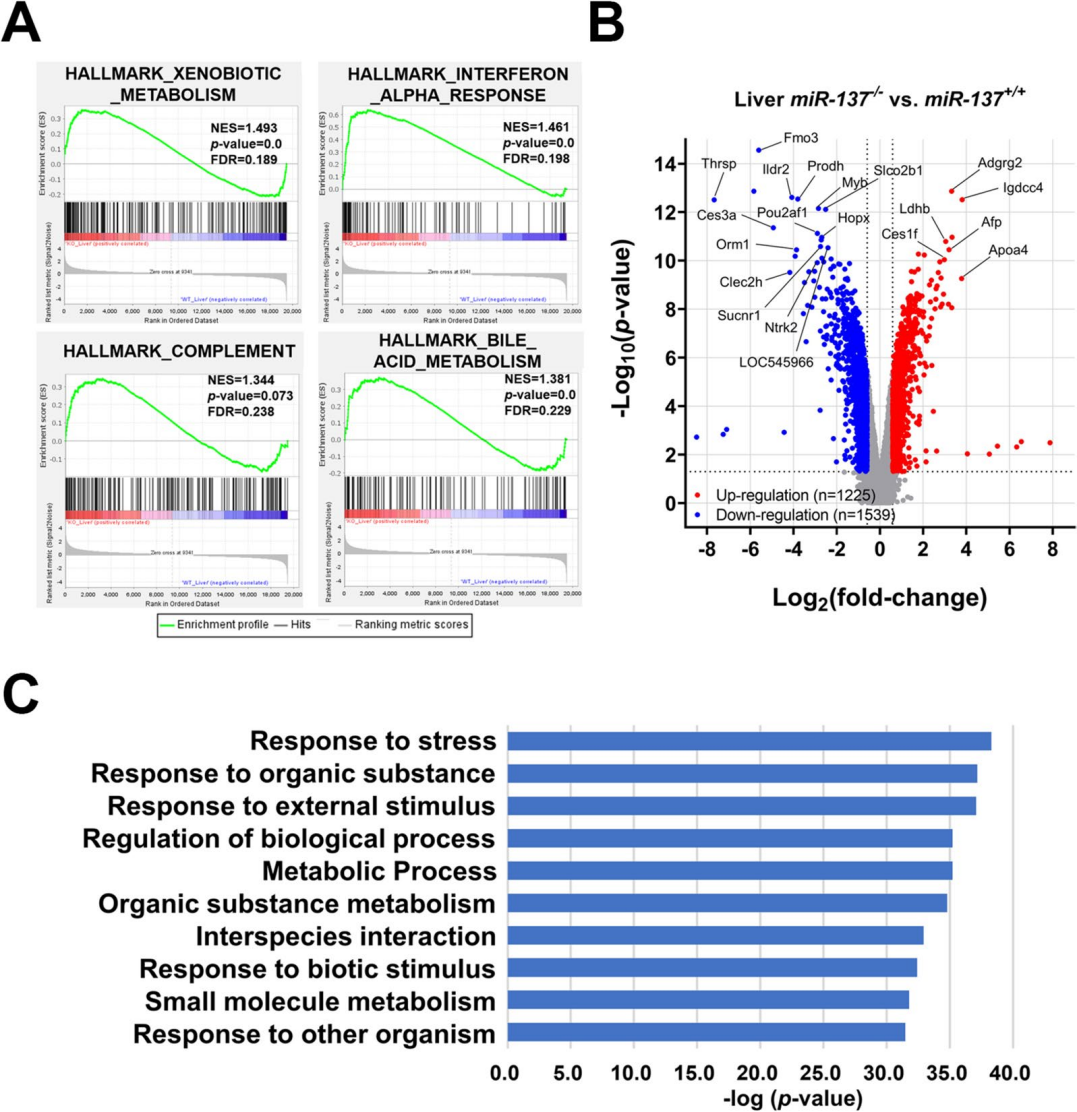
Transcriptomic and pathway analysis for miR-137 deficiency mediated growth retardation in the liver. Liver RNAs from postnatal day 14 mice of *Mir137*^+/+^ and *Mir137*^−/−^ were performed whole-genome transcriptomic analysis using expression cDNA microarray followed by enrichment and pathway analysis.** A** Positive-enriched hallmarks in *Mir137*^−/−^ livers by Gene Set Enrichment Analysis (GSEA). **B** Volcano plot analysis for differentially expressed genes in livers (*Mir137*^−/−^ v.s. *Mir137*^+/+^) (*p* < 0.05 and over 1.5-fold change). **C** Gene ontology (GO) enrichment analysis of differentially genes in livers between *Mir137*^−/−^ and *Mir137*^+/+^. Enriched cellular processes with *p*-value were ranked

Since miR-137 deficiency leaded to severe growth retardation and systemic developmental defects, we further elucidated the mechanism and molecular regulation involved in miR-137-mediated growth hormone/IGF-1 defects. RNAs from postnatal day 14 mice of *Mir137*^+/+^ and *Mir137*^−/−^ were studied through whole-genome transcriptomic analysis using expression cDNA microarray followed by enrichment and pathway analysis to identify enriched biological hallmarks and gene ontology in the brain (Fig. 7) and liver (Fig. 8) due to miR-137 deficiency. Principal component analysis (PCA) was used to illustrate the global gene expression changes among the brain and liver from *Mir137*^−/−^ and *Mir137*^+/+^ (Additional file 1: Fig. S11A). To understand the potential biological changes in the brain and liver, the complete gene expression dataset was submitted to Gene Set Enrichment Analysis (GSEA) to identify hallmark gene signatures differentially regulated by miR-137. Several core gene cluster, including Notch signaling, cellular functions, physiological metabolisms, and unfolded protein response were positive-enriched in the brain of *Mir137*^−/−^ (Fig. 7A). Other core gene clusters, including xenobiotic metabolism, IFN-α response, complement, and bile acid metabolism, were positive-enriched in the liver of *Mir137*^−/−^ (Fig. 8A). Volcano plot analysis showed that probes of genes were differentially expressed in the brain and liver between two genotypes of mice. In the brain, there were 845 and 701 genes with significant upregulation and downregulation (*p* < 0.05 and over 1.5-fold change), respectively (Fig. 7B). Similarly, there were 1225 and 1539 genes with significant upregulation and downregulation in the liver, respectively (Fig. 8B). We observed that only 208 genes were commonly found by analyses from two organs (Fig. S11B). A heatmap was created with unsupervised clustering using a total of 1546 genes from the brain and 2764 genes from the liver and showed that both genotypes were well differentiated (Additional file 1: Fig. S11C). Next, we performed enrichment analysis of the differentially expressed genes to gain insights into the cellular processes that were affected in *Mir137*^−/−^ brains and livers (Additional file 2: Table S1 and S2). Most affected gene ontology (GO) processes in the brain were development-related processes (Fig. 7C) while diverse processes including response to stress and metabolic process were enriched in the liver (Fig. 8C). To systematically figure out the miR-137 regulated network conferred to severe growth retardation, potential target genes of miR-137 with significant changes from microarray data were connected by Metacore software (Fig. 7D and Additional file 2: Table S3). The result showed a regulatory machinery was integrated under three core transcription factors including ESRRG, GCR, and SOX2. QPCR validation showed mRNA levels of SLC25A2, FOXP1, ZNF804A, CTIP1, and CADM2 were significantly upregulated (Fig. 7E). To elucidate the machinery of growth retardation caused by severely reduced IGF-1, the construction of IGF-1-centered regulatory network by combining cell proliferation and IGF-1 signaling processes based on liver transcriptomic data was performed (Fig. 7F and Additional file 2: Table S3). The result showed signaling related to cellular growth was downregulated due to reduced IGF-1 and downstream molecules. Taken together, miR-137 deficiency prompt growth retardation through systemic dysregulation.

## Discussion

GH, IGF-1, and other hormones such as sex steroids and thyroid hormone are critical for animals transitioning from maternal dependency to independent life. These hormones regulate growth and maturation through complex homeostasis mechanisms. While Cheng et al. previously reported that miR-137-deficient mice exhibited postnatal lethality due to neurodevelopment failure and disrupted Pde10a signaling, our study is the first to suggest that this phenotype may primarily result from systemic postnatal growth retardation mediated by dysregulation of the GH/IGF-1 axis [19]. The GH/IGF-1 signaling axis is well recognized as essential for individual growth and development, and various genetically engineered mouse models have been used to investigate its role [26]. GH-deficient mice exhibited abnormal body composition, reduced IGF-1 and IGFBP-3 signaling, and their body size was approximately two-thirds that of wild-type mice [27, 28]. Similarly, GHR-deficient mice (Laron mouse) exhibit severe postnatal growth retardation, proportionate dwarfism, and decreased serum IGF-1 levels [29]. Mice deficient in IGF-1 were infertile, show delayed bone development, weigh only about 30% of normal adult body weight, and have a high mortality rate [30, 31]. IGF-1R knockout mice present with fetal growth restriction and delayed epiphyseal maturation [32]. These findings underscore the critical importance of the GH/IGF-1 axis in regulating growth and development.

Despite extensive studies, how miRNAs regulate this axis remains unclear. In the current study, miR-137-deficient mice exhibited body weights of only about 30% that of wild-type controls, with significant decreased IGF-1 levels and signs of GH resistance. Moreover, *Mir137*^−/−^ died within 3 weeks after birth, a more severe phenotype than those reported in previous models, suggesting that miR-137 may act as an upstream regulator of the GH/IGF-1 axis and could be a potential therapeutic target. MiR-137 is highly expressed in the brain, and its deletion resulted in pronounced postnatal growth retardation and early mortality. Transcriptome enrichment analysis revealed that the loss of miR-137 impacted multiple growth-related pathways (Fig. 7A). One of the most significant pathways is Notch signaling, a key pathway for body growth and development. Notch signaling influences cell proliferation and maintains stem cell populations in developing organs such as the brain, liver, and muscles [33, 34]. Mechanistically, the observed growth retardation appeared to result from systemic reduced organ size reduction rather than defects from specific tissues. Transcriptomic data suggested that loss of miR-137 alters transcriptional regulatory networks in the brain. For example, SOX2 was upregulated in *Mir137*^−/−^, along with downstream targets such as ITF2, PTPN2, and EPHA7 (Fig. 7D). This aligns with previous findings indicating that miR-137 can regulate cell pluripotency through core transcriptional circuits involving SOX2 [35].

Several other genes dysregulated in *Mir137*^−/−^ further support its broad regulatory role. FOXP1, a potential target of miR-137, is involved in multiple organ development, immune regulation, and motor neuron functions [19, 36–38]. SATB2, another affected gene, is essential for skeleton development, bone regeneration, and neural differentiation [39, 40]. Additionally, miR-137 may also regulate mitochondrial energy metabolism and extracellular matrix homeostasis by targeting SLC25A5 [41]. The marked reduction of IGF-1 in *Mir137*^−/−^ likely plays a central role in linking miR-137 to cell proliferation and growth. Consistent with this, key growth-related molecules such as EGFR and SOS were also downregulated, supporting previous reports of EGF signaling’s role in postnatal somatic growth [42]. To take a close look, osteoporosis like reduced bone density, fat atrophy, hypoglycemia, and hypothermia were also found in *Mir137*^−/−^. These symptoms are consistent with known effects of GH/IGF-1 axis dysfunction, which can lead to reduced body mass, low bone density, and impaired glucose homeostasis [43]. In infants, GH or IGF-1 abnormalities are similarly linked to impaired bone growth and inflammatory imbalances [44]. Reduced circulating IGF-1 in *Mir137*^−/−^ may also indicate a shift toward energy conservation to maintain metabolic balance under stress [45]. Interestingly, although supplementation with miR-137 and IGF-1 can partially improve growth retardation in *Mir137*^−/−^, their administration in *Mir137*^+/+^ led to increased mortality. These suggested possible potential toxic effects resulting from gain-of-function, likely due to complex physiological interactions. Further investigation is warranted to optimize the application.

Mir-137 deficiency possibly led to GH resistance, as evidenced by elevated serum GH levels but reduced hepatic IGF-1 levels in *Mir137*^−/−^ [46]. In our in vivo characterization, the STAT-5 activity majorly mediated by GH/GHR was significantly attenuated in *Mir137*^−/−^ livers even after treatment with exogeneous GH or induction by Ghrelin (Fig. 4). However, in isolated primary hepatocyte from *Mir137*^−/−^, STAT-5 activity could still be induced by exogenous GH in a dose-dependent manner (Fig. 4B). This suggests that GH/GHR-mediated STAT5 activation and downstream IGF-1 expression may be disrupted in vivo, potentially due to systemic factors. To further address this possibility along the brain-liver axis, we measured p-STAT5 levels in isolated primary hepatocytes stimulated with GH and supplemented with 30% serum derived from *Mir137*^+/+^, *Mir137*^+/−^, or *Mir137*^−/−^. Notably, serum from *Mir137*^−/−^ suppressed GH-induced p-STAT5 (Additional file 1: Fig. S12A). This observation coincided with significant reduction in hepatic GHR expression in *Mir137*^−/−^ (Fig. S7). Since GHR expression is itself influenced by GH/GHR signaling [47, 48], the observed GH resistance in *Mir137*^−/−^ may be partially attributed to diminished GHR levels in the liver. Further studies, such as restoring GHR expression in *Mir137*^−/−^ livers and assessing the impact on IGF-1 production, are needed to clarify the mechanism of GH resistance in *Mir137*^−/−^. On the other hand, Wang et al. reported a novel heart-derived hormone, GDF15, can be secreted cardiomyocyte and interfere with GH signaling in the liver in pediatric heart disease [49]. However, we found no significant difference in *Gdf15* expression across major organs between *Mir137*^+/+^ and *Mir137*^−/−^ (Additional file 1: Fig. S12B), indicating that GDF15 is unlikely to contribute to GH resistance in this model.

Transcriptomic analysis of the brain identified six genes that are either predicted targets of miR-137 or encode secreted proteins. Among them, QPCR analysis revealed that 3 genes were upregulated in *Mir137*^−/−^ compared to *Mir137*^+/+^ (Additional file 2: Table S4 and Additional file 1: Fig. S12C) [50, 51]. Further functional validations are required to confirm their roles in the GH/IGF-1 signaling cascade. Importantly, brain-specific but not liver-specific miR-137-deficient mice exhibited identical growth retardation phenotype, supporting the notion that miR-137 modulates GH/IGF-1 signaling upstream in brain. Although miR-137 is expressed at much lower levels in the liver compared to the brain, liver-specific miR-137 knockout mice still showed physiological alterations related to stress response and metabolism (Fig. 8B and 8 C). These finds support the conclusion that the reduced hepatic GH receptor signaling observed in *Mir137*^−/−^ is a consequence of brain-liver axis dysfunction caused by miR-137 deficiency in the brain. We also extended the analysis on the liver transcriptome date. A regulatory network was constructed based on major GH receptor signaling cascades [52, 53] and differentially expressed genes between *Mir137*^+/+^ and *Mir137*^−/−^ livers (Additional file 1: Fig. S13A). We found that p-JAK2, a critical mediator of GH receptor signaling, was significantly decreased in *Mir137*^−/−^ livers (Additional file 1: Fig. S13B). Moreover, in the context of hypoglycemia observed in *Mir137*^−/−^, the expression level of *Slc2a4* (*Glut4*), a glucose transporter involved in carbohydrate metabolism and modulated by GH/GHR signaling [54], was also reduced (Additional file 1: Fig. 13 C). These findings are consistent with previous studies suggesting that miR-137 can alleviate oxidative stress and improve nonalcohol fatty liver disease [55, 56]. Nevertheless, the hepatic effect of miR-137 deficiency appeared more diverse and less focused compared with those in the brain, as reflected by the broader and less defined gene set enrichment patterns GSEA (Figs. 7A and 8A). Additionally, several limitations of this study should be acknowledged. First, exogenous miR-137 or IGF-1 treatment only partially rescued the growth retardation in *Mir137*^−/−^, suggesting involvement of additional complex regulations. Off-target effects from genetic manipulations, leading to inconsistencies across organ systems, should also be considered. Second, although IGF-1 levels correlated with body weight (Additional file 1: Fig. S3), dosage administration was challenging due to its broad physiological impact. It cannot determine whether exogenous IGF-1 to *Mir137*^−/−^ would allow already-retarded individual to adapt to the growth stimulation of IGF-1. Third, identifying the primary target of miR-137 remained difficult, as it may regulate a group of target genes that systematically influence the GH/IGF-1 hormonal system. These factors should be considered when interpreting the results.

Clinically, laboratory tests for growth hormone deficiency (GHD)-related growth retardation have traditionally relied on measurements of IGF-1 and IGFBP-3 levels [57, 58]. Most evidence suggests that these markers reflect endogenous GH secretion and exhibit minimal circadian variation [59]. However, there remain several limitations and clinical unmet needs. IGF-1 levels can be influenced by various factors, including age, pubertal stage, chronic illness, and malnutrition, while IGFBP-3 offers no significant advantage over IGF-1 in older children [60]. Notably, the diagnostic accuracy of IGF-1 as a screening tool for suspected GHD in children remains controversial due to inconsistent findings [61]. Although its specificity is relatively high, the low sensitivity limits its clinical utility [62]. Our recent study suggests that miR-137 is strongly correlated with IGF-1 levels and related phenotypes, indicating that it may act as a higher-order and upstream regulator in growth retardation. Furthermore, as a molecular diagnostic target, microRNAs such as miR-137 offer greater stability than plasma proteins and may provide sensitivity and accuracy in clinical testing. A prospective trial is warranted to evaluate for clinical implementation.

## Conclusions

**Fig. 9:**
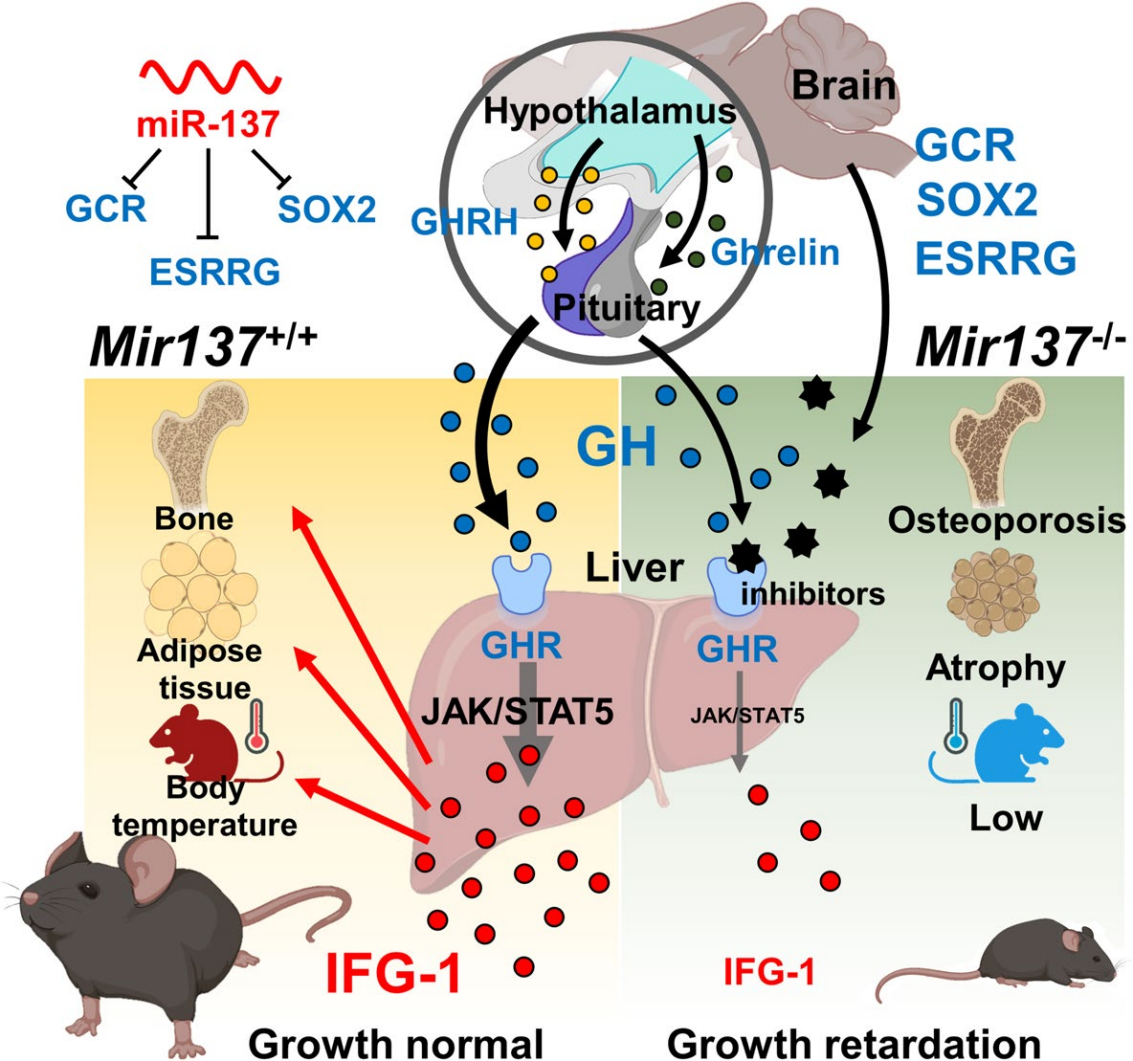
The role of miR-137 in body growth. Longitudinal growth is initiated by GH stimulated by GHRH and Ghrelin. Circulating GH can activate JAK/STAT5 signaling in the liver by binding to GHR. The major effects of GH on somatic growth are mediated by its stimulatory effects on the secretion of the IGF-1, one of the most potent activators of cell growth and proliferation, through GHR-mediated signaling. MiR-137 deficiency in the brain causes growth retardation, possibly by altering transcriptional regulatory networks involving GCR, ESRRG, and SOX2 transcription factors. This deficiency may result in either downregulated GH production or the enhancement of an uncharacterized inhibitor that impacts GHR signaling and IGF-1 production. GH, growth hormone; GHRH, growth hormone-releasing hormone; IGF-1, insulin-like growth factor-1

In this study, we revealed a potential role of miR-137 in regulating body growth and development through systemic modulation of the GH/IGF-1 axis (Fig. 9). Given the crucial role of miR-137 in hormone regulation, neural development, and contributing to the broader field of physiological functions as demonstrated in both previous studies and our current findings [15], future research may proceed in several directions. *Mir137*^−/−^ model may serve as a platform for testing novel drugs or therapeutic strategies for growth retardation. Clinically, exosome- or lipid nanoparticle-based miRNA delivery could target brain-related disorders as these systems offer the advantage of penetrating the blood–brain barrier [63, 64]. Furthermore, the additional clinical validation, blood-based detection of miR-137 could serve as a biomarker for monitoring growth and developmental status.

## Methods

### Conventional Mir137 knockout mice generation

The bMQ-453p19 bacterial artificial chromosome (BAC) clone contained mouse miR-137 exons 1–4 locus coding for predicted EST-based gene (ENSMUSESTT00000017155), from Sanger institute, UK, was used for generating targeting vectors. The targeting vector was constructed by recombineering-based strategy according to the previous study [65]. Briefly, the genomic fragment of *Mir137* with *lox*P site floxed in pL253 vector was linearized by NotI and used to replace the wild-type allele of *Mir137* in 129/sv embryonic stem (ES) cells. For generation of conventional *Mir137* knockout mice, E14 ES cells were used for transfection experiments. Growth and expansion of the ES cells, ES cell DNA isolation, and Southern blotting analysis were performed as described previously [66]. ES cells with *lox*P-floxed miR-137 (*Mir137*^*lox*P/*lox*P^) were further introduced Cre recombination to deleted a 5.5-kb region of predicted EST-based gene (spanning from exon2 and exon3 containing miR-137 transcripts). *Mir137*-deleted ES clones were identified by Southern blot analysis and injected into C57BL/6 blastocysts to establish chimeric mice. Germ-line transmission of the heterozygous *Mir137* knockout (*Mir137*^+/−^) allele was achieved by crossing the chimeric mice with wild-type C57BL/6 mice. Mice born from the intercross of the heterozygous F1 littermates were used for most of the experiments.

### Conditional nervous system and liver-specific Mir137 knockout mice generation

All mice used for conditional *Mir137* knockout mice were from the C57BL/6 genetic background. Conditional nervous system or liver-specific *Mir137* knockout mice were created by crossing *Mir137*^*lox*P/*lox*P^ with nestin-Cre or albumin-Cre transgenic mice, respectively. Nestin-Cre transgenic mice (BRC No.RBRC02412, RIKEN BioResource Research Center, JAPAN) expressed a cre recombinase gene under the control of Nestin promoter and enhancer in whole brain and spinal cord but no other major organs [67]. Albumin-Cre transgenic mice (Strain No.003574, The Jackson Laboratory, USA) from Rodent Model Resource Center of National Laboratory Animal Center, Taiwan expressed cre recombinase in the liver under the control of the mouse albumin enhancer/promoter [68]. We then crossed heterozygous mice to generate miR-137 wild-type mice (*Mir137*^*lox*P/*lox*P^) and homozygous mice (*Mir137*^*lox*P/*lox*P^; nestin-Cre). Genotype was done using tail DNA as descripted in below.

### Animal experiments

All animal experimental procedures were approved by the Institutional Animal Care and Use Committee (IACUC), National Taiwan University Medical College with IACUC number 20190231 and 20,190,234 (Investigation of the mechanism of growth retardation in miR-137 deficient mice). Mice had free access to food and water and were housed at 22 ± 1 °C with 50 ± 2% humidity and a 12 h light/12 h dark schedule. For anesthesia/euthanasia, it was based on AVMA Guidelines for the Euthanasia of Animals: 2020 Edition. Briefly, for anesthesia, we used 1.25% tribromoethanol/tert-amyl alcohol as working anesthetic by intraperitoneal (IP) injection. The dosage was 250 mg/kg for a mouse. The 100% anesthetic stock was prepared by 10 g 2,2,2-tribromoethanol (Cat. A18706, Alfa Aesar) dissolved in 10 mL tert-amyl alcohol (Cat. 240,486, Sigma Aldrich). The stock solution was stored at 4 °C and protected from light. For euthanasia, mice were deeply anesthetized with 1.25% working anesthetic (0.2 mL/10 g) followed by neck dislocation.

### Exogeneous administration of miR-137 and IGF-1

Mouse IGF-1 treatment was according to previous studies with modifications [69, 70]. The recombinant IGF-1 (Cat.250–19, PeproTech) was dissolved in 55 μl saline with 0.01% BSA while a vehicle solution was used as the control. The both solutions were stored in aliquots at − 80 °C until use, and once thawed. Mice were weighted and injected interperitoneally once every 2 days on postnatal day 1 with 25 mg/kg mouse recombinant IGF-1 or vehicle solution. Mouse neonatal free-hand intracranially miR-137 injection was based on the previous study with modifications [71, 72]. Ten micrograms Mir-137 mimic (Cat.4464066, ThermoFisher) or miRNA mimic negative control (Cat.4464059, ThermoFisher) was dissolved in 2 μl saline for injection. The both solutions were stored in aliquots at − 20 °C until use, and once thawed. For injection, neonates were collected within 12 h of birth followed by cryoanesthesia at 4 °C for 1 min. After cessation of movement, 2 μl solution was injected into the lateral ventricles (about 2 mm ventral of lambda and 2 mm depth) once on postnatal day 1 using a 10-μl Hamilton syringe (Cat.7653–01, Hamiton) with a 32-gauge needle (Cat.7803–04, Hamiton). After injection, the pups were kept on a warming pad until their color returned to normal and they started moving again. Mice body weight was recorded if a mouse from any group dead and the survival time continued to be tracked.

### Polymerase chain reaction (PCR) for genotyping

Genomic DNA was extracted from mice tails using the Wizard genomic DNA purification kit (Promega, Cat#A1120) according to the provided users’ manual. The DNA samples were mixed with PCR master mix and amplified by Veriti™ 96-Well Fast Thermal Cycler (ABI). The sequences of PCR primer used in genotyping are listed (Additional file 2: Table S5).

### Real-time quantitative PCR

Total RNA was isolated tissues or cells by TRI reagent (Sigma-Aldrich, Cat#T9424), and 2 µg of total RNA was used to synthesis of cDNA by high-capacity cDNA reverse transcription kit (ABI, Cat#4,368,814). Quantitative PCR was carried out using an ABI StepOnePlus™ Real-Time PCR System (ABI) and SYBR Green master mix (ABI, Cat#4,364,344). The relative expression of the gene was normalized to β-actin using the formula − ΔCT = − [CTgene − CTβ-actin]. The ratio of target gene mRNA to β-actin was calculated as (2^−ΔCT^). The primers used in QPCR are listed (Additional file 2: Table S6). Mature miR-137 (ABI, Cat#001129) and internal control U6 (ABI, Cat#001973) were quantified by Taqman method.

### Western blot

Total proteins from cells and tissue were extracted by lysis buffer (Thermo Fisher Scientific, M-PER Cat#78,501; T-EPR Cat#78,510) with protease and phosphatase inhibitors. Extracted proteins were sonicated and quantified by BCA assays. Proteins (30–60 µg) were separated by 8–15% SDS-PAGE and transferred to PVDF membrane (Merck Millipore). Membranes were blocked with 10% nonfat milk followed by probing with specific primary antibodies (1:1000) overnight at 4 °C. After secondary antibodies incubating for 1 h at room temperature, protein signals were visualized by chemiluminescence (Merck Millipore, Cat#WBKLS0500) and detected by FUJIFILM LAS-3000 ECL system (GE Healthcare). The protein expression level was normalized to GAPDH. Antibodies and recombinant proteins used for Western blots and animal experiments are listed: anti-GAPDH (Proteintech, Rosemont, IL, USA, Cat#60,004–1-Ig, RRID: AB_2107436, 1:10,000), anti-IGF-1 (Abcam, Cambridge, UK, Cat#ab36532, RRID: AB_733083, 1:1000), p-STAT5 (Cell Signaling Technology, Danvers, MA, USA, Cat#9359, RRID: AB_823649, 1:1000), STAT5 (Santa cruz, Dallas, TX, USA, Cat#835, RRID: AB_632446, 1:1000), AKT (Cell Signaling Technology, Cat#9272, RRID: AB_329827, 1:1000), p-AKT (Cell Signaling Technology, Cat#4060, RRID: AB_2315049, 1:1000), ERK (Cell Signaling Technology,Cat#9102, RRID: AB_330744, 1:1000), p-ERK (Cell Signaling Technology,Cat#9101, RRID: AB_331646, 1:1000),, LC3B (Cell Signaling Technology, Cat# 2775, RRID:AB_915950, 1:1000), goat anti-rabbit IgG HRP (Abcam, Cat#ab7090, RRID: AB_955417, 1:3000–5000), goat anti-mouse IgG HRP (Merck Millipore, Cat#AP124P, RRID: AB_90456, 1:3000–5000), recombinant mouse IGF-1 (PeproTech, Cranbury, NJ, USA, Cat#250–19), recombinant mouse GH (Prospec, Ness-Ziona, Israel, Cat#cyt-540), and recombinant human ghrelin (Prospec, Cat#hor-297).

### Hematoxylin and eosin (H&E) staining

Tissues were fixed in 10% formalin for 24–48 h, then embedded in paraffin after dehydration. Tissue sections were cut to a thickness of 5 µm for staining. The staining procedure was according to the routine standard protocol. Briefly, the samples were deparaffinized with xylene and rehydrated with different concentrations of ethanol before staining. The slides were then stained with hematoxylin and eosin and mounted with cover slides after washing. All experiments were carried out by the National Taiwan University College of Medicine Laboratory Animal Center.

### Blood glucose detection

Blood samples were collected by lancet submandibular blood collection from the facial vein of mice. Mice were starving over 6 h before blood collection. The blood glucose was detected by ACCU-CHEK blood glucose meter (Roche, Switzerland).

### Enzyme-linked immunosorbent assay(ELISA)

Mice blood without anticoagulant was collected and centrifuged at 3000* g* for 15 min at room temperature for serum collection. Serum hormones including IGF-1, GH, FGF21 were measured by ELISA kit including Mouse IGF-1 ELISA (R&D system, Cat#MG100), Mouse GH ELISA (Millipore, Cat#EZRMGH-45 K), and Mouse FGF21 ELISA (R&D, Cat#MF2100). All experimental procedures were followed by the operation manual.

### Trabecular bone (TB) and bone mineral density (BMD) detection by micro-tomography (micro-CT)

Postnatal 14-day-old mice were anaesthetized with isoflurane and oxygen at a flow rate of 2 L/min and scanned using a micro-CT (Bruker, SkyScan 1176). The following scanning conditions were used: trabecular bone, 9 µm resolution, 0.5 mm aluminum filter, 50 kV voltage, 140 µA current, and 3300 ms exposure; bone mineral density, 35 µm resolution, 0.5 mm aluminum filter, 50 kV voltage, 150 µA current, and 120 ms exposure. All images and imaging data were processed using software from the company. All experiments were performed by the Taiwan Mouse Clinic of Academia Sinica.

### Body temperature measure by infrared camera

Postnatal 14-day-old mice were measured by infrared camera (NEC Avio Infrared Technology, NEC F30S). Mice were put into the container to measure the temperature and camera detection distance is 34 cm. Images were acquired for illustrating. All experiments were performed by Taiwan Mouse Clinic of Academia Sinica.

### Primary hepatocyte isolation and GH stimulation

Mouse primary hepatocyte isolation was based on our previous study [73]. Briefly, a 4-week-old mouse was anesthetized and perfused at 37℃ with perfusion buffer (HBSS contained 50 mM EGTA; 1.5 ml/min for 10 min) and 0.05% type IV collagenase in HBSS (1.5 ml/min for 10 min) from the portal vein. After perfusion, the liver was excised, ground into a hepatocyte wash medium HBSS, and filtered with a 100-μm cell strainer. The filtrate was centrifuged at 50 × *g* for 2 min to collect the pellet, which was washed twice with 20 mL hepatocyte wash medium before resuspension and counting in William’s E medium containing with L-glutamine, antibiotic, 1 × insulin-transferrin-selenium-A, hydrocortisone, L-ornithine, L-lactic acid, and ethanolamine. For GH stimulation experiments, GH was added in William’s E medium for 30 min treatment.

### Transcriptomic experiments and data analysis

The transcriptomic experiments for expression profiling were performed by utilizing the GeneChip Mouse Genome 430 2.0 Array (Affymetrix, CA, USA). The procedure was based on the manufacturer’s manual. Briefly, 100 ng total RNA from individual brain or liver of postnatal day 14 male *Mir137*^+/+^ or *Mir137*^−/−^ was used for the Two-Cycle cDNA Synthesis Kit. Biotin-labelled cRNA was produced through in vitro cDNA transcription (IVT Labeling Kit, Affymetrix). After fragmentation, 20 μg cRNA was hybridized to the chip by using Affymetrix Fluidics Station 450 followed by washing and staining procedures. The final chip was scanned by GeneChip Scanner 3000 and the raw data contained in the CEL file was further analyzed with the RMA summarization algorithm and baseline to mean normalization. All original raw data had been deposited in the Gene Expression Omnibus and were available with the accession number GSE248675 (brain) and GSE248269 (liver). Significant differential gene expression was identified by statistical analysis performed with false discovery rate (FDR) correlated *p*-value < 0.05 and fold-change greater than 1.5 in each comparison. The enrichment analysis was utilized GSEA 4.0 for understanding biological knowledge (http://software.broadinstitute.org/gsea/index.jsp). The false discovery rate (FDR) is calculated by comparing the actual data with 1000 Monte-Carlo simulations. The normalized enrichment score (NES) computes the density of modified genes in the dataset with the random expectancies, normalized by the number of genes found in a given gene cluster, to consider the size of the cluster. For pathway analysis and regulatory network construction, Metacore™ (Thomson reuters) online software was utilized based on the default enrichment analysis and Transcriptional Regulation algorithm.

## Statistical analysis

For statistical analysis, *P* values were calculated using Student’s *t* test in Excel or Graphpad Prism 8. All results are presented as mean ± SD, with *n* representing the number of mice or experiment replicates. Statistical significance was determined as *P* < 0.05. *P* values are presented as follows: N.S., *P* > 0.05; *P < 0.05; **P < 0.01; ***P < 0.001.

## Supplementary Information

Additional file 1: Fig. S1-The histology of major organs in miR-137 wild-type (*Mir137*^+/+^) and miR-137 deficient (*Mir137*^−/−^). Fig. S2-Body fat composition and markers for adipose tissue testing in *Mir137*^−/−^. Fig. S3-The correlation between serum IGF-1 and body weights in mice. Fig. S4-Testing of p-AKT and p-ERK, downstream of IGF-1 signaling, by Western blot in major organs. Fig. S5-Exogenous supplements of miR-137 alone or combined with IGF-1 partially improved weight loss and early mortality in *Mir137*^−/−^. Fig. S6-Testing of p-STAT5, a downstream active molecule of GH signaling, in the liver and muscle of miR-137 wild-type (+/+) and homologous knockout (-/-) mice. Fig. S7-Transcriptional levels of *Ghr* (growth hormone receptor) in major organs of mice. Fig. S8-The breading strategy for generating nervous system and liver specific miR-137 knockout mice.Fig. S9- The histology of major organs in wild-type mice (Mir137loxP/loxP) and brain specific miR-137 deficient mice (nestin-Cre, Mir137loxP/loxP)Fig. S9- The histology of major organs in wild-type mice (Mir137loxP/loxP) and brain specific miR-137 deficient mice (nestin-Cre, Mir137loxP/loxP)Fig. S9- The histology of major organs in wild-type mice (Mir137loxP/loxP) and brain specific miR-137 deficient mice (nestin-Cre, Mir137loxP/loxP). Fig. S9- The histology of major organs in wild-type mice (*Mir137*^loxP/loxP^) and brain specific miR-137 deficient mice (nestin-Cre, *Mir137*^loxP/loxP^). Fig. S10-Histopathological analysis of bone, white adipose tissue, and brown adipose tissue in wild-type mice (*Mir137*^loxP/loxP^) and brain specific miR-137 deficient mice (nestin-Cre, *Mir137*^loxP/loxP^). Fig. S11-Transcriptomic profiling and differential expressed genes analysis of between brains and livers in the absence of miR-137. Fig. S12-Circulating factors identification for GH/GHR signaling interference in the brain-liver axis of miR-137 deficient mice. Fig. S13-Evaluation of GH receptor signaling cascades and downstream effectors in *Mir137*^−/−^ with GH resistance.

Additional file 2: Table S1-Enrichement analysis of transcriptomic changes in *Mir137*^−/−^ vs. *Mir137*^+/+^ brains using Metacore software. Table S2-Enrichement analysis of transcriptomic changes in *Mir137*^−/−^ vs. *Mir137*^+/+^ brains using Metacore software. Table S3-Network significance of process network in *Mir137*^−/−^ vs. *Mir137*^+/+^ mouse. Table S4-Gene list of up-regulated and secreted proteins^1^ in the brain of miR-137 deficient mice. Table S5-Primer sequences for genotyping. Table S6-Primer sequences for Real-time QPCR.

Additional file 3.

## Data Availability

All data generated or analyzed during this study are included in this published article, its supplementary information files, and publicly available repositories. All transcriptomic original raw data has been deposited in the Gene Expression Omnibus (https://www.ncbi.nlm.nih.gov/geo/) and were available with the accession number GSE248675 (brain) and GSE248269 (liver) [74, 75].

## Abbreviations

BAC: Bacterial artificial chromosome
BAT: Brown adipose tissue
BMD: Bone mineral density
ES cells: Embryonic stem cells
GH: Growth hormone
GHD: Growth hormone deficiency
GHR: Growth hormone receptor
GSEA: Gene set enrichment analysis
IACUC: International animal care and use committee
IGF-1: Insulin growth factor-1
IP: Intraperitoneal
IUGR: Intrauterine growth restriction
miRNAs: MicroRNAs
NES: Normalized enrichment score
TB: Trabecular bone
WAT: White adipose tissue
