# Supplementary material for: Brain miR-137 governs growth and development via GH/IGF-1 signaling
Source: BMC Biol. 2025 Jul 1;23:197. doi: 10.1186/s12915-025-02306-8 (PMC12219031; doi:10.1186/s12915-025-02306-8)
Supplement: Supplementary file 1 — Additional file 1: Fig. S1-The histology of major organs in miR-137 wild-type (Mir137+/+) and miR-137 deficient (Mir137−/−). Fig. S2-Body fat composition and markers for adipose tissue testing in Mir137−/−. Fig. S3-The correlation between serum IGF-1 and body weights in mice. Fig. S4-Testing of p-AKT and p-ERK, downstream of IGF-1 signaling, by Western blot in major organs. Fig. S5-Exogenous supplements of miR-137 alone or combined with IGF-1 partially improved weight loss and early mortality in Mir137−/−. Fig. S6-Testing of p-STAT5, a downstream active molecule of GH signaling, in the liver and muscle of miR-137 wild-type (+/+) and homologous knockout (-/-) mice. Fig. S7-Transcriptional levels of Ghr (growth hormone receptor) in major organs of mice. Fig. S8-The breading strategy for generating nervous system and liver specific miR-137 knockout mice.Fig. S9- The histology of major organs in wild-type mice (Mir137loxP/loxP) and brain specific miR-137 deficient mice (nestin-Cre, Mir137loxP/loxP)Fig. S9- The histology of major organs in wild-type mice (Mir137loxP/loxP) and brain specific miR-137 deficient mice (nestin-Cre, Mir137loxP/loxP)Fig. S9- The histology of major organs in wild-type mice (Mir137loxP/loxP) and brain specific miR-137 deficient mice (nestin-Cre, Mir137loxP/loxP). Fig. S9- The histology of major organs in wild-type mice (Mir137loxP/loxP) and brain specific miR-137 deficient mice (nestin-Cre, Mir137loxP/loxP). Fig. S10-Histopathological analysis of bone, white adipose tissue, and brown adipose tissue in wild-type mice (Mir137loxP/loxP) and brain specific miR-137 deficient mice (nestin-Cre, Mir137loxP/loxP). Fig. S11-Transcriptomic profiling and differential expressed genes analysis of between brains and livers in the absence of miR-137. Fig. S12-Circulating factors identification for GH/GHR signaling interference in the brain-liver axis of miR-137 deficient mice. Fig. S13-Evaluation of GH receptor signaling cascades and downstream effectors in Mir13 [file 12915_2025_2306_MOESM1_ESM.docx]

**Supplementary Information for**

**Brain mir-137 Sustains Body Growth and Development through GH/IGF-1 Mediated Systemic Regulatory Machinery**

Keng-Mao Liao^1^, Wei-Lun Hsu^1^, Wan-Yi Huang^1^, Wei-Jia Luo^1^, Sung-Liang Yu^1,3,4,5^, Pan-Chyr Yang^2^, Kang-Yi Su^1,3,4*^

^1^Department of Clinical Laboratory Sciences and Medical Biotechnology, College of Medicine, National Taiwan University, Taipei 100233, Taiwan

^2^Department of Internal Medicine, College of Medicine, National Taiwan University, Taipei 100233, Taiwan

^3^Centers for Genomic and Precision Medicine, National Taiwan University, Taipei 106319, Taiwan.

^4^Department of Laboratory Medicine, National Taiwan University Hospital, Taipei 100233, Taiwan

^5^Graduate Institute of Pathology, College of Medicine, National Taiwan University, Taipei 100233, Taiwan.

**Additional file 1**

**Fig. S1.** The histology of major organs in miR-137 wild-type (*Mir137*^+/+^) and miR-137 deficient (*Mir137*^-/-^).

**Fig. S2.** Body fat composition and markers for adipose tissue testing in *Mir137*^-/-^.

**Fig. S3.** The correlation between serum IGF-1 and body weights in mice.

**Fig. S4.** Testing of p-AKT and p-ERK, downstream of IGF-1 signaling, by Western blot in major organs.

**Fig. S5.** Exogenous supplements of miR-137 alone or combined with IGF-1 partially improved weight loss and early mortality in *Mir137*^-/-^.

**Fig. S6.** Testing of p-STAT5, a downstream active molecule of GH signaling, in the liver and muscle of miR-137 wild-type (+/+) and homologous knockout (-/-) mice.

**Fig. S7.** Transcriptional levels of *Ghr* (growth hormone receptor) in major organs of mice.

**Fig. S8.** The breading strategy for generating nervous system and liver specific miR-137 knockout mice.

**Fig. S9.** The histology of major organs in wild-type mice (*Mir137*^loxP/loxP^) and brain specific miR-137 deficient mice (nestin-Cre, *Mir137*^loxP/loxP^).

**Fig. S10.** Histopathological analysis of bone, white adipose tissue, and brown adipose tissue in wild-type mice (*Mir137*^loxP/loxP^) and brain specific miR-137 deficient mice (nestin-Cre, *Mir137*^loxP/loxP^).

**Fig. S11.** Transcriptomic profiling and differential expressed genes analysis of between brains and livers in the absence of miR-137.

**Fig. S12.** Circulating factors identification for GH/GHR signaling interference in the brain-liver axis of miR-137 deficient mice.

**Fig. S13.** Evaluation of GH receptor signaling cascades and downstream effectors in *Mir137*^-/-^ with GH resistance.

**Supplementary Figures**


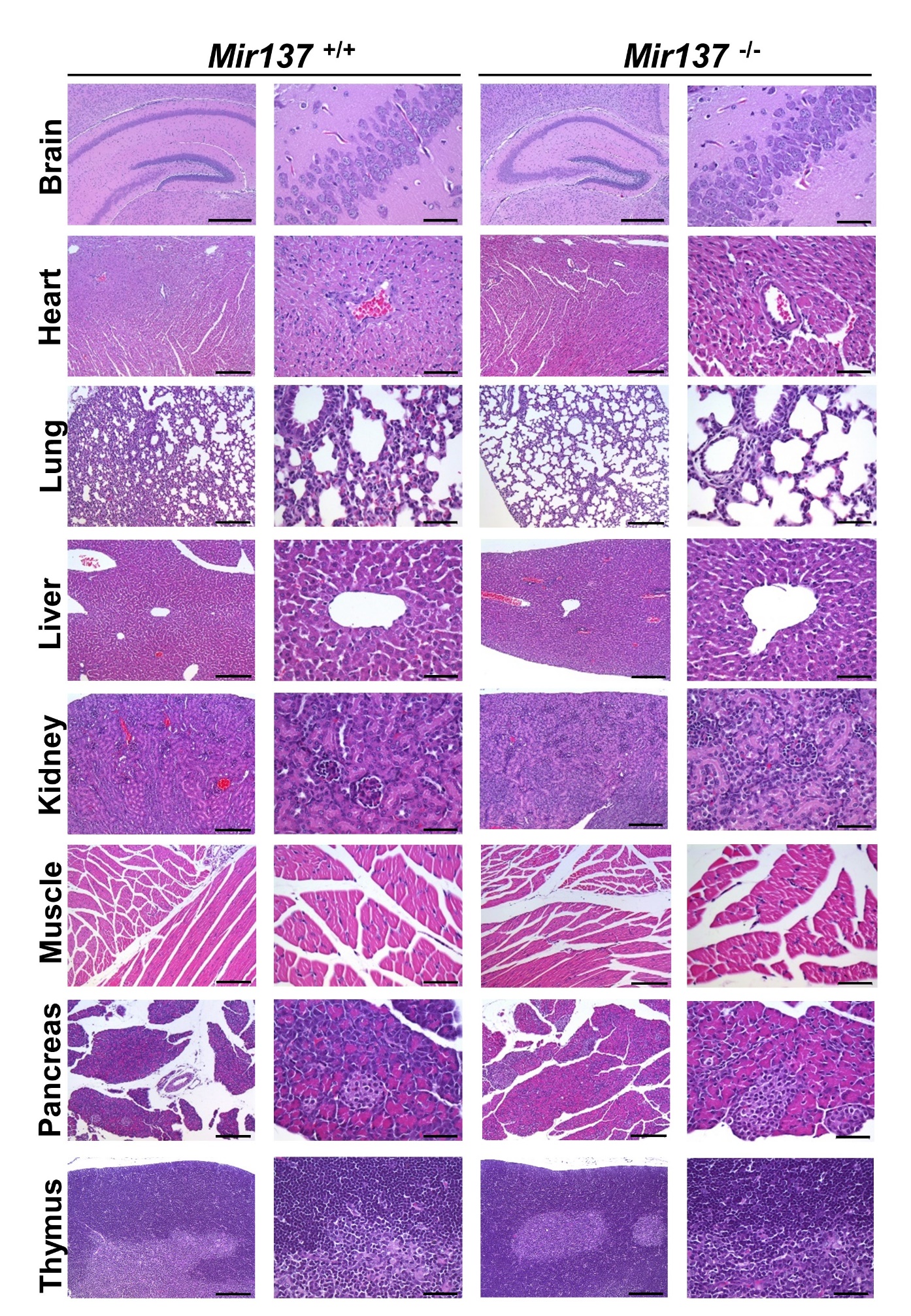


**Fig. S1.** The histology of major organs in miR-137 wild-type (*Mir137*^+/+^) and miR-137 deficient (*Mir137*^-/-^). The mice organs were isolated and embedded. Sections were performed H&E staining for histopathological analysis. Scale bars, 500 μm for the left panel of each genotype and 200 μm for the right panel of each genotype.


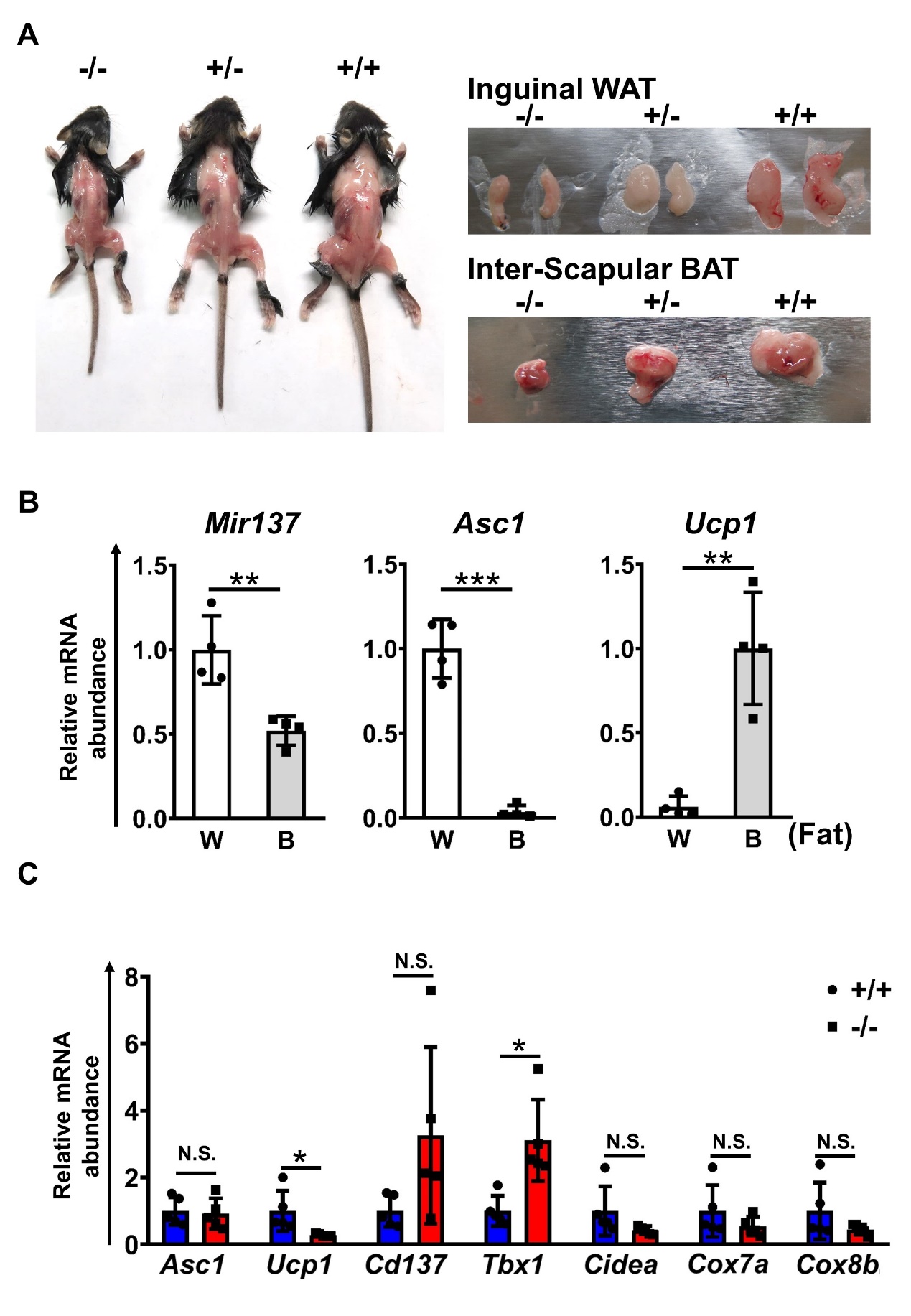


**Fig. S2.** Body fat composition and markers for adipose tissue testing in *Mir137*^-/-^. **A** Photographs of body composition in miR-137 wild-type (+/+), heterozygous knockout (+/-), and homozygous knockout (-/-) mice (left panel). Inguinal white adipose tissue (WAT) and inter-scapular brown adipose tissue (BAT) of mice (right panel). **B** Mir-137 expression in WAT (W) and BAT (B). *Asc1* and *Ucp1* are markers for WAT and BAT, respectively. **C** Marker testing for beige in WAT of +/+ and -/- mice.


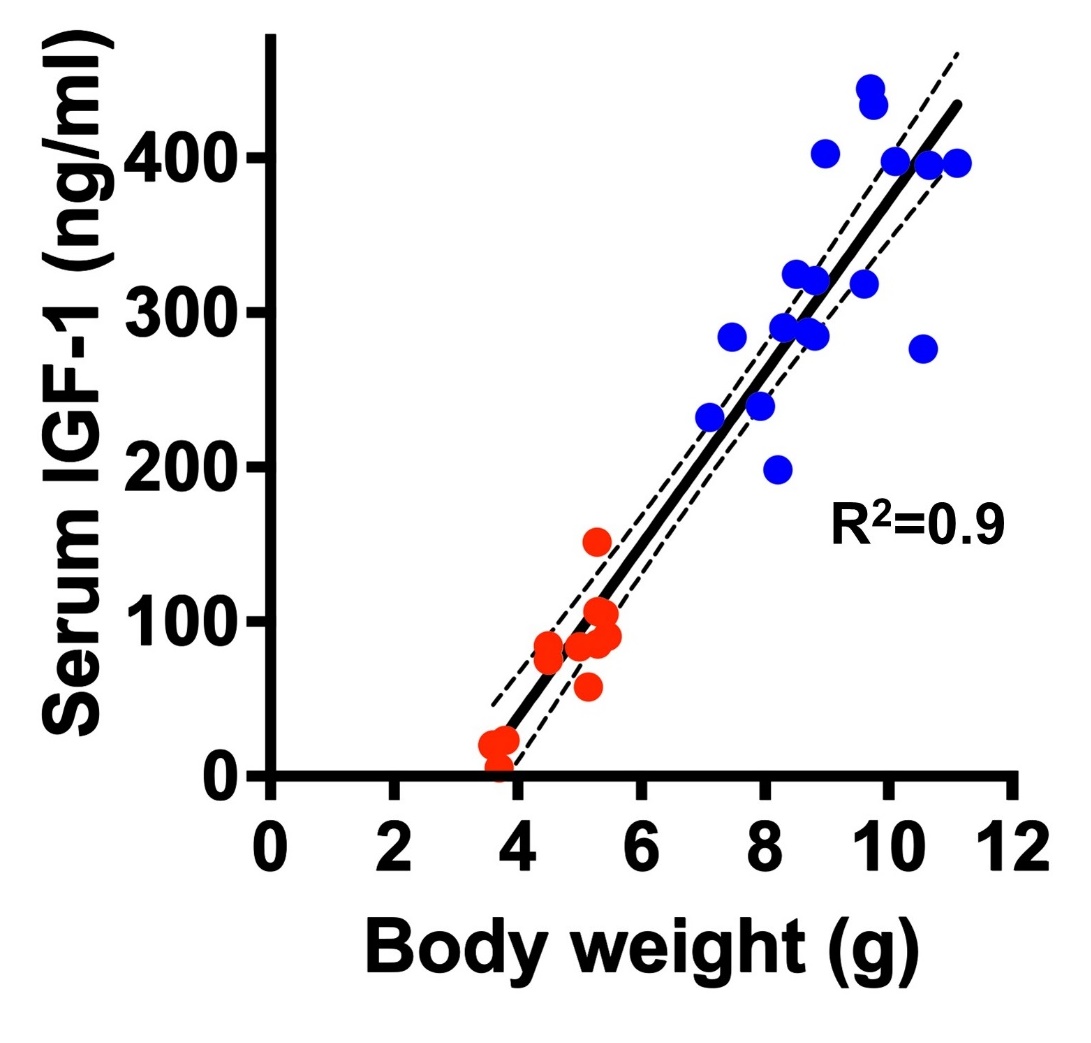


**Fig. S3.** The correlation between serum IGF-1 and body weights in mice.


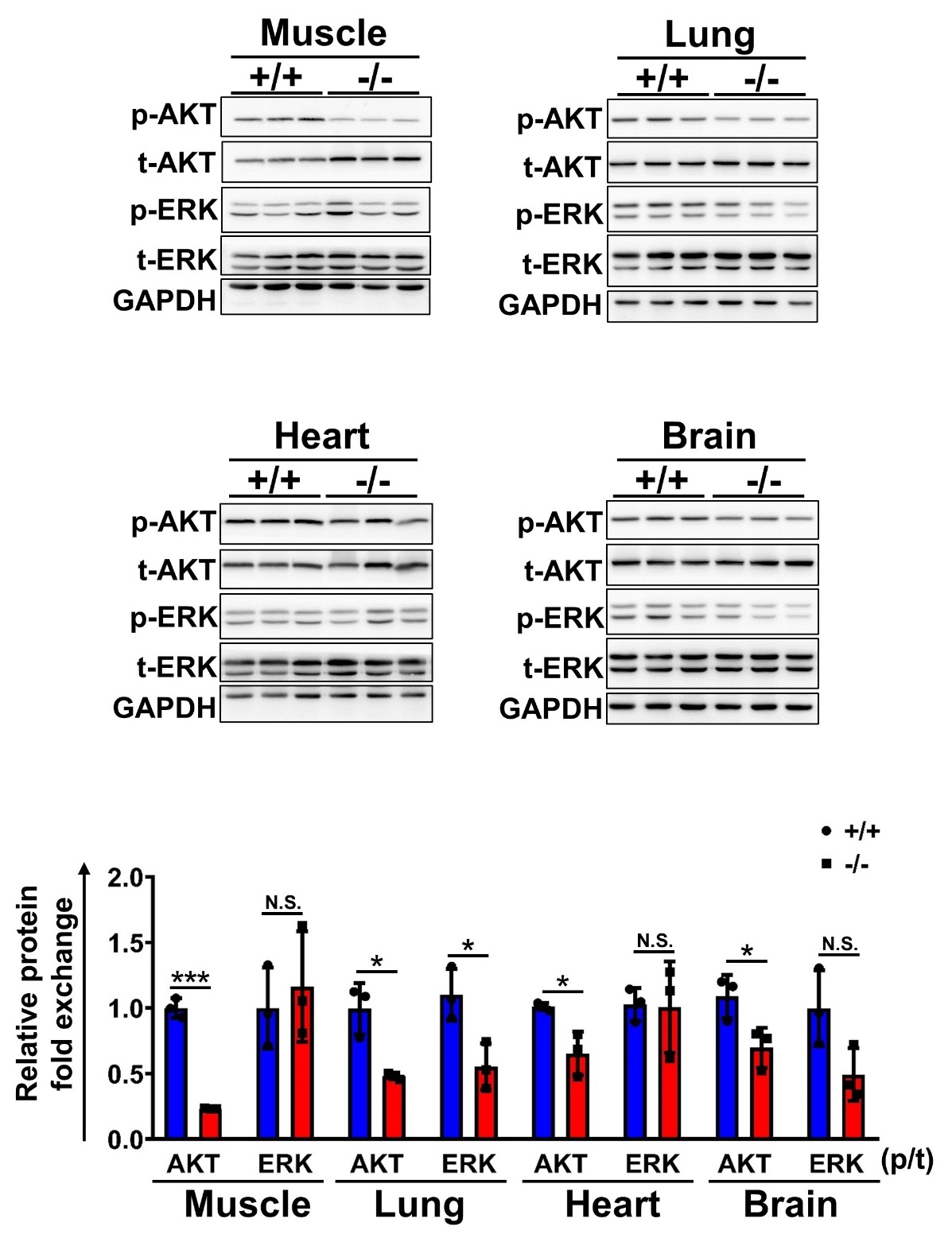


**Fig. S4.** Testing of p-AKT and p-ERK, downstream of IGF-1 signaling, by Western blot in major organs. Major organs from miR-137 wild-type (+/+) and homozygous knockout (-/-) mice were collected for Western blot (n=3 for each group) (upper panel). Quantification was performed by signal intensity related to GAPDH (lower panel). Data were presented as the mean ± SD. **p* < 0.05; ****p* < 0.001; N.S., non-significant.


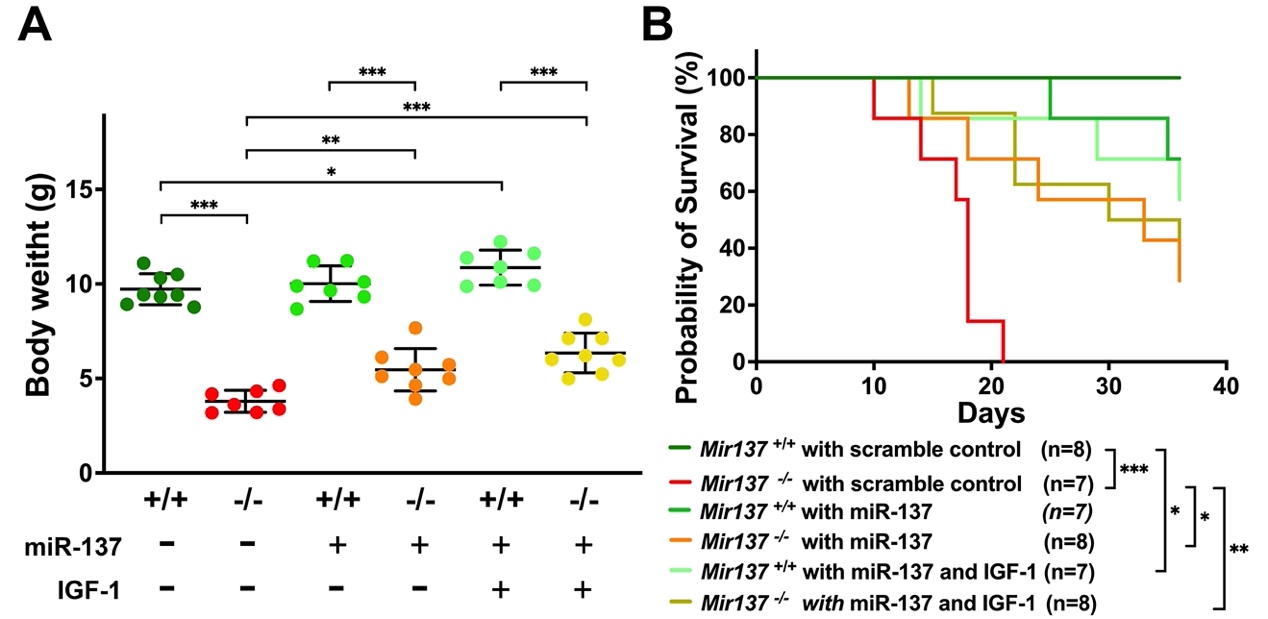


**Fig. S5.** Exogenous supplements of miR-137 alone or combined with IGF-1 partially improved weight loss and early mortality in *Mir137*^-/-^. MiR-137 was administered intracranially (10 μg miR-137 mimic in 2 μl saline) once at postnatal day 1 and IGF-1 intraperitoneally (25 mg/kg in 5 μl saline with 0.01% BSA) once every two days from postnatal day 1, with subsequent monitoring of body weight and survival. **A** Body weight recording of mice at day 10. **B** Survival curve of mice in each group from the date of birth. Data was presented as the mean ± SD. **p* < 0.05; ***p* < 0.01; ****p* < 0.001

**
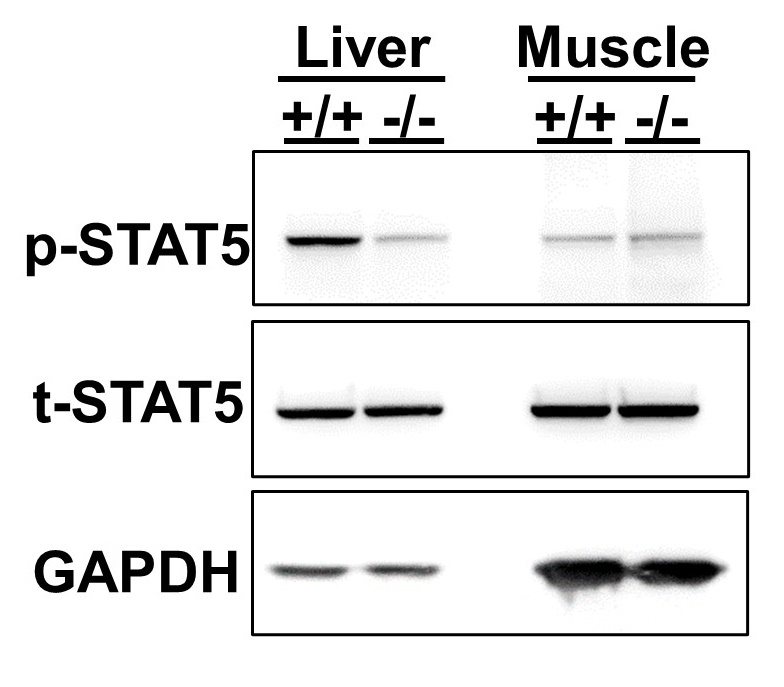
**

**Fig. S6.** Testing of p-STAT5, a downstream active molecule of GH signaling, in the liver and muscle of miR-137 wild-type (+/+) and homologous knockout (-/-) mice.


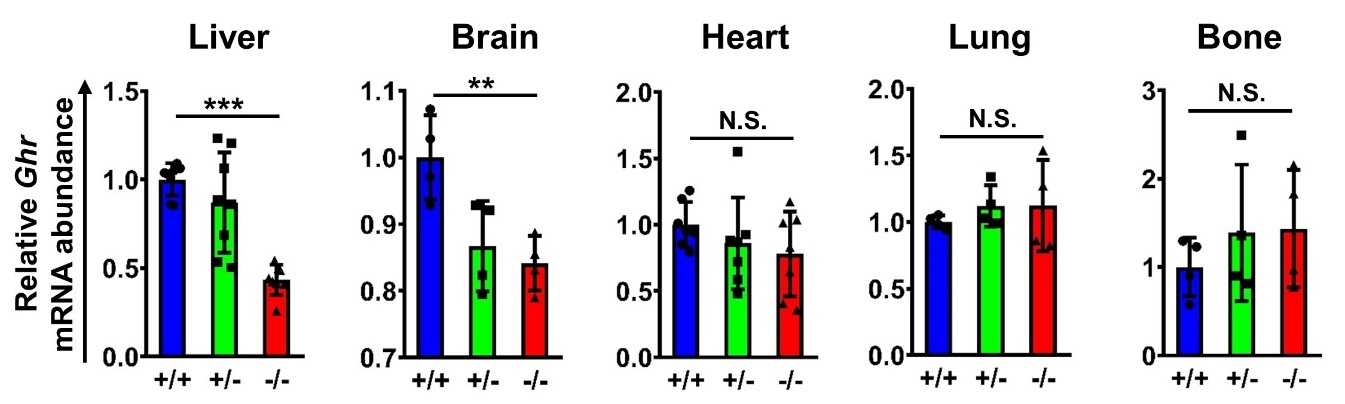


**Fig. S7.** Transcriptional levels of *Ghr* (growth hormone receptor) in major organs of mice. The relative expression of the gene was normalized to β-actin (n=3 for each group). Data were presented as the mean ± SD. ***p* < 0.01; ****p* < 0.001; N.S., non-significant. +/+, +/-, and -/- were represented miR-137 wild-type, heterozygous knockout, and homozygous knockout mice, respectively.


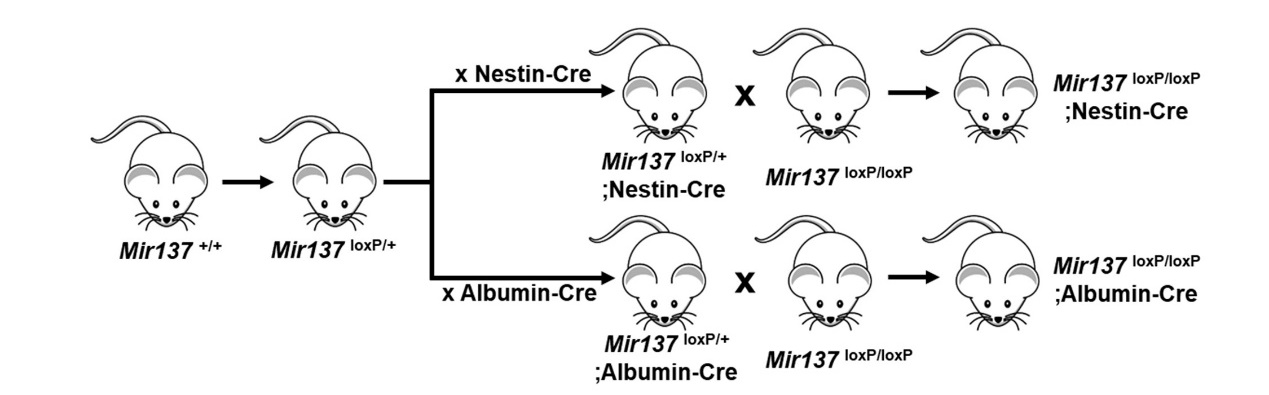


**Fig. S8.** The breading strategy for generating nervous system and liver specific miR-137 knockout mice. miR-137 wild-type mice (*Mir137*+/+) were targeted to generate heterozygous *lox*P-floxed *Mir137* locus mice (*Mir137^lox^*^P/+^). *Mir137^lox^*^P/+^ was further crossed with nestin-Cre or albumin-Cre transgenic mice to generate nervous system or liver specific miR-137 knockout mice.


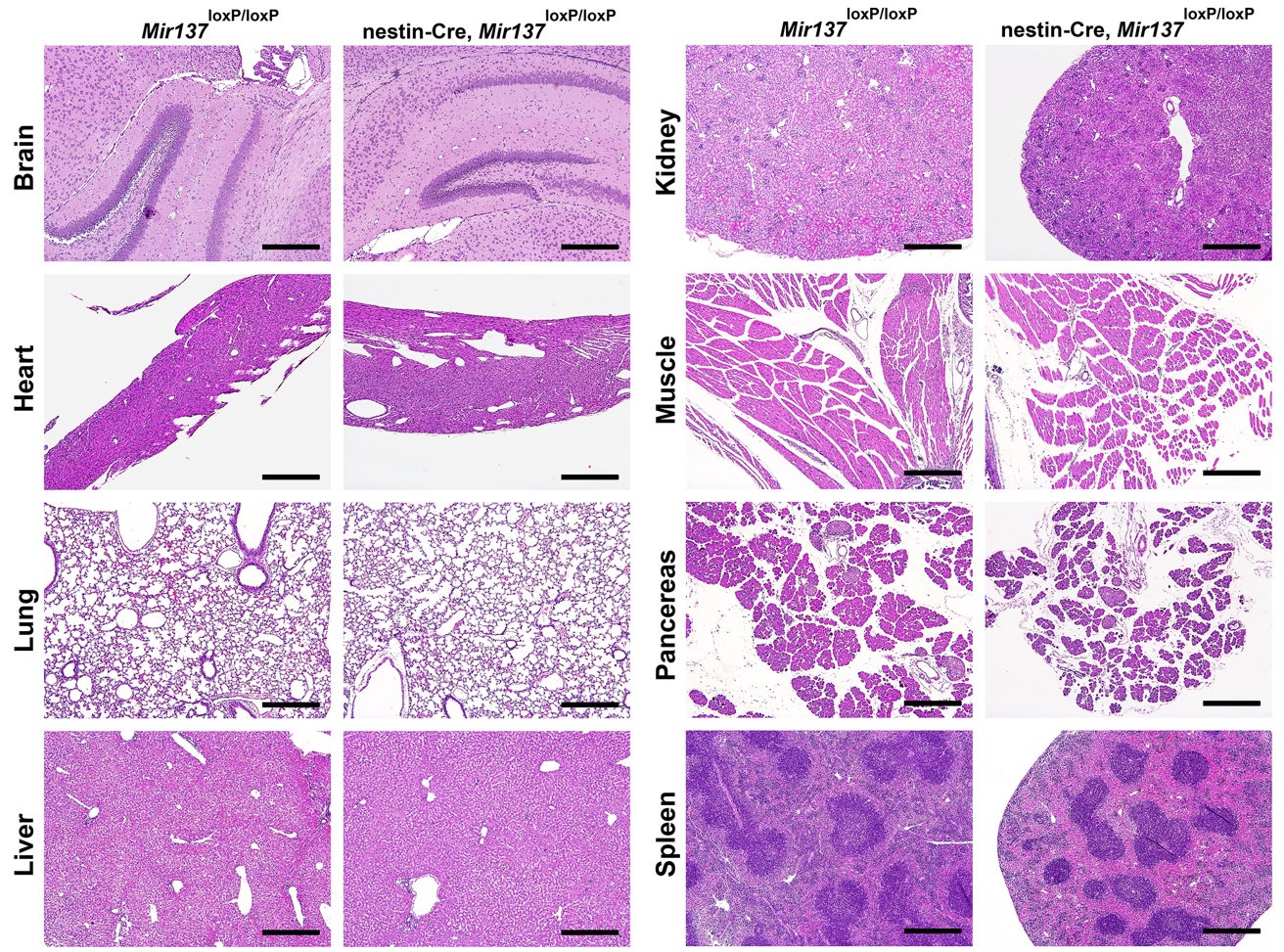


**Fig. S9.** The histology of major organs in wild-type mice (*Mir137*^loxP/loxP^) and brain specific miR-137 deficient mice (nestin-Cre, *Mir137*^loxP/loxP^). The mice organs were isolated and embedded. Sections were performed H&E staining for histopathological analysis. Scale bars, 200 μm.


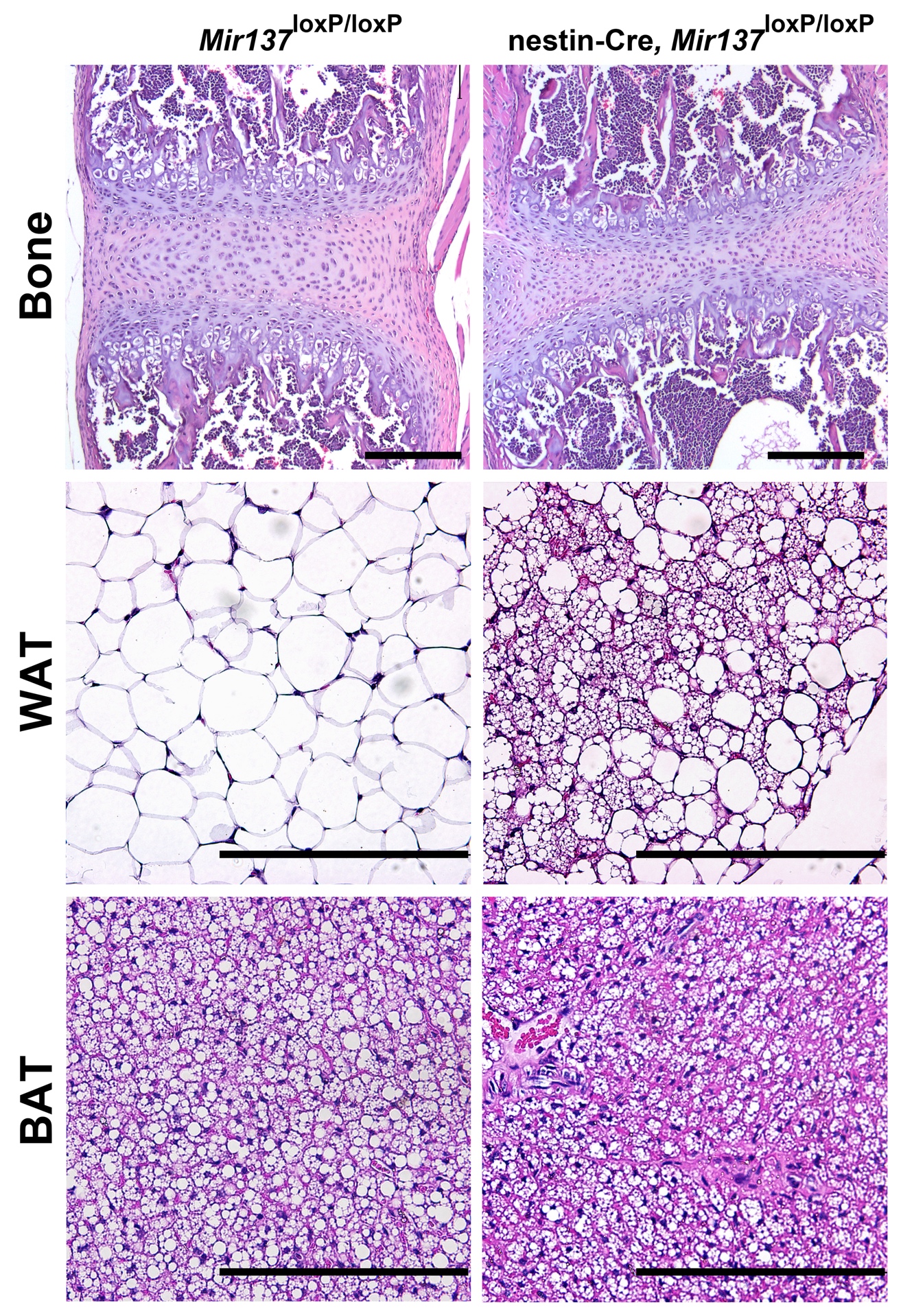


**Fig. S10.** Histopathological analysis of bone, white adipose tissue, and brown adipose tissue in wild-type mice (*Mir137*^loxP/loxP^) and brain specific miR-137 deficient mice (nestin-Cre, *Mir137*^loxP/loxP^). Scales bars represented 200 μm. WAT, white adipose tissue; BAT, brown adipose tissue.


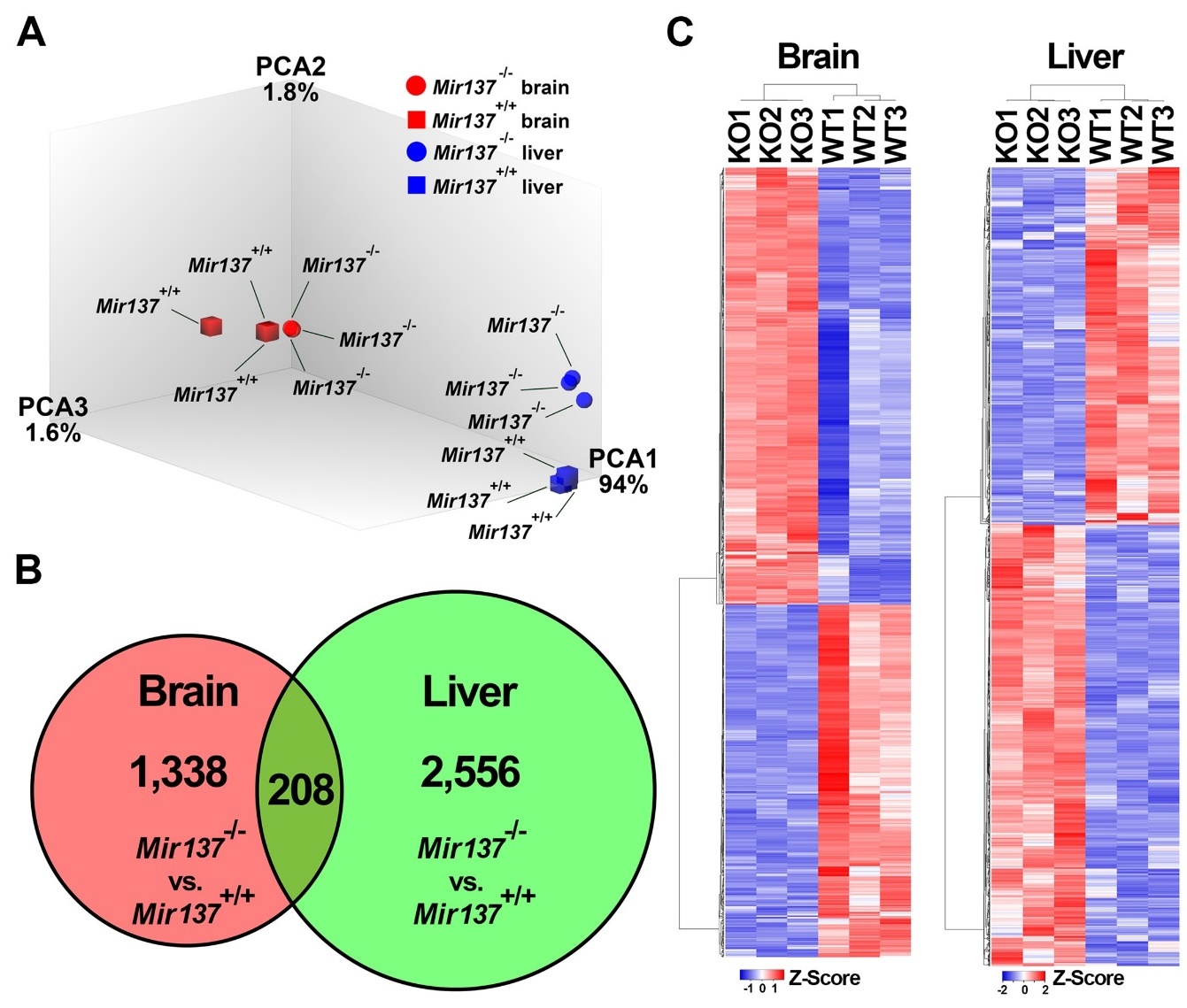


**Fig. S11.** Transcriptomic profiling and differential expressed genes analysis of between brains and livers in the absence of miR-137. RNAs from brains or livers of miR-137 wild-type (*Mir137*^+/+^, WT) and homozygous knockout (*Mir137*^-/-^, KO) mice were assessed for transcriptomic experiments followed by analysis. **A** Principal component analysis (PCA) plot for the global gene expression changes among the brain and liver from *Mir137*^+/+^ and *Mir137*^-/-^. **B** Venn diagram plot for differential expressed genes between *Mir137*^+/+^ and *Mir137*^-/-^ in the brain (left) and liver (right). **C** Unsupervised heatmap cluster analysis by differential expressed genes between *Mir137*^+/+^ and *Mir137*^-/-^ in the brain (left) and liver (right).


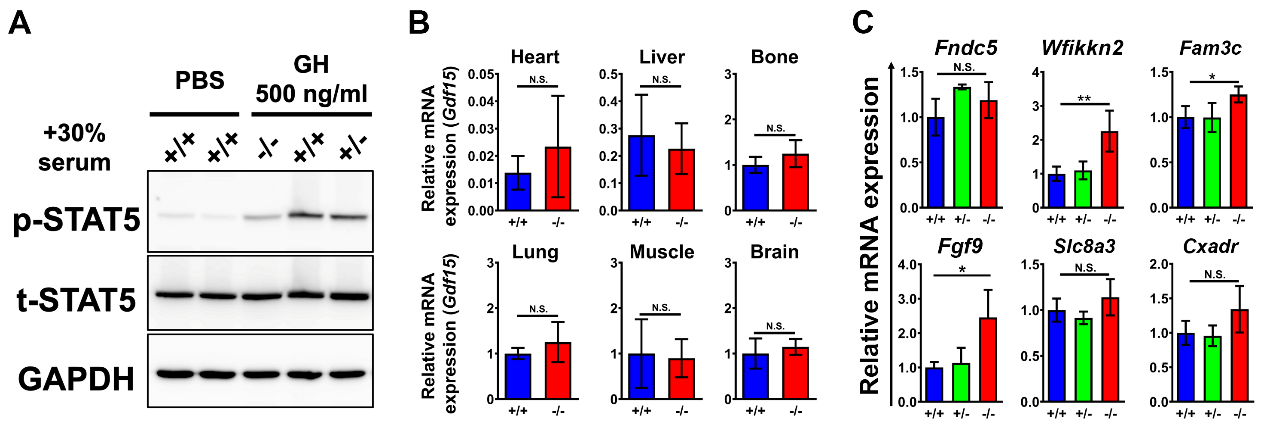


**Fig. S12.** Circulating factors identification for GH/GHR signaling interference in the brain-liver axis of miR-137 deficient mice. **A** GH/GHR downstream p-STAT5 evaluation in isolated primary hepatocytes stimulated with GH, supplemented with 30% serum from *Mir137*^+/+^, *Mir137*^+/-^, or *Mir137*^-/-^. **B** *Gdf15* expression levels by QPCR analysis in major organs of *Mir137*^+/+^ and *Mir137*^-/-^. (n=3) **C** The expression level of genes that can be secreted out of cells and may be predicted targets of miR-137 in the *Mir137*^+/+^, *Mir137*^+/-^, and *Mir137*^-/-^. (n=4) Data was presented as the mean ± SD. **p* < 0.05; ***p* < 0.01; N.S., non-significant.


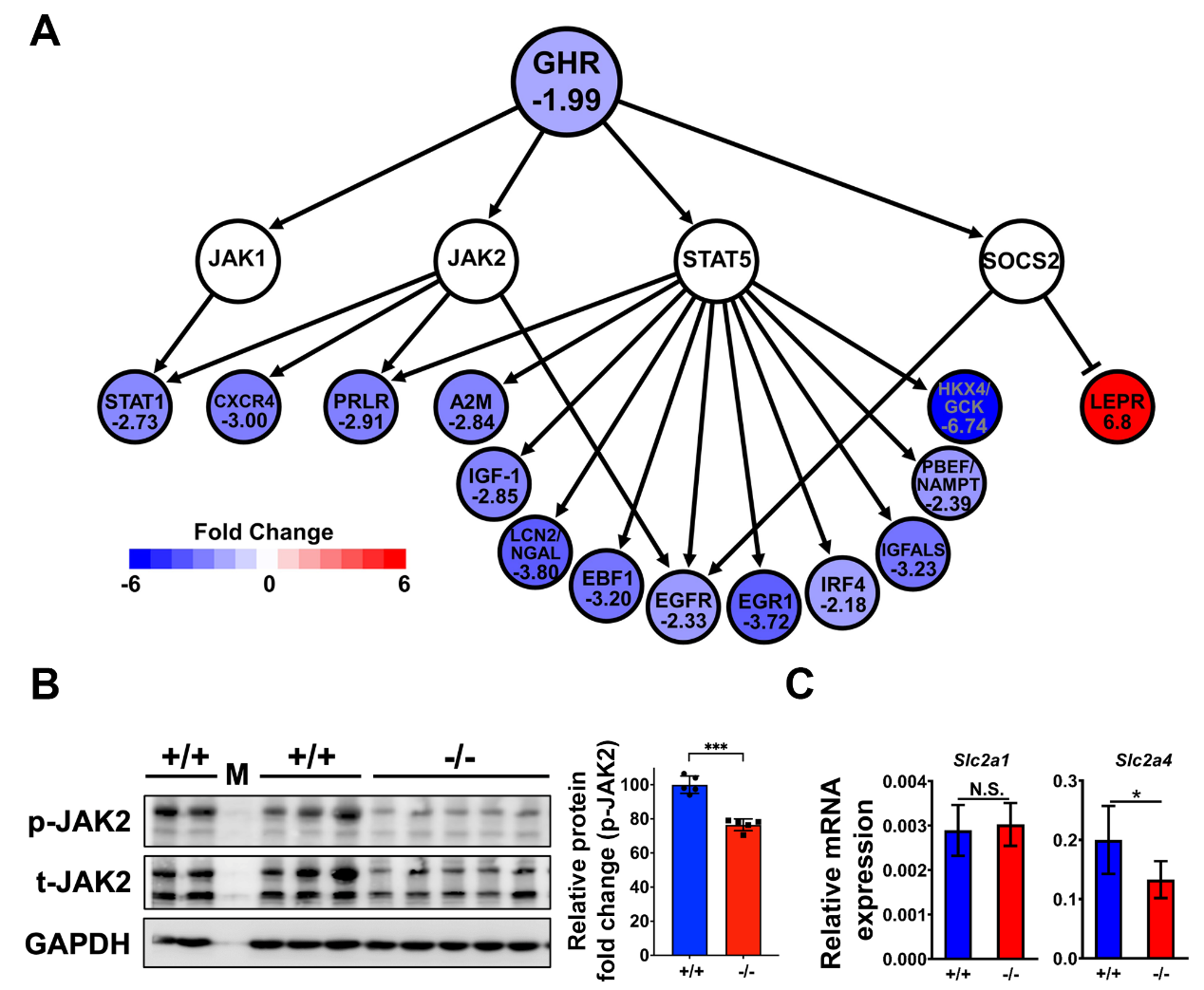


**Fig. S13.** Evaluation of GH receptor signaling cascades and downstream effectors in *Mir137*^-/-^ with GH resistance. **A** The major regulatory network of GH receptor signaling cascades and downstream effectors affected by miR-137 deficiency in the liver. Red and blue colors represented upregulated and downregulated genes with the fold changes presented in numbers according to transcriptomic results. **B** Evaluation of p-JAK in the liver of *Mir137*^+/+^ and *Mir137*^-/-^. (n=5) **C** Expression levels of major metabolism related glucose transporter genes, *Slc2a1* (*Glut1*) and *Slc2a4* (*Glut4*). (n=5) Data was presented as the mean ± SD. **p* < 0.05; N.S., non-significant.
