# Supplementary material for: Brain miR-137 governs growth and development via GH/IGF-1 signaling
Source: BMC Biol. 2025 Jul 1;23:197. doi: 10.1186/s12915-025-02306-8 (PMC12219031; doi:10.1186/s12915-025-02306-8)
Supplement: Supplementary file 2 — Additional file 2: Table S1-Enrichement analysis of transcriptomic changes in Mir137−/− vs. Mir137+/+ brains using Metacore software. Table S2-Enrichement analysis of transcriptomic changes in Mir137−/− vs. Mir137+/+ brains using Metacore software. Table S3-Network significance of process network in Mir137−/− vs. Mir137+/+ mouse. Table S4-Gene list of up-regulated and secreted proteins1 in the brain of miR-137 deficient mice. Table S5-Primer sequences for genotyping. Table S6-Primer sequences for Real-time QPCR. [file 12915_2025_2306_MOESM2_ESM.docx]

**Supplementary Information for**

**Brain mir-137 Sustains Body Growth and Development through GH/IGF-1 Mediated Systemic Regulatory Machinery**

Keng-Mao Liao^1^, Wei-Lun Hsu^1^, Wan-Yi Huang^1^, Wei-Jia Luo^1^, Sung-Liang Yu^1,3,4,5^, Pan-Chyr Yang^2^, Kang-Yi Su^1,3,4*^

^1^Department of Clinical Laboratory Sciences and Medical Biotechnology, College of Medicine, National Taiwan University, Taipei 100233, Taiwan

^2^Department of Internal Medicine, College of Medicine, National Taiwan University, Taipei 100233, Taiwan

^3^Centers for Genomic and Precision Medicine, National Taiwan University, Taipei 106319, Taiwan.

^4^Department of Laboratory Medicine, National Taiwan University Hospital, Taipei 100233, Taiwan

^5^Graduate Institute of Pathology, College of Medicine, National Taiwan University, Taipei 100233, Taiwan.

**Additional file 2**

**Table S1.** Enrichement analysis of transcriptomic changes in *Mir137*^-/-^ vs. *Mir137*^+/+^ brains using Metacore software.

**Table S2.** Enrichement analysis of transcriptomic changes in *Mir137*^-/-^ vs. *Mir137*^+/+^ brains using Metacore software.

**Table S3.** Network significance of process network in *Mir137*^-/-^ vs. *Mir137*^+/+^ mouse

**Table S4.** Gene list of up-regulated and secreted proteins^1^ in the brain of miR-137 deficient mice.

**Table S5.** Primer sequences for genotyping.

**Table S6.** Primer sequences for Real-time QPCR.

| **Table S1. Enrichment analysis of transcriptomic changes in *Mir137*-/- vs. *Mir137*+/+ brains using Metacore software.** | | | | | | |
| --- | --- | --- | --- | --- | --- | --- |
| Ranking | Maps | Total genes | p-value | FDR adjusted p-value | Genes in data |  |
| ***Enrichment by Pathway Maps*** | | | | | | |
| 1 | Neurophysiological process_Regulation of intrinsic membrane properties and excitability of cortical pyramidal neurons | 74 | 1.05E-07 | 8.32E-05 | 15 |  |
| 2 | Neurophysiological process_Constitutive and regulated NMDA receptor trafficking | 65 | 1.25E-07 | 8.32E-05 | 14 |  |
| 3 | Neurophysiological process_Dynein-dynactin motor complex in axonal transport in neurons | 54 | 7.00E-07 | 3.10E-04 | 12 |  |
| 4 | Protein folding and maturation_Posttranslational processing of neuroendocrine peptides | 50 | 2.27E-06 | 7.55E-04 | 11 |  |
| 5 | Altered Ca2+ handling in heart failure | 35 | 4.83E-06 | 1.14E-03 | 9 |  |
| 6 | Neurophysiological process_GABA-A receptor life cycle | 27 | 5.16E-06 | 1.14E-03 | 8 |  |
| 7 | Neurophysiological process_Glutamic acid regulation of Dopamine D1A receptor signaling | 45 | 5.98E-06 | 1.14E-03 | 10 |  |
| 8 | Neurophysiological process_Leptin signaling in neurons | 50 | 1.62E-05 | 2.69E-03 | 10 |  |
| 9 | Neuroprotective action of lithium | 63 | 2.39E-05 | 3.06E-03 | 11 |  |
| 10 | Signal transduction_Beta-adrenergic receptors signaling via Cyclic AMP | 42 | 2.40E-05 | 3.06E-03 | 9 |  |
| ***Enrichment by GO Processes*** | | | | | | |
| 1 | multicellular organism development | 6440 | 9.33E-60 | 4.63E-56 | 518 |  |
| 2 | system development | 5418 | 1.03E-59 | 4.63E-56 | 464 |  |
| 3 | nervous system development | 3412 | 5.21E-55 | 1.56E-51 | 339 |  |
| 4 | anatomical structure development | 8077 | 3.18E-49 | 7.11E-46 | 574 |  |
| 5 | developmental process | 8656 | 3.97E-49 | 7.11E-46 | 601 |  |
| 6 | regulation of biological quality | 4388 | 6.07E-42 | 9.08E-39 | 367 |  |
| 7 | neurogenesis | 2105 | 3.56E-39 | 4.56E-36 | 224 |  |
| 8 | generation of neurons | 1841 | 1.62E-38 | 1.81E-35 | 205 |  |
| 9 | neuron differentiation | 1746 | 3.12E-37 | 3.11E-34 | 196 |  |
| 10 | regulation of membrane potential | 743 | 4.80E-36 | 4.30E-33 | 118 |  |
| ***Enrichment by Process Networks*** | | | | | | |
| 1 | Neurophysiological process_Transmission of nerve impulse | 213 | 5.51E-10 | 8.53E-08 | 39 |  |
| 2 | Cell adhesion_Synaptic contact | 176 | 1.00E-06 | 7.75E-05 | 29 |  |
| 3 | Development_Neurogenesis_Synaptogenesis | 179 | 1.31E-05 | 6.77E-04 | 27 |  |
| 4 | Reproduction_Progesterone signaling | 216 | 5.93E-05 | 2.25E-03 | 29 |  |
| 5 | Cytoskeleton_Cytoplasmic microtubules | 115 | 7.26E-05 | 2.25E-03 | 19 |  |
| 6 | Cytoskeleton_Intermediate filaments | 81 | 1.11E-04 | 2.88E-03 | 15 |  |
| 7 | Reproduction_GnRH signaling pathway | 166 | 2.15E-04 | 4.32E-03 | 23 |  |
| 8 | Reproduction_Gonadotropin regulation | 199 | 2.23E-04 | 4.32E-03 | 26 |  |
| 9 | Neurophysiological process_GABAergic neurotransmission | 140 | 9.68E-04 | 1.67E-02 | 19 |  |
| 10 | Development_Neurogenesis in general | 193 | 1.82E-03 | 2.82E-02 | 23 |  |
| ***Enrichment by Disease (by Biomarkers)*** | | | | | | |
| 1 | Thoracic Neoplasms | 20431 | 5.70E-43 | 1.59E-39 | 940 |  |
| 2 | Lung Neoplasms | 20403 | 2.11E-42 | 2.94E-39 | 938 |  |
| 3 | Genital Diseases | 18794 | 3.32E-42 | 3.08E-39 | 890 |  |
| 4 | Respiratory Tract Diseases | 21469 | 4.64E-42 | 3.23E-39 | 967 |  |
| 5 | Respiratory Tract Neoplasms | 20512 | 7.02E-42 | 3.91E-39 | 940 |  |
| 6 | Rectal Neoplasms | 7943 | 2.38E-41 | 1.11E-38 | 494 |  |
| 7 | Lung Diseases | 21289 | 4.62E-40 | 1.84E-37 | 958 |  |
| 8 | Genital Neoplasms, Female | 15501 | 1.10E-39 | 3.83E-37 | 777 |  |
| 9 | Skin and Connective Tissue Diseases | 15093 | 2.53E-39 | 7.83E-37 | 762 |  |
| 10 | Genital Diseases, Female | 15681 | 6.88E-39 | 1.91E-36 | 781 |  |

| **Table S2. Enrichment analysis of transcriptomic changes in *Mir137*-/- vs. *Mir137*+/+ brains using Metacore software.** | | | | | |
| --- | --- | --- | --- | --- | --- |
| Ranking | Maps | Total genes | p-value | FDR adjusted p-value | Genes in data |
| ***Enrichment by Pathway Maps*** | | | | | |
| 1 | Protein folding and maturation_Amyloid precursor protein processing (schema) | 50 | 2.80E-16 | 4.09E-13 | 24 |
| 2 | Immune response_IFN-alpha/beta signaling via JAK/STAT | 62 | 9.39E-07 | 6.87E-04 | 16 |
| 3 | NETosis in SLE | 31 | 1.53E-06 | 7.47E-04 | 11 |
| 4 | Immune response_IFN-alpha/beta signaling via MAPKs | 73 | 2.05E-06 | 7.51E-04 | 17 |
| 5 | G-protein signaling_Rac1 activation | 74 | 1.13E-05 | 2.80E-03 | 16 |
| 6 | Oxidative stress_ROS signaling | 108 | 1.15E-05 | 2.80E-03 | 20 |
| 7 | Tau dysregulation in Alzheimer disease | 85 | 1.81E-05 | 3.80E-03 | 17 |
| 8 | Protein folding and maturation_Regulation of amyloid precursor protein processing | 108 | 3.99E-05 | 7.30E-03 | 19 |
| 9 | Immune response_Antimicrobial actions of IFN-gamma | 43 | 5.18E-05 | 8.42E-03 | 11 |
| 10 | Oxidative stress_Role of Sirtuin1 and PGC1-alpha in activation of antioxidant defense system | 60 | 7.28E-05 | 1.07E-02 | 13 |
| ***Enrichment by GO Processes*** | | | | | |
| 1 | response to stress | 5264 | 5.26E-39 | 5.42E-35 | 582 |
| 2 | response to organic substance | 4744 | 6.46E-38 | 2.88E-34 | 536 |
| 3 | response to external stimulus | 4115 | 8.39E-38 | 2.88E-34 | 483 |
| 4 | positive regulation of biological process | 8632 | 5.94E-36 | 1.25E-32 | 830 |
| 5 | metabolic process | 11081 | 6.07E-36 | 1.25E-32 | 1004 |
| 6 | organic substance metabolic process | 10486 | 1.64E-35 | 2.81E-32 | 961 |
| 7 | biological process involved in interspecies interaction between organisms | 2690 | 1.19E-33 | 1.74E-30 | 346 |
| 8 | response to biotic stimulus | 2498 | 3.94E-33 | 5.07E-30 | 327 |
| 9 | small molecule metabolic process | 2226 | 1.71E-32 | 1.96E-29 | 300 |
| 10 | response to other organism | 2417 | 3.33E-32 | 3.43E-29 | 317 |
| ***Enrichment by Process Networks*** | | | | | |
| 1 | Development_Regulation of angiogenesis | 221 | 1.47E-03 | 1.08E-01 | 33 |
| 2 | Development_Neurogenesis_Synaptogenesis | 179 | 1.63E-03 | 1.08E-01 | 28 |
| 3 | Proliferation_Negative regulation of cell proliferation | 183 | 2.28E-03 | 1.08E-01 | 28 |
| 4 | Development_Neurogenesis_Axonal guidance | 230 | 2.86E-03 | 1.08E-01 | 33 |
| 5 | Cell adhesion_Amyloid proteins | 197 | 3.45E-03 | 1.08E-01 | 29 |
| 6 | Cardiac development_FGF_ErbB signaling | 124 | 5.07E-03 | 1.17E-01 | 20 |
| 7 | Cell adhesion_Synaptic contact | 176 | 5.21E-03 | 1.17E-01 | 26 |
| 8 | Apoptosis_Apoptotic nucleus | 161 | 6.35E-03 | 1.25E-01 | 24 |
| 9 | Development_Blood vessel morphogenesis | 227 | 7.85E-03 | 1.34E-01 | 31 |
| 10 | Reproduction_Feeding and Neurohormone signaling | 210 | 8.55E-03 | 1.34E-01 | 29 |
| ***Enrichment by Disease (by Biomarkers)*** | | | | | |
| 1 | Lung Neoplasms | 20403 | 3.16E-66 | 9.46E-63 | 1463 |
| 2 | Respiratory Tract Neoplasms | 20512 | 8.07E-66 | 1.21E-62 | 1467 |
| 3 | Thoracic Neoplasms | 20431 | 1.24E-65 | 1.24E-62 | 1463 |
| 4 | Lung Diseases | 21289 | 2.26E-65 | 1.69E-62 | 1500 |
| 5 | Respiratory Tract Diseases | 21469 | 4.98E-65 | 2.99E-62 | 1507 |
| 6 | Neoplasms by Site | 25780 | 1.69E-49 | 8.42E-47 | 1644 |
| 7 | Urogenital Neoplasms | 20843 | 3.06E-46 | 1.31E-43 | 1437 |
| 8 | Neoplasms | 26380 | 3.38E-45 | 1.27E-42 | 1657 |
| 9 | Urogenital Diseases | 21425 | 9.79E-45 | 3.26E-42 | 1460 |
| 10 | Female Urogenital Diseases | 19789 | 3.50E-40 | 1.05E-37 | 1370 |

| **Table S3. Network significance of process network in *Mir137*-/- vs. *Mir137*+/+** | | |
| --- | --- | --- |
| Ranking | Process | FDR adjusted p-value |
| ***miR-137 regulated network*** | | |
| 1 | regulation of biological quality | 2.60E-40 |
| 2 | behavior | 1.50E-32 |
| 3 | positive regulation of cytosolic calcium ion concentration | 9.63E-32 |
| 4 | cellular response to chemical stimulus | 1.18E-30 |
| 5 | signaling | 1.41E-30 |
| 6 | cell communication | 3.09E-30 |
| 7 | synaptic signaling | 4.99E-30 |
| 8 | cell-cell signaling | 1.60E-29 |
| 9 | trans-synaptic signaling | 3.31E-29 |
| 10 | nervous system development | 2.10E-28 |
| ***IGF-1-centered regulated network*** | | |
| 1 | regulation of cell population proliferation | 6.82E-211 |
| 2 | positive regulation of cell population proliferation | 2.00E-136 |
| 3 | positive regulation of cellular process | 3.19E-107 |
| 4 | cell surface receptor signaling pathway | 1.54E-103 |
| 5 | negative regulation of cell population proliferation | 9.25E-103 |
| 6 | positive regulation of biological process | 2.84E-101 |
| 7 | negative regulation of biological process | 3.23E-96 |
| 8 | negative regulation of cellular process | 6.49E-96 |
| 9 | response to endogenous stimulus | 4.19E-95 |
| 10 | regulation of phosphorylation | 2.68E-90 |

**Table S4. Gene list of up-regulated and secreted proteins^1^ in the brain of miR-137 deficient mice.**

| Gene | Probe ID | Fold Changes | GO Molecular Function | Potential miR-137 target^2^ |
| --- | --- | --- | --- | --- |
| *Fndc5* | 1453135_at | 1.16 | hormone activity/inferred from electronic annotation; protein binding/inferred from physical interaction | Yes |
| *Wfikkn2* | 1428811_at | -1.02 | enzyme inhibitor activity/inferred from electronic annotation; serine-type endopeptidase inhibitor activity/inferred from electronic annotation; protein binding/inferred from physical interaction; metalloendopeptidase inhibitor activity/inferred from electronic annotation; peptidase inhibitor activity/inferred from electronic annotation | No |
| *Fam3c* | 1448904_at | 1.50 | protein binding/inferred from electronic annotation | Yes |
| *Fgf9* | 1438718_at | 1.58 | receptor binding/inferred from electronic annotation; fibroblast growth factor receptor binding; growth factor activity; heparin binding/inferred from electronic annotation | Yes |
| *Slc8a3* | 1450311_at | 1.01 | calcium: sodium antiporter activity; calcium: sodium antiporter activity; calcium: cation antiporter activity | No |
| *Cxadr* | 1453282_at | 1.93 | receptor binding; integrin binding; protein binding; beta-catenin binding; PDZ domain binding; identical protein binding; cell adhesion molecule binding; connexin binding; cell adhesive protein binding involved in AV node cell-bundle of His cell communication | Yes |

^1^Predicted by SignalIP website and Metacore software.

^2^Predicted by miRWalk website.

**Table S5. Primer sequences for genotyping.**

| Primer name | 5’-3’ sequence |
| --- | --- |
| *miR-137* WT Forward (I2U) | GTACTGAAAGGGTGGGCTG |
| *miR-137* WT Reverse (I2D) | TGCTCTATTGCTTCCTGCC |
| *miR-137* KO Forward (I1U) | GATGAGTGGTGGTGGTGGGAG |
| *miR-137* KO Reverse (I3D) | AGAGGTCCGCTCACAGCTAGG |

**Table S6. Primer sequences for Real-time QPCR**

| *Gene* | Forward primer sequence (5’-3’) | Reverse primer sequence (5’-3’) |
| --- | --- | --- |
| *Runx2* | CCTAGTTAGAGTGGTAGCAGAAGC | ACAGACAACGAAGAAAGTTCCCAC |
| *Ibsp* | AAGCAGCACCGTTGAGTATGG | CCTTGTAGTAGCTGTATTCGTCCTC |
| *Sp7* | ACTCATCCCTATGGCTCGTG | GGTAGGGAGCTGGGTTAAGG |
| *Alpl* | TAACACCAACGCTCAGGTCC | TGGATGTGACCTCATTGCCC |
| *Col1a1* | GGAGAGAGCATGACCGATGG | CGATCTCGTTGGATCCCTGG |
| *Asc1* | GGGTGGCACTCAAGAAAGAG | AGTGTTCCAGGACACCCTTG |
| *Ucp1* | GGCCTCTACGACTCAGTCCA | TAAGCCGGCTGAGATCTTGT |
| *Cd137* | CCTGTGATAACTGTCAGCCTG | TCTTGAACCTGAAATAGCCTGC |
| *Tbx1* | TGGGACGAGTTCAATCAGC | TGTCATCTACGGGCACAAAG |
| *Cidea* | TGACATTCATGGGATTGCAGAC | GGCCAGTTGTGATGACTAAGAC |
| *Cox7a* | GCTCTGGTCCGGTCTTTTAGC | GTACTGGGAGGTCATTGTCGG |
| *Cox8b* | TGTGGGGATCTCAGCCATAGT | AGTGGGCTAAGACCCATCCTG |
| *Slc2a1* | GGACCCTGCACCTCATTG | GGCCACGATGCTCAGATAG |
| *Slc2a4* | CATTCCCTGGTTCATTGTGG | GAAGACGTAAGGACCCATAGC |
| *Igf1* | AGACAGGCATTGTGGATGAG | TGAGTCTTGGGCATGTCAGT |
| *Igfbp3* | AATGGCCGCGGGTTCTGC | TTCTGGGTGTCTGTGCTTTGAG |
| *Igfals* | AGCTCAGCGTCTTTTGCAGT | ACAGGTTGTTTCCGTCAAGC |
| *Socs2* | TCCCTCGTCTTATGCAACTAATC | GGATCTCATGGTGATGGTTCTT |
| *Igf1r* | GTGGGGGCTCGTGTTTCTC | GATCACCGTGCAGTTTTCCA |
| *Gh* | AGGCCCAGCAGAGAACCGACA | ACGGTCCGAGGTGCCGAACA |
| *Tshb* | GGGCAAGCAGCATCCTTTTG | GTGTCATACAATACCCAGCACAG |
| *Pomc* | ATGCCGAGATTCTGCTACAGT | TCCAGCGAGAGGTCGAGTTT |
| *Ghrh* | GCTGTATGCCCGGAAAGTGAT | AATCCCTGCAAGATGCTCTCC |
| *Ghrhr* | GCGGAGTTACACTGGGTCAC | ATGCAAGCTCATCGTCTCTCA |
| *Sst* | GAGCCCAACCAGACAGAGAA | GAAGTTCTTGCAGCCAGCTT |
| *Sstr1* | CTACTGTCTGACTGTGCT | ATGGGCAAGATAACCAGTAAT |
| *Sstr2* | CTGGAATCCGAGTGGGATCAT | ACGGAAGAGACGTTGAAGATGTA |
| *Sstr3* | GGCATCAACCAGTTCACCAG | GAGAACACAACCACAGGCAG |
| *Sstr4* | GCCAAGCTAATCAACCTGGG | AGCATAATCCGATGGCCAGA |
| *Sstr5* | GGCTTCCACACCTAGCTGG | AGCACAGGCACTAATACCGC |
| *Ghr* | GATTTTACCCCCAGTCCCAGTTC | GACCCTTCAGTCTTCTCATCCACA |
| *Pdk4* | CAAAGACGGGAAACCCAAGCC | CGCAGAGCATCTTTGCACAC |
| *Ldhb* | CATTGCGTCCGTTGCAGATG | GGAGGAACAAGCTCCCGTG |
| *Pfk1* | TGTGGTCCGAGTTGGTATCTT | GCACTTCCAATCACTGTGCC |
| *G6pc* | GTGGCAGTGGTCGGAGACT | ACGGGCGTTGTCCAAAC |
| *Pck1* | CACCATCACCTCCTGGAAGA | GGGTGCAGAATCTCGAGTTG |
| *Hamgcs2* | CCGTATGGGCTTCTGTTCAG | AGCTTTGTGCGTTCCATCAG |
| *Sirt1* | TTGGCACCGATCCTCGAAC | CCCAGCTCCAGTCAGAACTAT |
| *Fgf21* | CCTCTAGGTTTCTTTGCCAACAG | AAGCTGCAGGCCTCAGGAT |
| *Htr1f* | TTCTGGAGGCACCAAGGAAC | GTCCGTTGATGGATCGGACA |
| *Slc25a5* | AGGGCATCATAGACTGCGTG | CTGGGTCCTCTTGTCCACAC |
| *Dclk1* | AGCAACCACCGCTCTTGATA | GGGGGAGTAGTCCTCCGATT |
| *Foxp1* | TGCAAAAGACAAAGAGCGCC | CTGCTGAAGAAATGGGCACG |
| *Zfp804a* | CAAAGTGCTTCAGCCACACC | GGGGTAAGGGAGGAAAAGCC |
| *Tcf4* | GACTCGCCAGGCTATCCTTC | ACTGGAGTTGATGTCTGCCG |
| *Bcl11a* | AACCATTCCAGCCAGGTAGC | ATGGGGGACGATTTGTGCAT |
| *Syt1* | TCATCCAGGCTGCTGAACTG | CACAGCCATCACCAGTGTCT |
| *Grin2a* | GAACTACAAGGCCGGGAGG | TTGTGGCAGATGCCCGTAAG |
| *Cadm2* | CGGTTCTCCGCTTCTACAGT | ACCAGCTCGATCCGATTGTC |
| *Gabra1* | GGTTGACCGTGAGAGCTGAA | CTACAACCACTGAACGGGCT |
| *Epha7* | CATCTGGCTGCTTGGCTTTG | CCTGGCACACCTGGTATGTT |
| *Ncoa2* | TGGAGGATCCTGGTCTGGAG | CGTGCCACACAAATCAAGCA |
| *Satb2* | TTTGCGAACTGCTTCGTTGG | TTAGTTGGCTGGGACTGCTG |
| *Ptpn2* | GGCCAACGGATGACAGAGAA | GGTCAGGGGTCAAACAACCA |
