# Supplementary material for: Brain miR-137 governs growth and development via GH/IGF-1 signaling
Source: BMC Biol. 2025 Jul 1;23:197. doi: 10.1186/s12915-025-02306-8 (PMC12219031; doi:10.1186/s12915-025-02306-8)

Fig. 1B Southern Blot

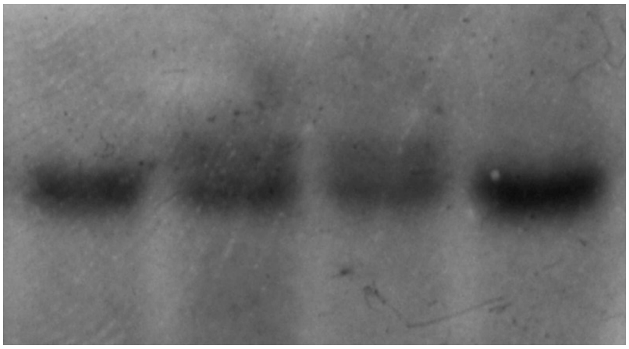

Fig. 3C Western Blot

IGF-1

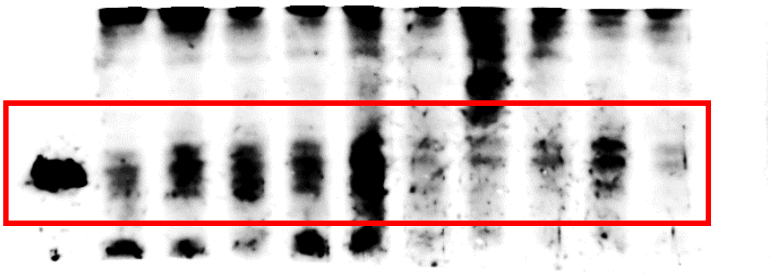

GAPDH

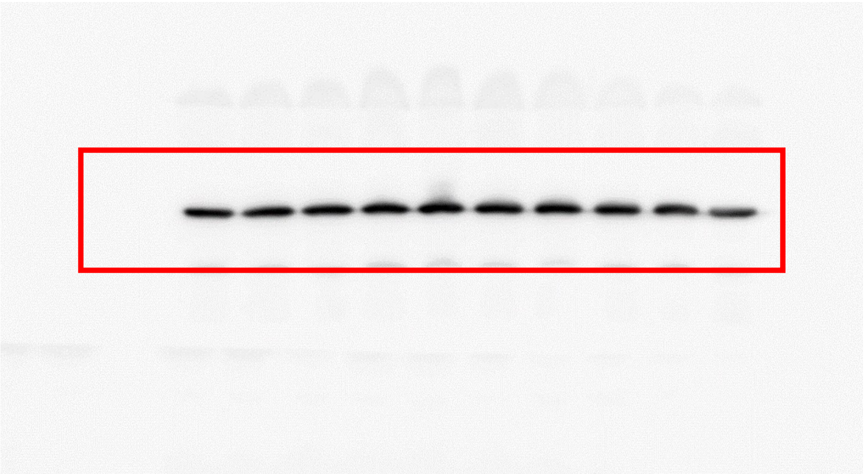

Fig. 4A Western Blot

p-STAT5

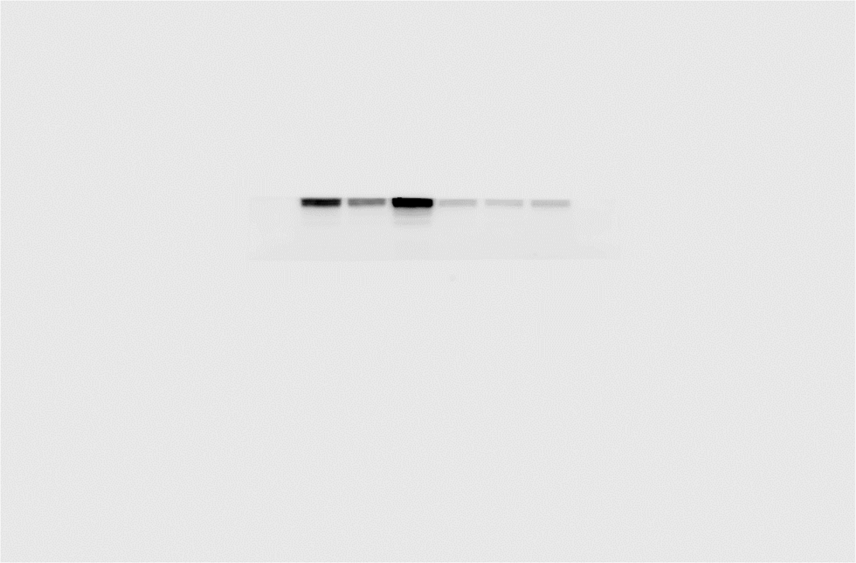

t-STAT5

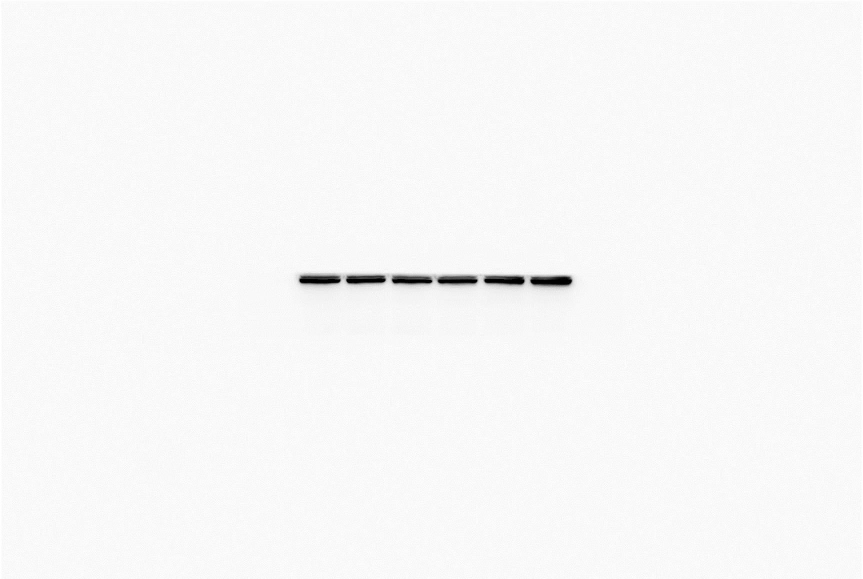

GAPDH

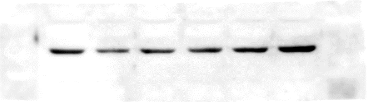

Fig. 4B Western Blot

p-STAT5

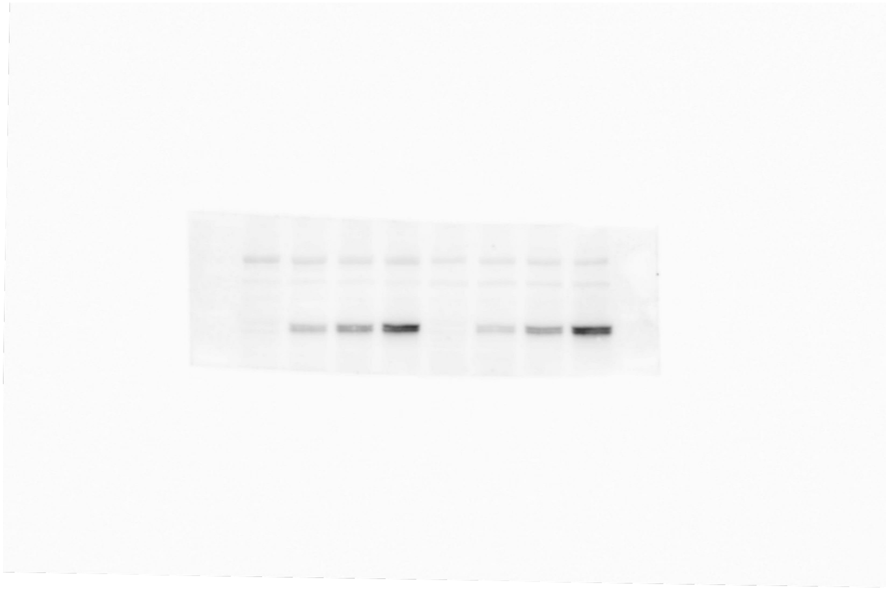

t-STAT5

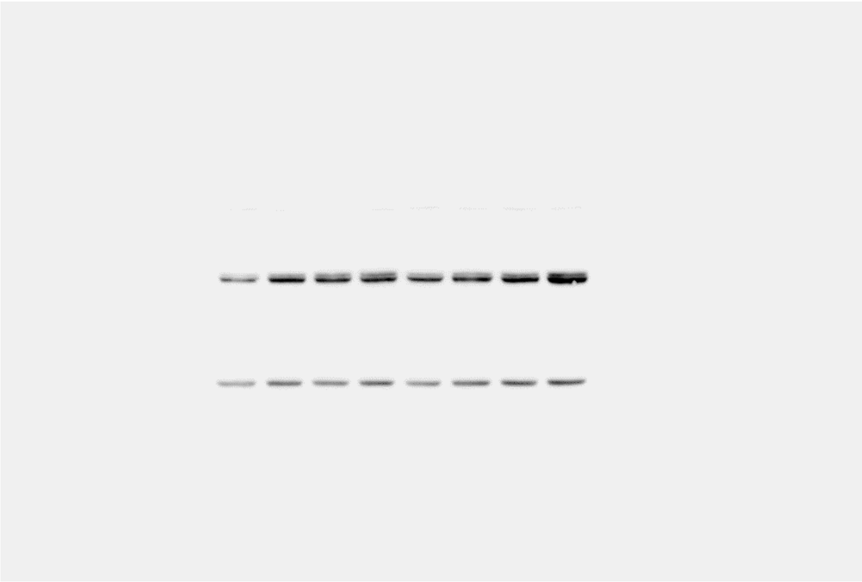

GAPDH

Fig. 4G Western Blot

p-STAT5

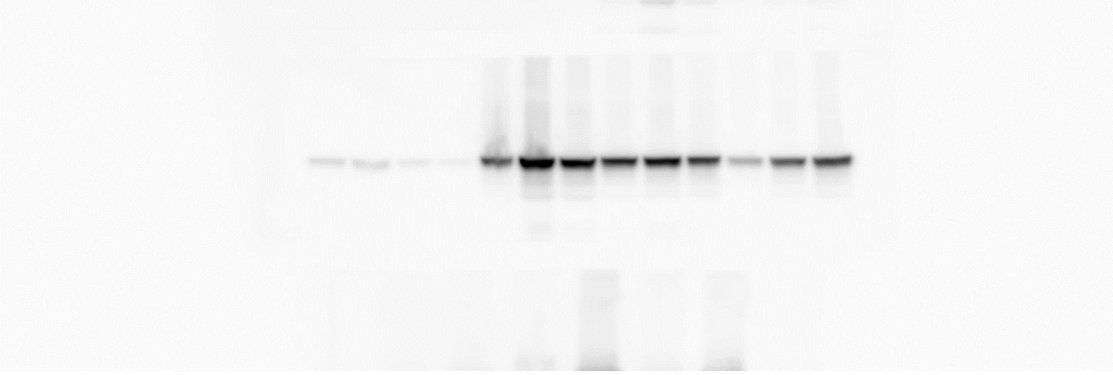

t-STAT5

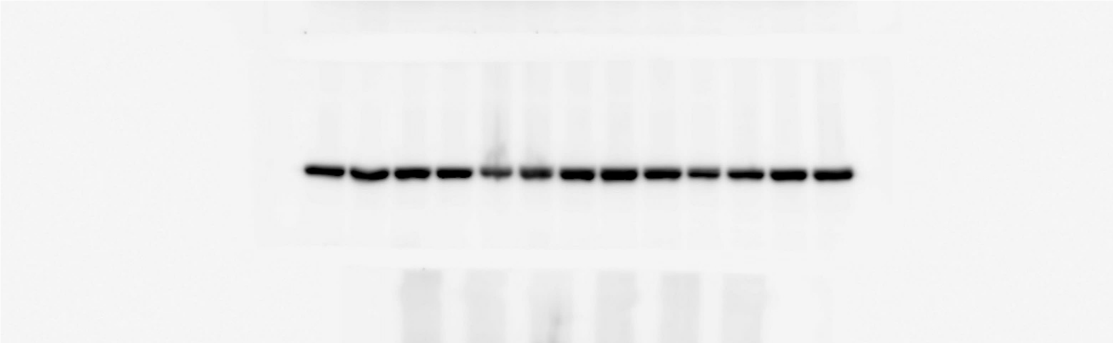

GAPDH

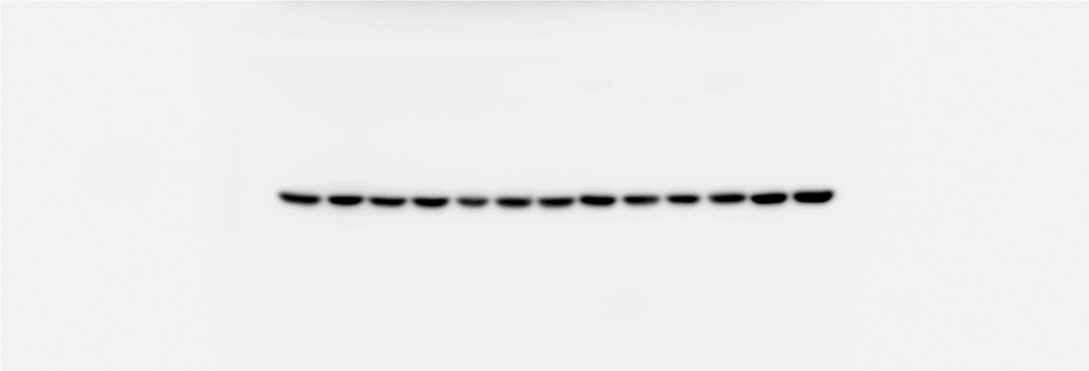

Fig. 4I Western Blot (left)

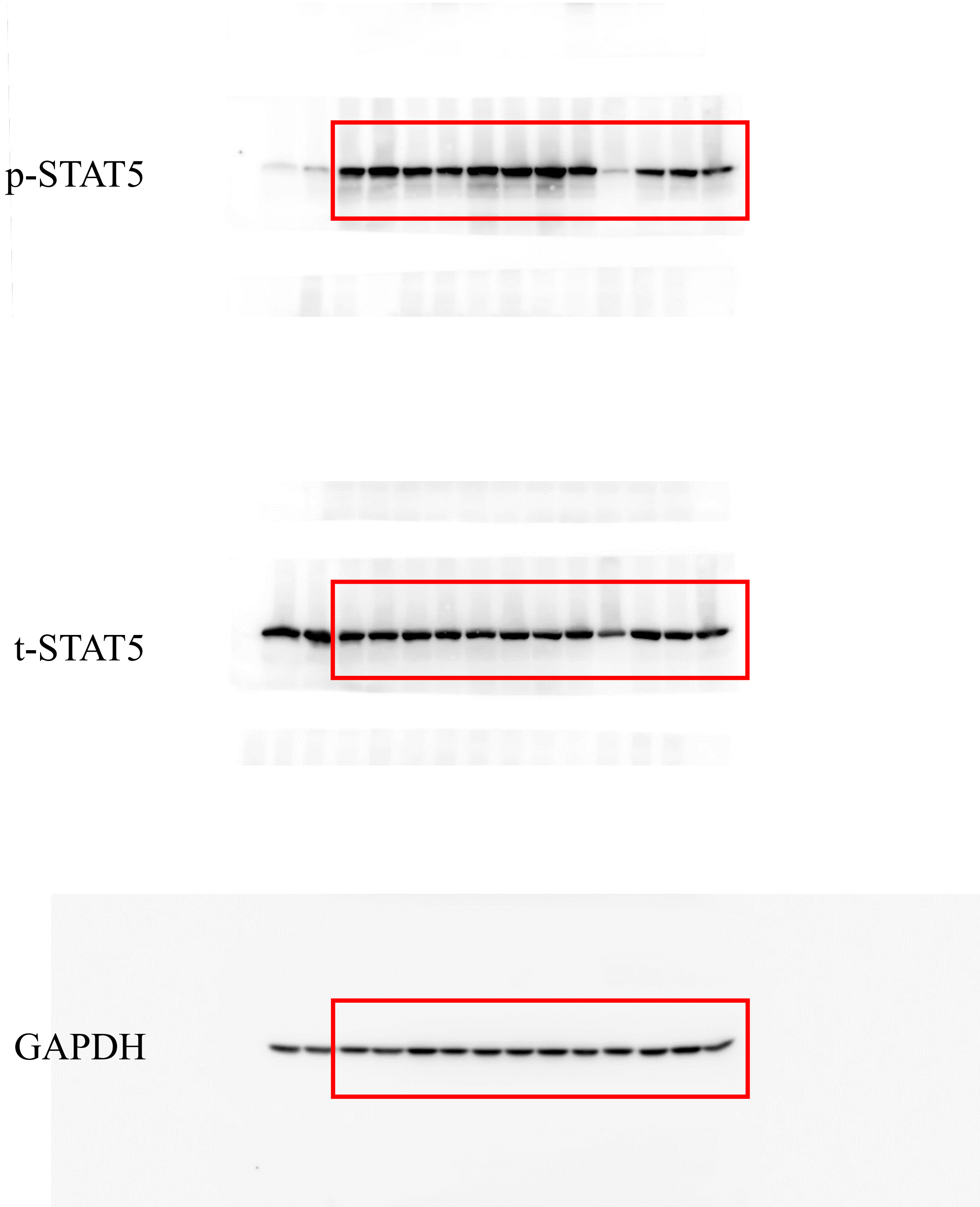

Fig. 4I Western Blot (right)

p-STAT5

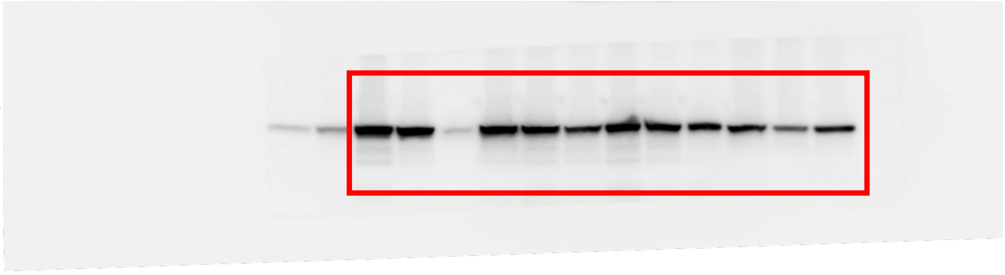

t-STAT5

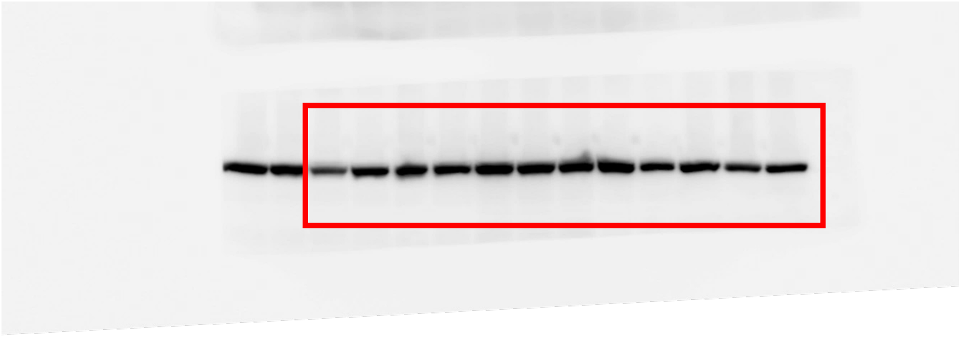

GAPDH

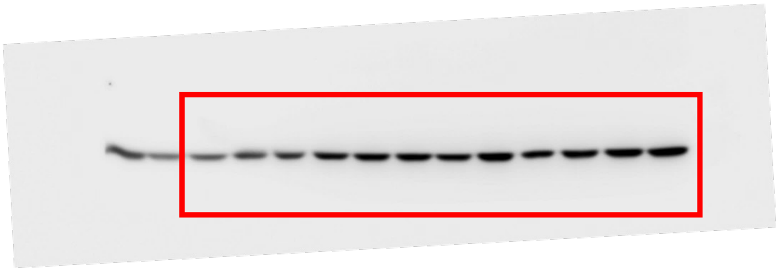

Fig. 5E Western Blot

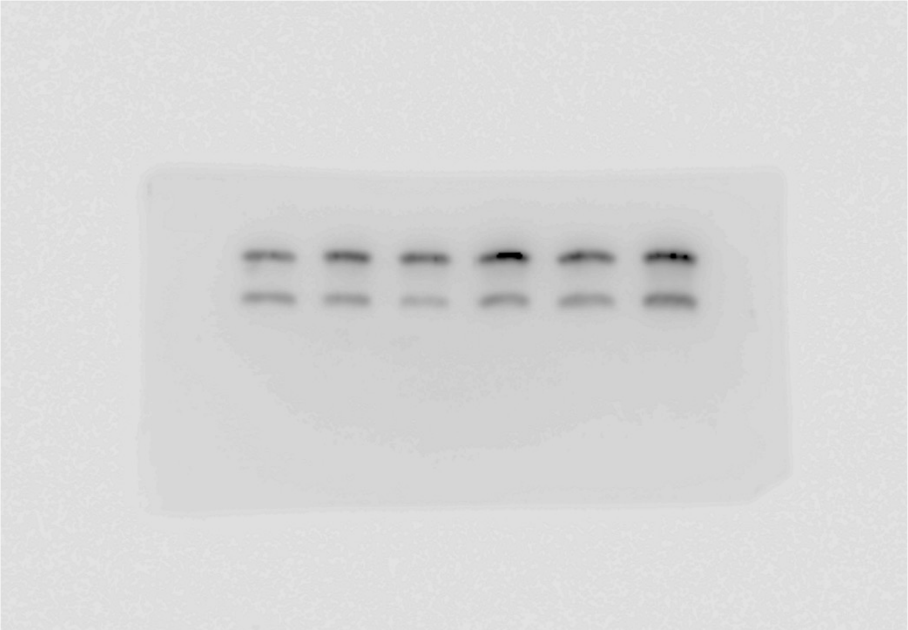

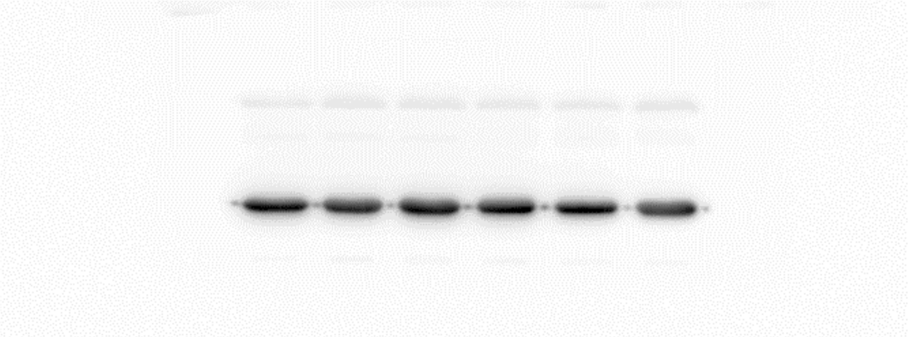

Fig. 6B mouse images

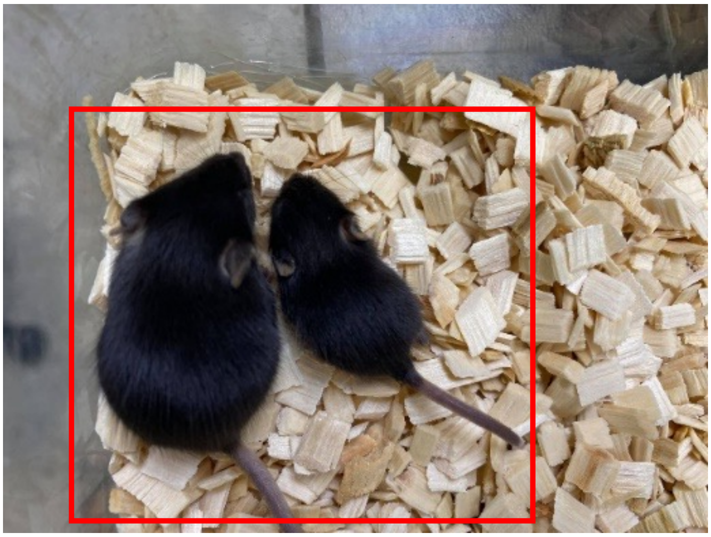

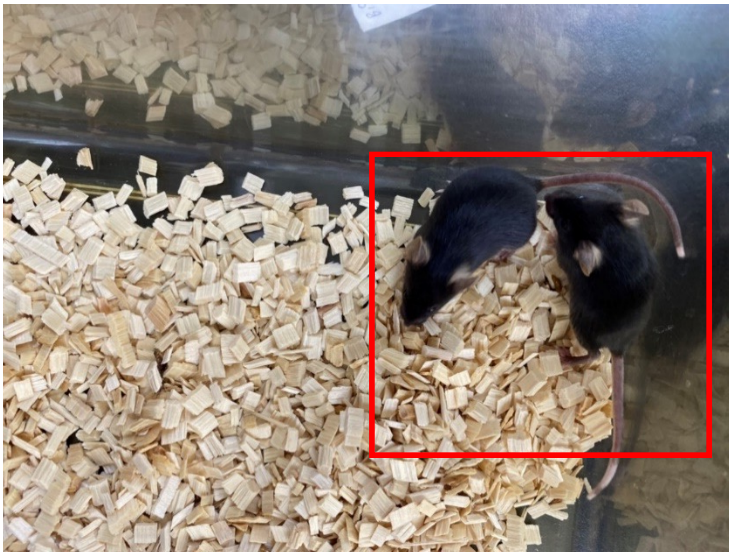

Fig. 6C Western Blot (Nestin-Cre)

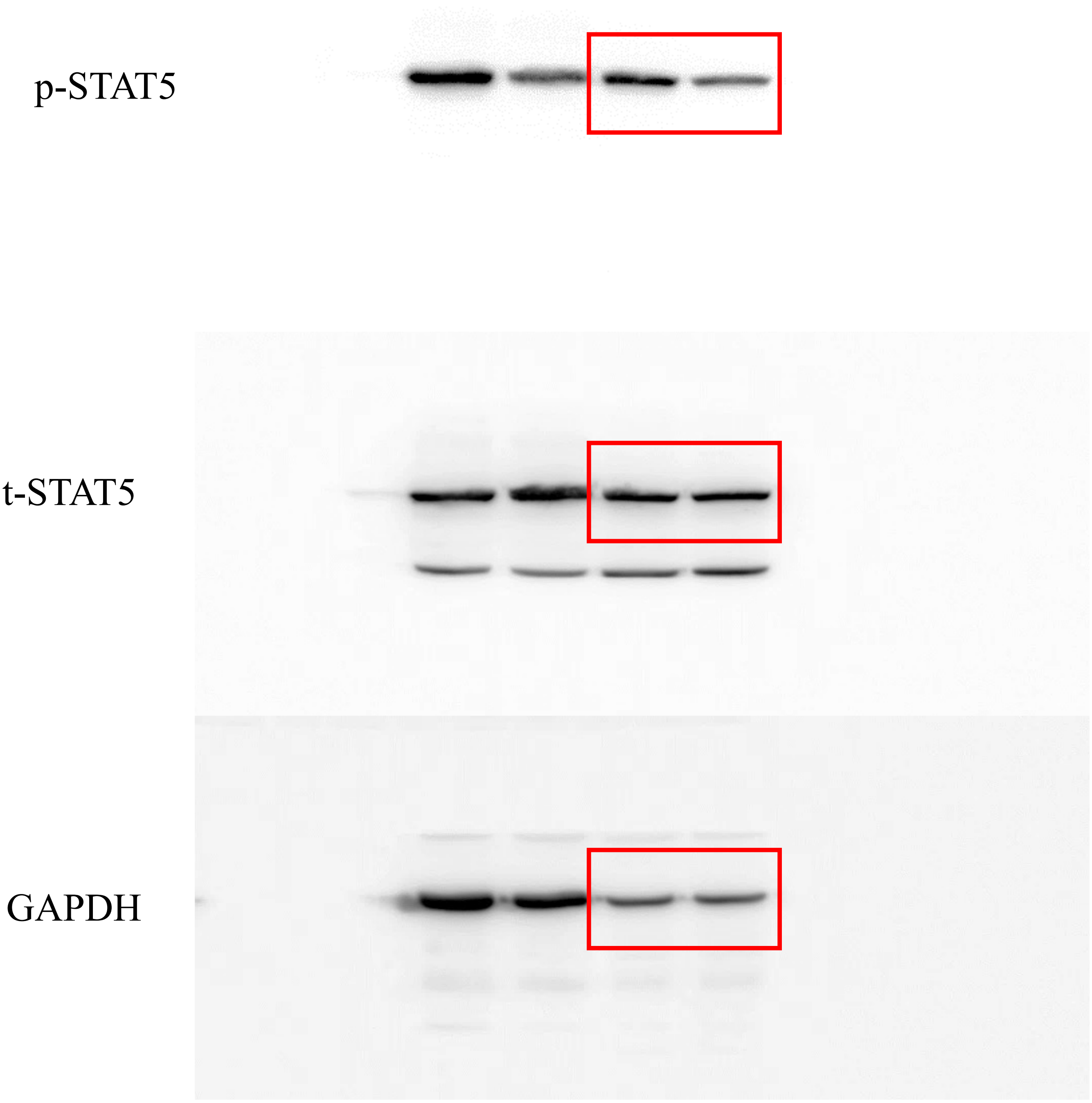

Fig. 6C Western Blot (Albumin-Cre)

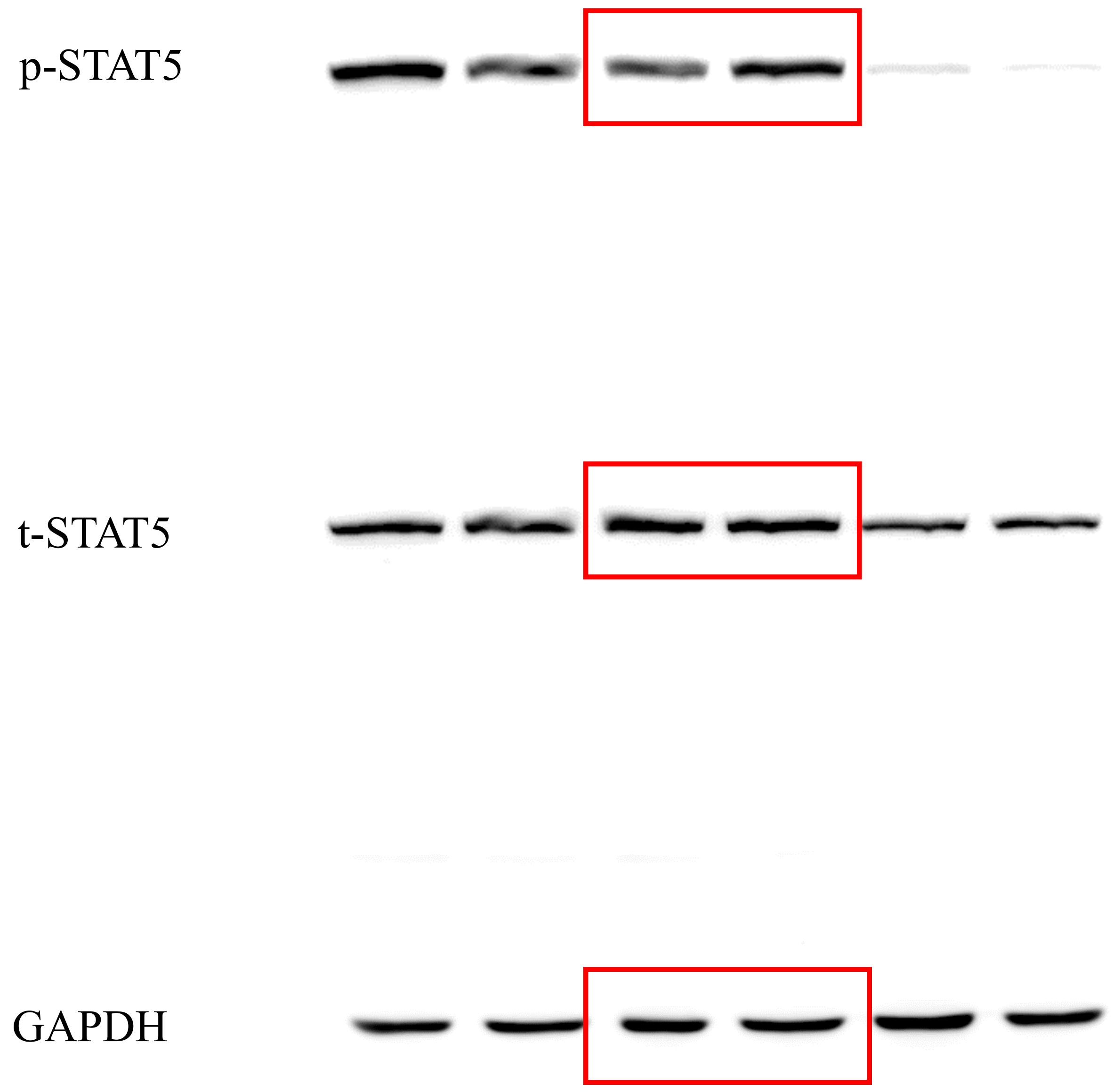

Fig. S4 Muscle Western Blot

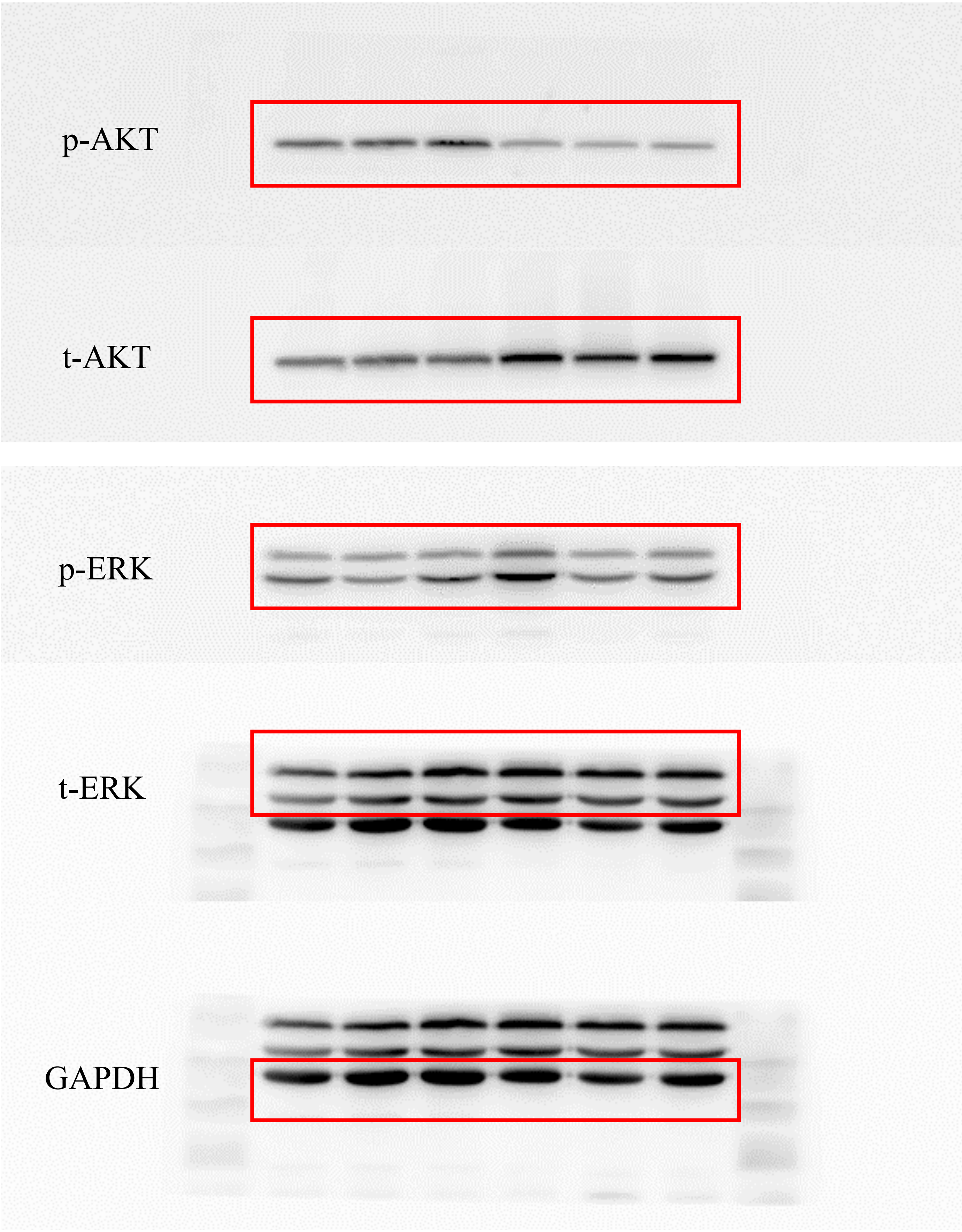

Fig. S4 Lung Western Blot

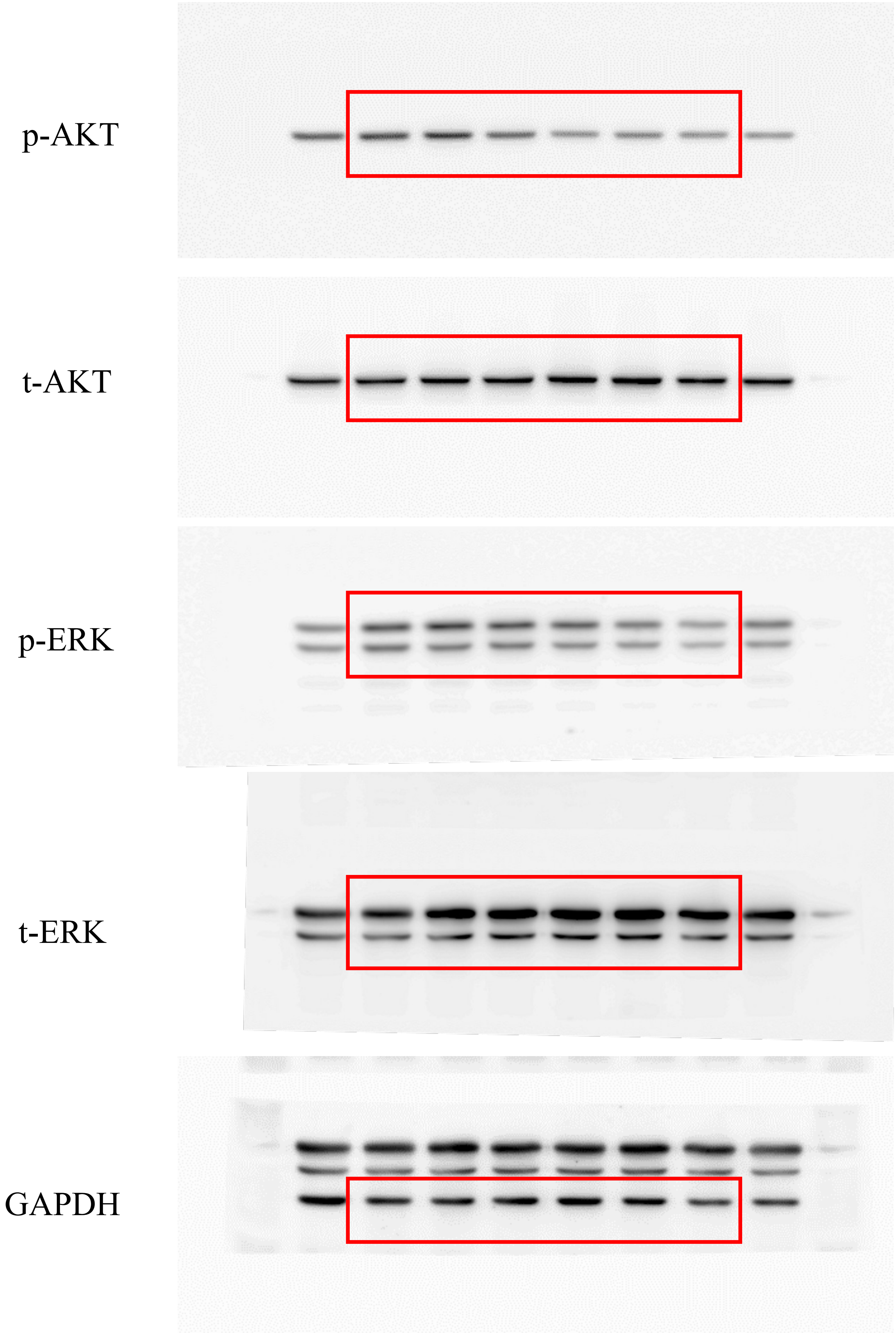

Fig. S4 Heart Western Blot

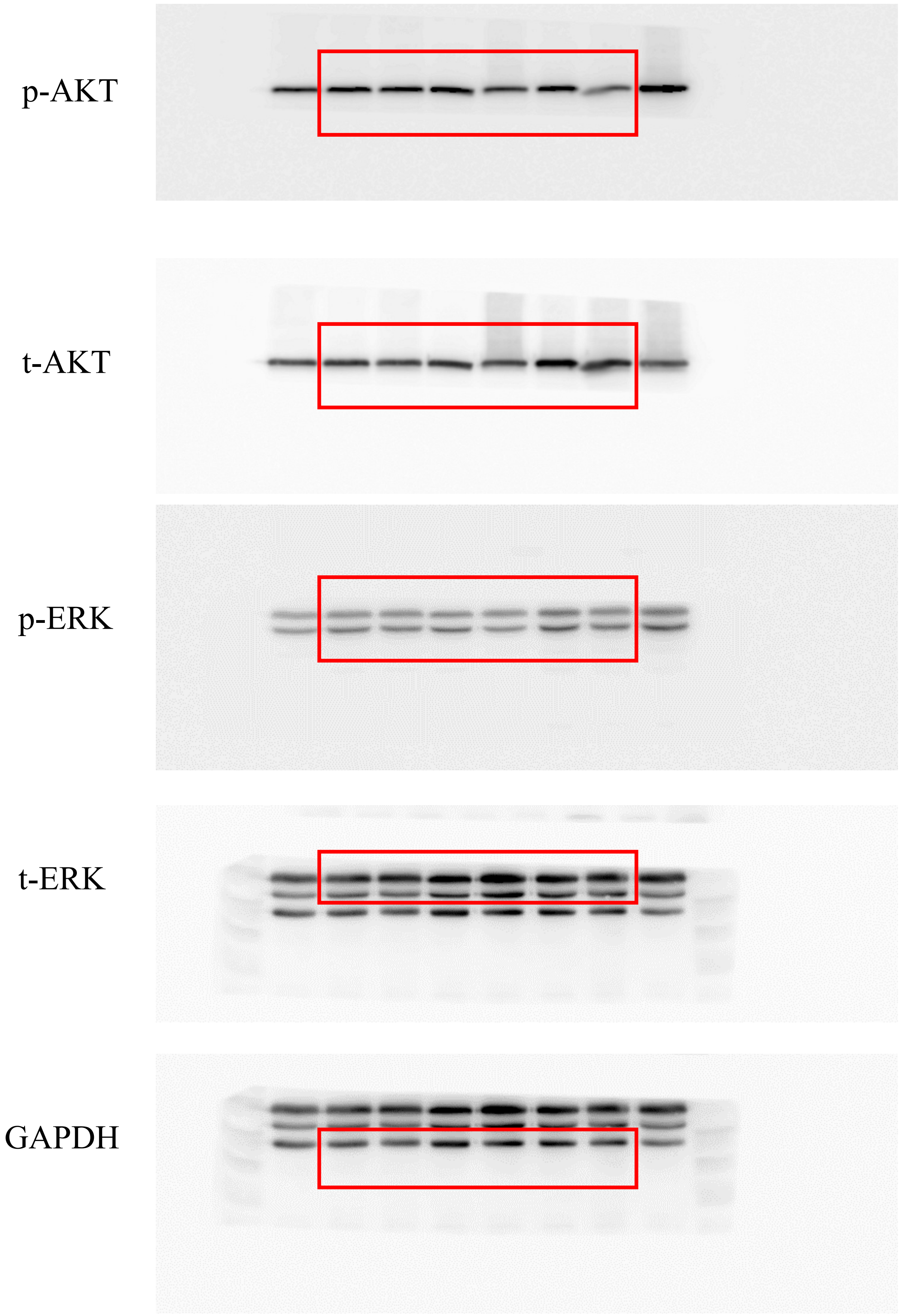

Fig. S4 Brain Western Blot

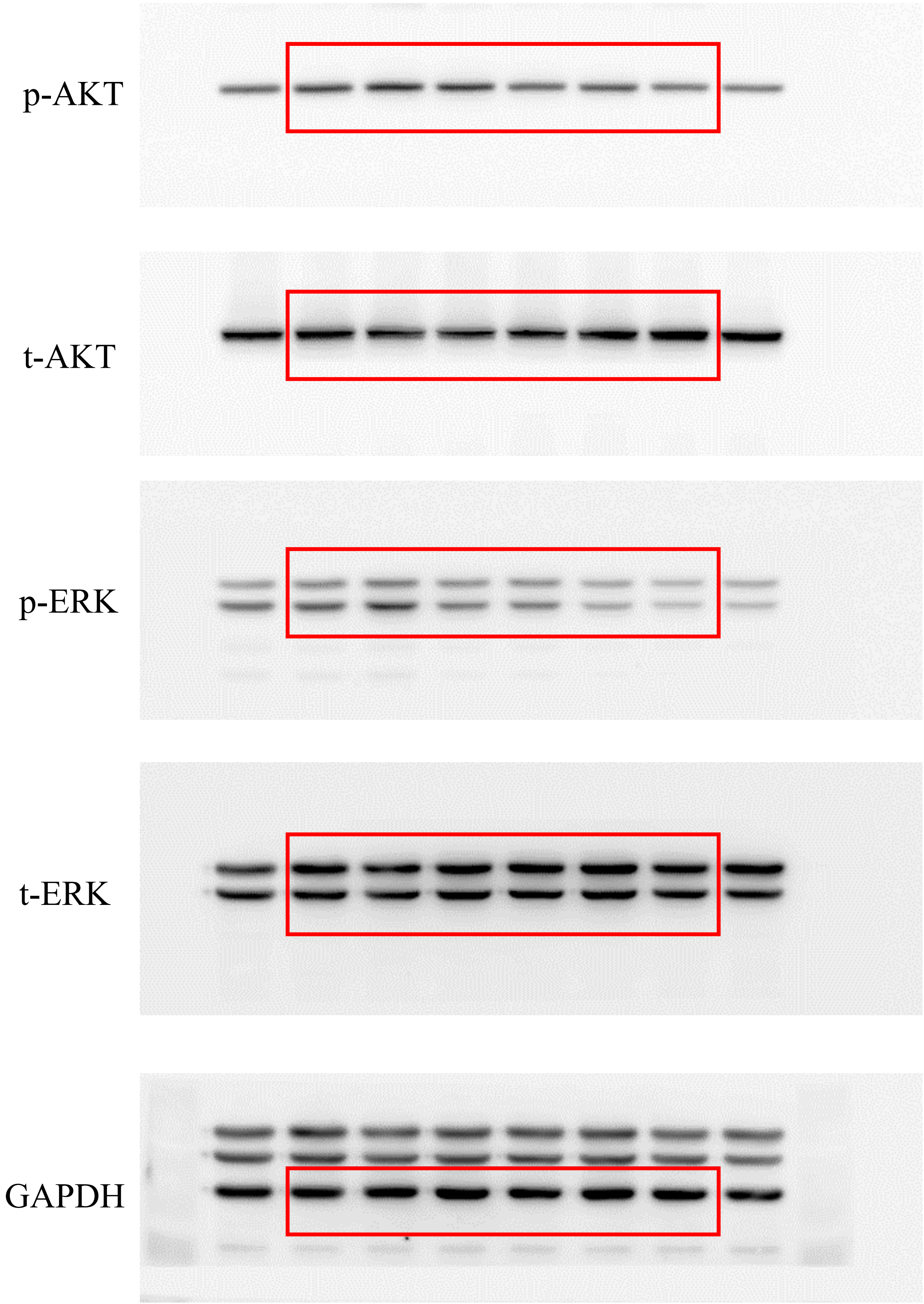

Fig. S6 Western Blot

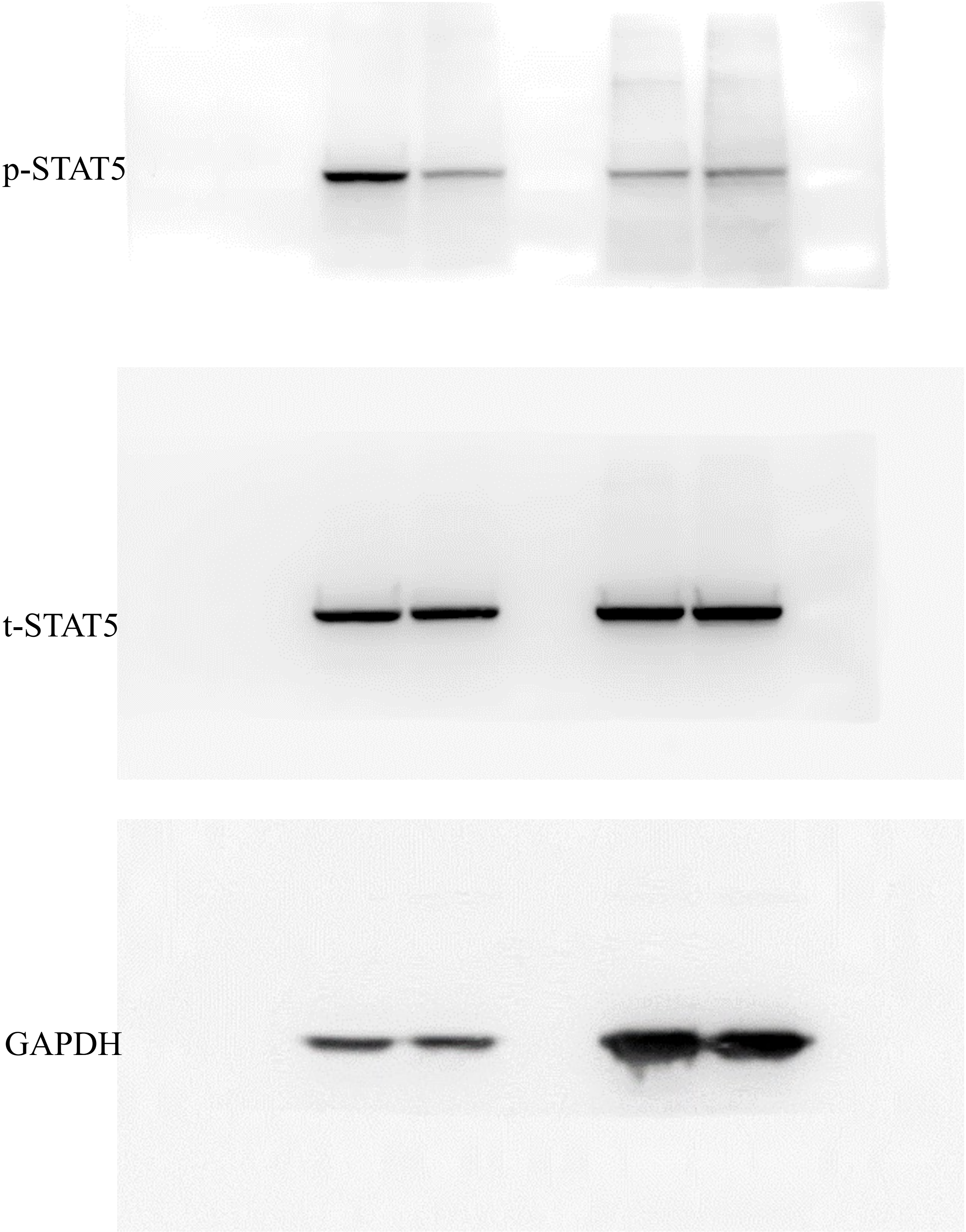

Fig. S12A Western Blot

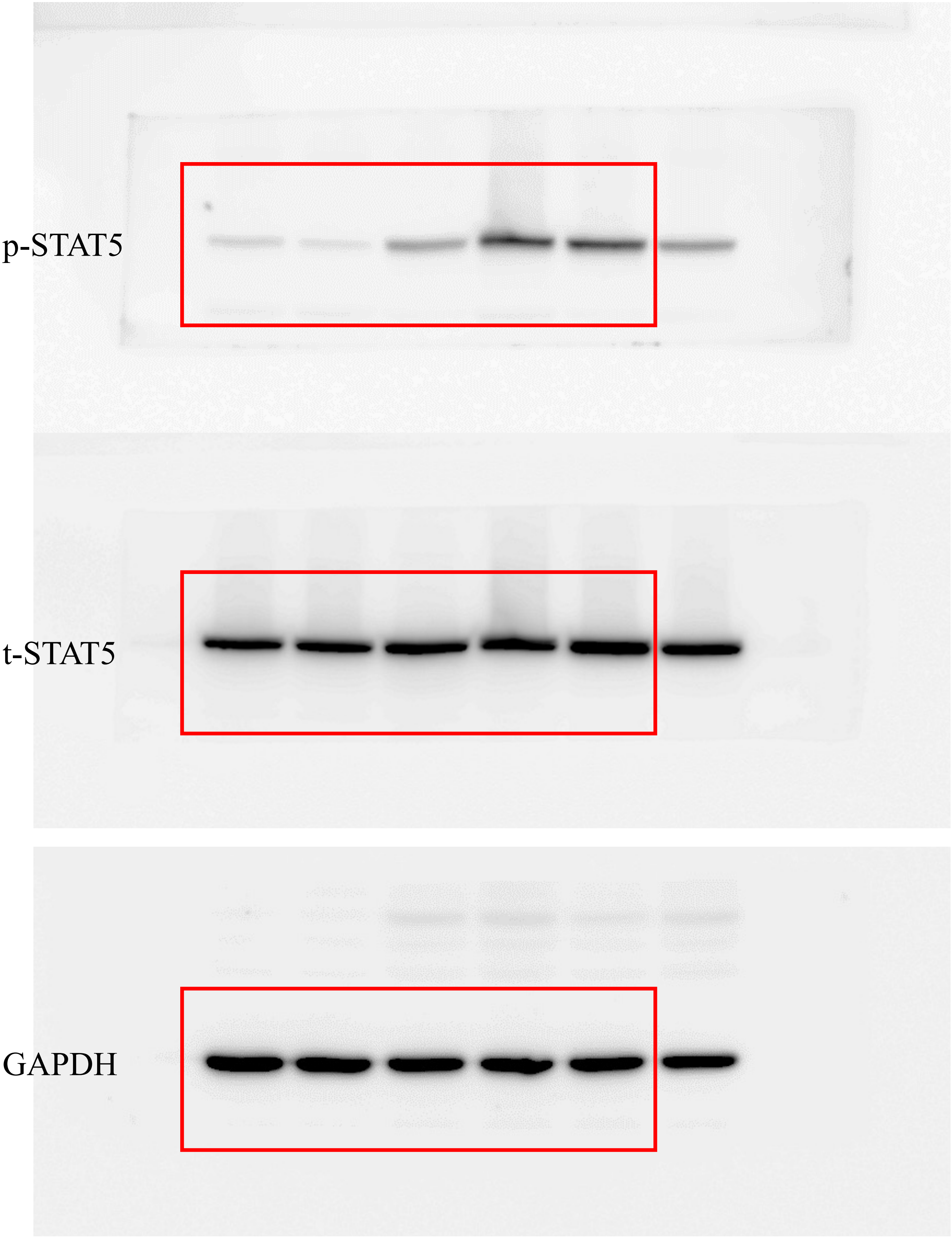

Fig. S13 Western Blot

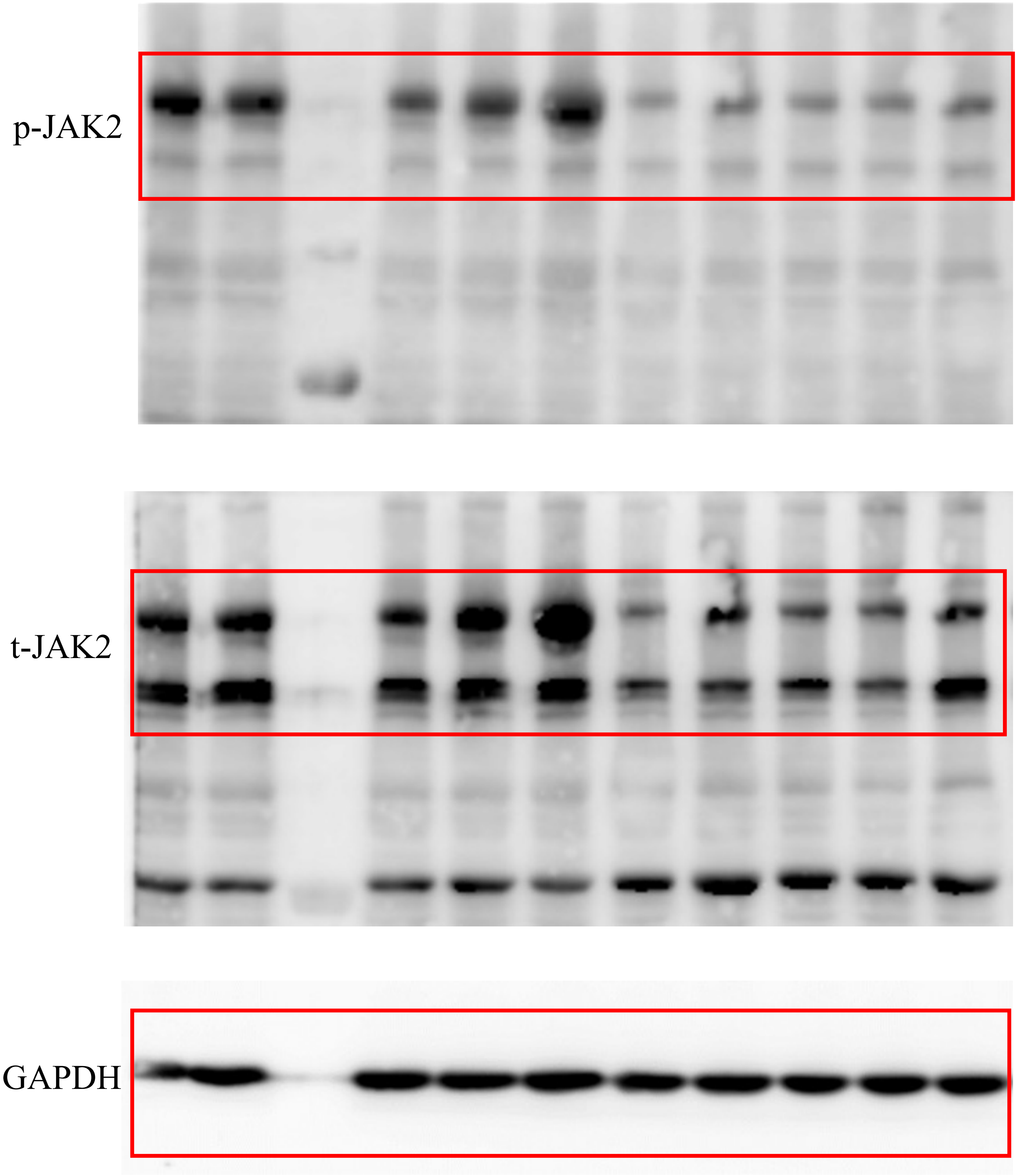

Supplement: Supplementary file 3 — Additional file 3. [file 12915_2025_2306_MOESM3_ESM.pdf]
